# Investigating the functional role of SETD6 in lung adenocarcinoma

**DOI:** 10.1186/s12885-022-10476-9

**Published:** 2023-01-06

**Authors:** Jing Xu, Hui Zhou, Ziling Luo, Jie Chen, Man Liu

**Affiliations:** 1 Department of Pharmacy, Dermatology Hospital, Southern Medical University, Guangzhou, China; 2 Department of Pharmacy, the First Affiliated Hospital of Sun Yat-Sen University, Guangzhou, China; 3 Department of Pharmacy, the Third Affiliated Hospital of Southern Medical University, Guangzhou, China; 4 Department of Gastroenterology, the First Affiliated Hospital of Sun Yat-Sen University, Guangzhou, China

**Keywords:** SET domain containing 6, Lung adenocarcinoma, Nuclear factor-κB, Nuclear factor erythroid 2–related factor 2

## Abstract

**Background:**

SET domain containing 6 (SETD6) has been shown to be upregulated in multiple human cancers and can promote malignant cell survival. However, expression and function of SETD6 in lung adenocarcinoma (LUAD) remains unaddressed. This study aimed to demonstrate the expression pattern, biological roles and potential mechanisms by which SETD6 dysregulation is associated with LUAD.

**Methods:**

The expression level of SETD6 was evaluated in LUAD clinical specimens and its correlation with clinical parameters were analyzed. In vitro, gain-of-function and loss-of-function experiments were performed to evaluate the effects of SETD6 on cell proliferation, apoptosis, migration, and colony formation of LUAD cell line A549. Western-blot was performed to investigate the involvement of nuclear factor-κB (NF-κB) and nuclear factor erythroid 2–related factor 2 (Nrf2) pathways as downstream signaling of SETD6 in LUAD cells.

**Results:**

Compared with non-tumorous tissues, SETD6 was overexpressed in tumor tissues, and its overexpression significantly correlates with higher rates of regional lymph node metastasis and poor prognosis in patients with LUAD. In A549 cell line, SETD6 overexpression could promote cell proliferation, migration, colony formation and inhibit cell apoptosis, whereas SETD6 knockdown caused the opposite effects. Furthermore, we demonstrated that the mechanisms underlying the effect of SETD6 on LUAD biological behaviors may be through its interaction with NF-κB and Nrf2 signaling pathways.

**Conclusions:**

SETD6, which is highly expressed in LUAD tumor tissues, plays an important role in promoting the malignant behaviors of LUAD via likely the NF-κB and Nrf2 signaling pathways.

**Supplementary Information:**

The online version contains supplementary material available at 10.1186/s12885-022-10476-9.

## Background

Lung adenocarcinoma (LUAD) is the most prevalent type of lung cancer, accounting for over 40% of all types of lung cancer [1]. Although there has been great progress in new treatment regimens in the last few decades, the 5-year survival rate for patients with LUAD remains poor, which is less than 20% [2]. Therefore, investigating novel genes and elucidating their regulatory pathways that involved in the development of LUAD is critical to identify novel prognostic biomarkers, and design rational therapeutic targets for patients with LUAD.

The SET-domain-containing protein 6 (SETD6) is a member of the protein lysine methyltransferases (PKMTs), which is a key player in regulating gene translation, intracellular signaling pathways and involved in cell mitosis, proliferation, inflammatory processes and oxidative stress [3–7]. Previous studies showed SETD6 is overexpressed in multipe human cancers and participates in tumorigenesis [8–10]. For instance, SETD6 can promote breast cancer cell proliferation and migration [8]. SETD6 also increases viability and proliferation of transformed bladder cells [9]. Silencing of SETD6 can inhibit the tumorigenesis of oral squamous cell carcinoma [10]. However, to the best of our knowledge, the expression pattern and functional role of SETD6 in LUAD remains unaddressed.

In this study, we identified SETD6 as a significantly upregulated protein in LUAD tissues, and SETD6 overexpression significantly correlated with a higher rate of regional lymph node metastasis and poor prognosis in patients with LUAD. We also delineated for the first time the function of SETD6 as a novel regulator of cell proliferation, migration, colony formation and cell apoptosis in LUAD cells. Further mechanistic studies uncovered that the biological function of SETD6 in LUAD may associate with its interaction with downstream nuclear factor-κB (NF-κB) and nuclear factor erythroid 2–related factor 2 (Nrf2) pathways.

## Materials and methods

### Human tissue collection

Paraffin-embedded tissue samples including tumor and adjacent tissues of LUAD patients were collected from the First Affiliated Hospital, Sun Yat-sen University (Guangzhou, China) from December 2010 to December of 2020. Patient inclusion criteria of this study included: (1) Eligible patients underwent radical resection of lung cancer, which were confirmed by pathologists as LUAD; (2) Patients had not undergone radiotherapy or chemotherapy previously; (3) Patients had complete clinical data recorded. Patient exclusion criteria included: (1) Patients with LUAD that cannot be confirmed by postoperative pathology; (2) Patients had other cancer history before operation. Informed consents were obtained from all subjects and/or their legal guardian(s). All methods were carried out in accordance with relevant guidelines and regulations.

The diagnosis of tumor-node-metastasis (TNM) staging is based on the 8^th^ edition of International Association for the study of lung cancer (IASLC) staging standard in 2016 [11]. All specimens were histologically analyzed and classified by professional pathologists. Written informed consents were obtained from all patients for the use of their tissues and clinical data. The present study was approved by constituted ethical committee of The First Affiliated Hospital, Sun Yat-sen University. In total, 45 patients (29 males and 16 females) were included, with a median age of 65 years (range, 28 – 88 years). Among them, 9 were in stage I, 16 were in stage II, 13 were in stage III and 7 was in stage IV. We performed western blot assay and IHC analysis with the tumor and adjacent tissue samples to examine the expression pattern of SETD6 in LUAD. Correlations between SETD6 and clinical parameters were analyzed.

### Immunohistochemistry

Paraffin-embedded tissue sections were deparaffinized in xylene and rehydrated in a graded alcohol series (100%, 95%, 80% and 70% ethanol). Antigen retrieval was performed after heating in citrate buffer at 98 °C for 15 min. The sections were blocked with 3% H_2_O_2_ for 10 min at room temperature. After being blocked with TBST containing 5% goat serum at room temperature for 1 h, the sections were incubated with antibody against SETD6 (1:500; catalog No. ab220612; Abcam) at 4 °C overnight and with horseradish peroxidase universal immunoglobulin G secondary antibody (cat. No. sc69786; 1:1,000; Santa Cruz Biotechnology, Inc.) for 30 min at 37 °C. The signal was detected with 3,3′-diaminobenzidine solution using a light microscope (IX71; Olympus Corporation). For SETD6 protein, all visual fields were analyzed, the entire tissue specimen irrespective of its size was assessed and counted on the percentage of positive cells and the intensity of staining by Automatic Inverted Fluorescence Microscope.

### Cell line and cell culture

The A549 cell line was obtained from The American Type Culture Collection (ATCC, Manassas, VA, USA). A549 cells were cultured in DMEM with 10% fetal bovine serum (FBS; Gibco; Thermo Fisher Scientific, Inc.) at 37˚C in a humidified atmosphere with 5% CO_2_ until they reached 80%—90% confluence.

### Transfection, and retro- and lentiviral transduction

Retro- and lentiviral transfections were performed for knockdown experiments. Lentivirus for control, SETD6 shRNAs were purchased from NanJing PPL Biotechnology co. LTD. Three short hairpin RNA template oligonucleotides (SETD6-shRNA a, SETD6-shRNA b, and SETD6-shRNA c) based on three different parts of the human SETD6 gene (GenBank accession no. NM_001160305) were designed by NanJing PPL Biotechnology co. LTD. The sequences for the SETD6 shRNAs used in A549 cells were: 5’-CCT GTT CCC TGA AGG AAC AGC AAT -3’ for SETD6-shRNA a,5’-GCA GAC ATA CTA AAC CAC TTA-3’ for SETD6-shRNA b and 5’-GCG AAT TGT CTT CGG ATG GTA-3’ for SETD6-shRNA c. shRNAs directed against SETD6 were cloned into the shRNA vector pPLK GFP + Puroand the generated construct was confirmed by sequencing subsequently. SEDT6 shRNA or negative control (empty pPLK GFP + Puro vectors) were transfected into A549 cells using Lipofectamine 3000 reagent (Invitrogen, USA) according to the manufacturer’s protocol. Twenty-four hours after transfection, the protein expression of SEDT6 was detected by western blot.

For overexpression experiments, to obtain coding region of human wild-type SEDT6 cDNA, oligonucleotide primers complementary to the 5’and 3’ends of SEDT6 were designed by NanJing PPL Biotechnology co. LTD that incorporated the restriction sites EcoRI and Not I: Primer S, 5’-CCGGAATTCAATGGCATCCAAAAGAGC; and Primer A, 5’-CGGGGTACCCTAGTCTTTGAGAACAAGCG. Polymerase chain reaction (PCR) products were digested with EcoRI and Not I and then analyzed by agarose gel electrophoresis. Bands of the expected length were cut out and ligated into the lentiviral expression vector plenti–CMV-GFP-Plur (Sigma, St. Louis, MO.,USA), which had been digested with EcoRI and Not I. Subsequently, the generated construct was confirmed by sequencing and further referred to as SEDT6-3*Flag. SEDT6-3*Flag or empty plenti–CMV-GFP-Plur vectors of negative control were transfected into A549 cells using Lipofectamine 3000 reagent (Invitrogen, USA) following the manufacturer’s protocol. Twenty-four hours after transfection, the protein expression of SEDT6 was detected by western blot.

### MTT for cell viability

The 3-(4,5-dimethylthiazol-2-yl)-2,5-diphenyl tetrazolium bromide (MTT) cell proliferation and cytotoxicity assay kit (Nanjing Jiancheng Bioengineering Institute) was used to monitor the status of cell proliferation. Briefly, A549 cells were cultured in DMEM supplemented with 10% FBS at 37℃ under atmosphere of 5% CO_2_, and subcultured by 0.25% trypsin every 2 or 3 days. The cells were inoculated into 96-well plates with 1 × 10^4^ cells per well, and the volume of each well was 100 μl. After incubation for 24 h, the cells were incubated at 37℃ and 5% CO_2_. Add 50ul of 1 × MTT solution to each well, continue to incubate for 4 h, then stop culture, and carefully absorb and discard the culture supernatant. Add 150 µl DMSO to each well and shake for 10 min to dissolve the crystals. The optical density (OD) value was determined by an automatic microplate reader at 570 nm wavelength. The cell viability rate was calculated according to the following formula: Cell viability (%) = (OD value of compound group/OD value of the control group) × 100%.

### Caspase-3 activity assay

Caspase-3 activity was analyzed using the Caspase3 Colorimetric Assay Kit (Beyotime Biotechnology, Shanghai, China) following the manufacturer’s guide. Briefly, A549 cells were cultured in 6-well plate and allowed to attach overnight. After SETD6-shRNA transfection, cells were washed with PBS and lysed. The cell lysates were centrifuged at 13,000 g for 15 min. The blank solution, containing 90 μl reaction buffer and 10 μl Ac-DEVD-pNA, and the sample solution, containing 75 μl reaction buffer, 15 μl sample, and 10 μl Ac-DEVD-pNA, were incubated in a 96-well microplate overnight at 37 °C. Absorbance was measured at 405 nm in a microplate reader.

### Cell migration assays

Cell migration was assessed by wound healing cell scratch migration assays. A549 cells were seeded into 6-well plate with a cell density of 6 × 10^5^cells/ml. At 80–90% confluent, the cell monolayer was scraped with a sterile 200 μl pipette tip, followed by PBS washing for three times. After incubation for 0 h, 24 h, and 48 h, the cells were fixed, then observed and images were taken under inverted fluorescence microscope. The migration distance and mobility were calculated using Python-based image analysis software named Python(x, y) (v1.0, Shanghai Genechem Co., Ltd.).

### Colony-formation assay

The cloning ability of A549 cells was detected using a colony formation assay. Briefly, cells in each treatment group were cultured in 6-well culture plates for 2 weeks to form colonies. Colonies were stained with crystal violet (2%) and counted under inverted microscope (Olympus, Tokyo, Japan). Colony numbers were calculated from five independent fields. The experiments were repeated for three times.

### Apoptosis analysis

Apoptotic cells were detected by using the Hoechst 33,258 staining (Beyotime, Haimen, China). A549 were seeded with a density of 6 × 10^5^ cells/well in 6-well plate. The cells were fixed with 4% paraformaldehyde overnight at 4℃, washed with PBS for three times and then stained with 2 μg/ml Hoechst 33,258 for 5 min. Morphologic changes in apoptotic nuclei were evaluated under a fluorescence microscope (excitation wavelength 350 nm, emission filter 460 nm) (Olympus Fluoview, Japan). Five vision fields of each well were selected in parallel to take photos and calculate the apoptosis rate. Apoptosis rate (%) = (cell count of apoptosis cell/ total cell count) × 100%.

### Protein extraction and immunoblotting

A549 cells were seeded into 6-well plate with a cell density of 1 × 10^5^cells/ml. After related pretreatment, incubation was terminated and A549 cells in logarithmic growth phase were harvested. After aspirating the cell culture medium, the cells were washed with PBS solution for 2–3 times. Cytoplasmic and nuclear proteins were obtained from the cells using protein extraction kits (Beyotime Institute of Biotechnology, Shanghai, China). The protein content was determined by BCA Protein Assay Kit (Beyotime, Haimen, Jiangsu, China). Equal amounts of protein samples (15 μg) were separated at 10% of sodium dodecyl sulphate–polyacrylamide gel electrophoresis (SDS-PAGE) and then transferred to 0.22 μm polyvinylidene difluoride (PVDF) membrane (Bio-Rad, USA). Non-specific binding was blocked with 5% non-fat dry milk in 0.1% Tween-20/TBS (TBST) at room temperature for 1 h. After washing with TBST, the membranes were cut into several smaller pieces according to protein ladders (molecular weight markers), and each pre-cut PVDF membrane was incubated with its corresponding primary antibody (SETD6, NF-κB p65, Nrf2, Keap1, HO-1, or SOD-1, all purchased from Abcam (Cambridge, MA, USA)) overnight at 4 °C. On the next day, the membrane was washed, and second antibody (1:2000) coupled with horseradish peroxidase secondary antibodies (Abcam, Cambridge, MA, USA) was incubated at room temperature for 1 h, followed by enhanced Chemiluminescence reagent BeyoECL (Beyotime, Shanghai, China) detection. Membranes were stripped and re-probed for β-actin or GAPDH to demonstrate equal loading. The values of band intensities were quantified by Quantity One 4.6.2 software (Bio-Rad Laboratories, Hercules, CA, USA) to the respective protein loading controls. All immunoblots shown here are representatives of at least three independent experiments.

### Statistical analysis

All values are presented as the means ± standard deviation (SD) from at least three individual experiments. Statistical analysis of the differences between the groups were performed with an unpaired Student’s t-test by using GraphPad 5.0 software. Fisher’s exact test was used to analyze the relationship between SETD6 expression and clinicopathological characteristics. Survival curve was generated by the Kaplan–Meier method, and the log-rank test was used for assessing the statistical significance between the groups. *P* < 0.05 was considered to indicate a statistically significant difference.

## Results

### Up-regulation of SETD6 in LUAD tissues

**Fig. 1:**
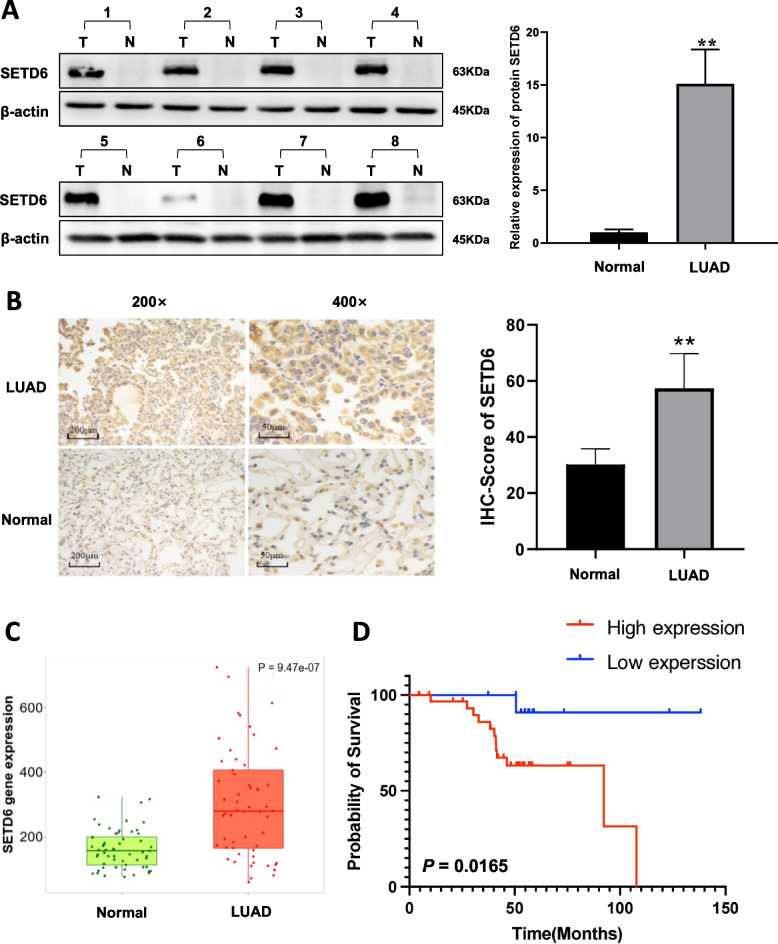
SETD6 was up-regulated in tumor tissues and was associated with a substantially poorer prognosis in LUAD patients. **A** Representative western blot images and comparative analysis of SETD6 expression in LUAD tumor tissues (named T) and adjacent normal tissuses (named N). Protein bands were quantified by Quantity One 4.6.2 software analysis. **p < 0.01. **B** Typical IHC staining and comparative IHC score of SETD6 in LUAD tumor tissues and adjacent normal tissues. **p < 0.01. **C** Data of *SETD6* mRNA expression in tumor-adjacent normal tissue and malignant LUAD tissue was taken from the TMNplot.com Analysis Platform. **D** Kaplan–Meier overall survival curves of LUAD patients with high (*n* = 32) or low (*n* = 13) expressions of SETD6 in tumor tissues as determined by IHC staining

To identify whether SETD6 plays an important role in LUAD, we evaluated SETD6 expression in 45 paired LUAD and adjacent non-tumorous tissues. We found that SETD6 showed significantly higher protein levels in tumor samples compared with adjacent normal tissues both by western blotting or IHC analysis (Fig. 1A, B). In addition, TNMplot (https://tnmplot.com/analysis/) [12], a web tool for differential gene expression analysis in normal and tumors tissues, was used to validate *SETD6* differential expression pattern. Consistant with our findings, *SETD6* gene expression was significantly higher in LUAD *vs.* normal tissues (*p* = 9.47 × 10^−7^) (Fig. 1C).

### Association between the expression of SETD6 and the clinicopathological features in LUAD

To determine whether the expression level of SETD6 impacts the prognosis of patients with LUAD, we analyzed IHC data of SETD6 in the 45 cases with recorded clinical follow-up data. The Kaplan–Meier analysis revealed that LUAD patients with higher SETD6 expression exhibited significantly poorer survival (*p* = 0.0165) (Fig. 1D). We additionaly analyzed the correlation between SETD6 expression levels and other clinicopathological parameters (Table 1). We found that SETD6 expression was not significantly correlated with gender, smoking index, clinical stage and tumor size. However, higher SETD6 expression was significantly associated with higher rates of regional lymph node metastasis (*p* = 0.0083). These data suggest that SETD6 may exert potential oncogenic function during LUAD metastasis and development.

**Table 1:** Association between SETD6 expression and clinicopathologic features

Variables	Number of patients	SETD6 expression (%)		*P*
		Low/none	High	
Total	45	13 (28.9)	32 (71.1)	
**Gender**				
Male	29	10 (34.5)	19 (65.5)	0.3223
Female	16	3 (18.8)	13 (81.2)	
**Age**				
< 60	13	8 (61.5)	5 (38.5)	0.0039**
≥ 60	32	5 (15.6)	27 (84.4)	
**Smoking index**				
> 400	17	7 (41.2)	10 (58.8)	0.1884
< 400	28	6 (21.4)	22 (78.6)	
**TNM stage**				
I-II	25	9 (36.0)	16 (64.0)	0.3271
III-IV	20	4 (20.0)	16 (80.0)	
**Regional lymph node metastasis**				
Yes	28	4 (14.3)	24 (85.7)	0.0083**
No	17	9 (52.9)	8 (47.1)	
**Tumor size**				
≥ 3 cm	26	5 (19.2)	21 (80.8)	0.1114
< 3 cm	19	8 (42.1)	11 (57.9)	

^**^p < 0.01

### The functional role of SETD6 in regulating malignant behaviors of LUAD cells

In order to uncover the role of SETD6 in regulating malignant behaviors including cell proliferation, migration and colony formation of LUAD cells, we preformed gain-of-function and loss-of-function experiments in LUAD cell line A549 with SETD6 overexpression and knockdown strategies.

**Fig. 2:**
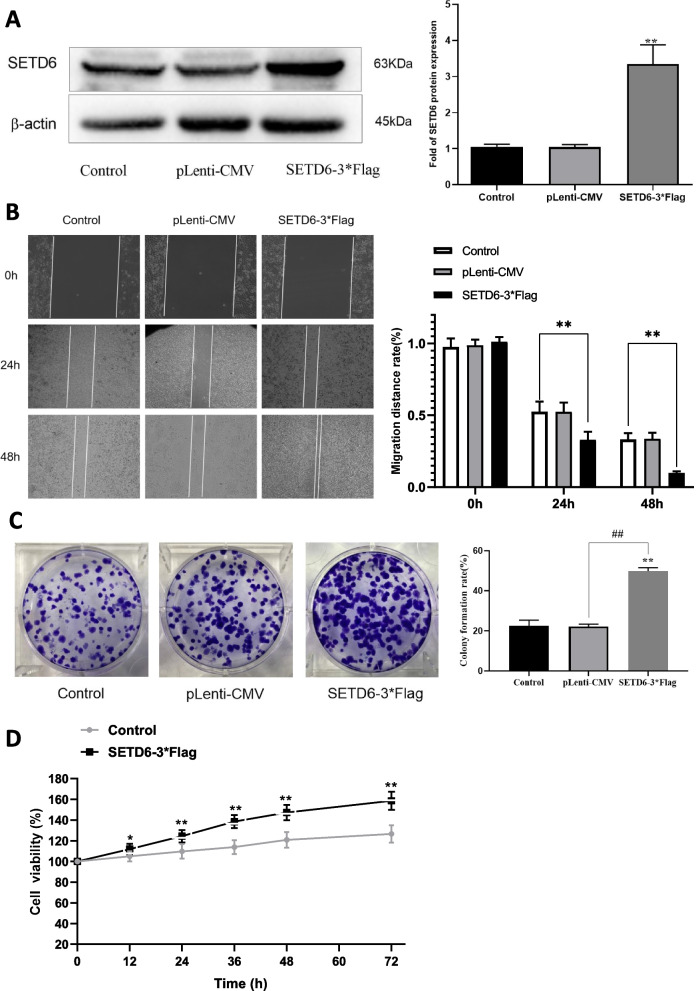
The effect of SEDT6 overexpression with SEDT6-3*Flag transfection on A549 cell migration, colony formation and viability. **A** SETD6 protein levels were determined by western blot in control, plenti-CMV or SEDT6-3*Flag transfected A549 cells. β-actin served as loading control. Protein bands were quantified by Quantity One 4.6.2 software analysis. **B** Cell migration rate of control, plenti-CMV or SEDT6-3*Flag transfected A549 cells at indicated time points (0 h, 24 h, 48 h) were determined by wound healing assay. **C** Colony formation rate of control, plenti-CMV or SEDT6-3*Flag transfected A549 cells were determined by colony formation assay. **D** Cell viability of control, plenti-CMV or SEDT6-3*Flag transfected A549 cells were determined by MTT method. The cell viability was measured at different time (12, 24, 36, 48, 60, 72 h) and the percentage of survival of cells relative to controls was calculated. *, *P* < 0.05 compared to control group; **, *P* < 0.01 compared to control group; ^##^, *P* < 0.01 compared to plenti-CMV group

For overexpression purpose, SEDT6-3*Flag or empty plenti-CMV-GFP-Plur vectors were transiently transfected into A549 cells. The transfection of SEDT6-3*Flag leaded to successful overexpression of SETD6 in A549 cells (Fig. 2A). Accompanied with elevated SETD6 expression, the cell migration (Fig. 2B), colony formation ability (Fig. 2C) and cell proliferation (Fig. 2D) were all increased. Taken together, these data demonstrated that increased expression of SETD6 plays an important role in promoting malignant behaviors of LUAD cells.

**Fig. 3:**
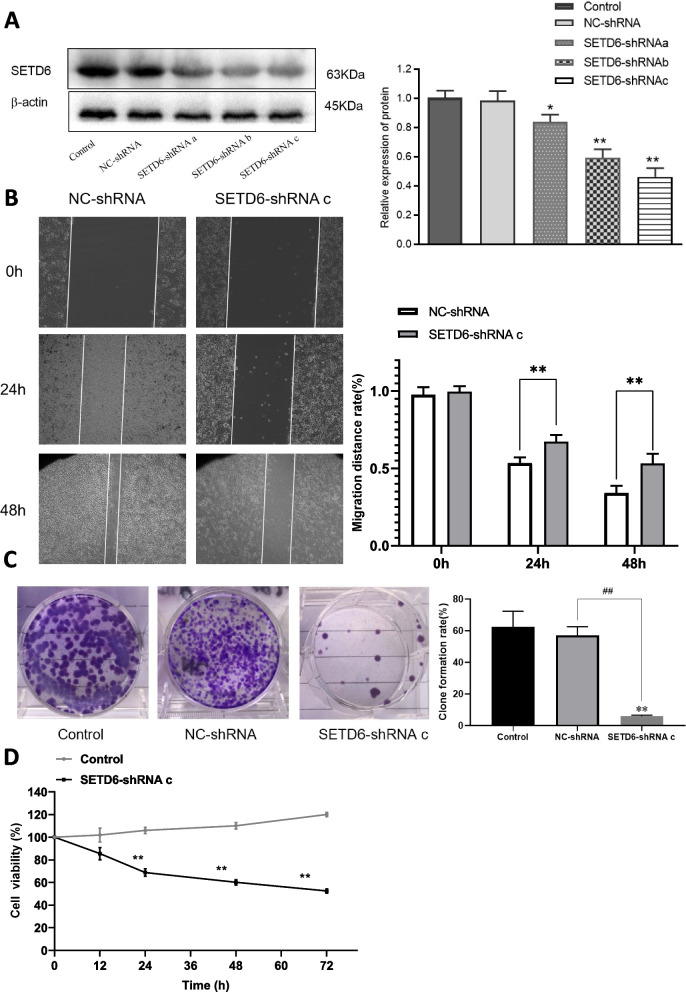
The effect of SEDT6 knockdown with SETD6-shRNA transfection on A549 cell migration, colony formation and viability. **A** SETD6 protein levels were determined by western blot in control, NC-shRNA or SEDT6-shRNA a, or b, or c transfected A549 cells. β-actin served as loading control. Protein bands were quantified by Quantity One 4.6.2 software analysis. **B** Cell migration rate of NC-shRNA or SEDT6-shRNA c transfected A549 cells at indicated time points (0 h, 24 h, 48 h) were determined by wound healing assay. **C** Colony formation rate of control, NC-shRNA or SEDT6-shRNA c transfected A549 cells were determined by colony formation assay. **D** Cell viability of control or SEDT6-shRNA c transfected A549 cells were determined by MTT method. The cell viability was measured at different time (12, 24, 36, 48, 60, 72 h) and the percentage of survival of cells relative to controls was calculated. *, *P* < 0.05 compared to control group; **, *P* < 0.01 compared to control group; ^##^, *P* < 0.01 compared to NC-shRNA group

For knockdown purpose, three lentiviral vector based shRNA (SETD6-shRNA a, SETD6-shRNA b, and SETD6-shRNA c) designed against *SETD6* mRNA sequence were transfected in A549 cells to down-regulate the expression of SETD6. It showed that SETD6-shRNA c exerted the most inhibitory effect on the expression of SETD6 compared to other groups (Fig. 3A). Thus, we chose SETD6-shRNA c for loss-of-function assay in the following experiment. Transfection with SETD6-shRNA c resulted in a considerable decreased cell migration (Fig. 3B), colony formation ability (Fig. 3C) and cell proliferation (Fig. 3D), suggesting that silencing of SETD6 can inhibit the malignant behaviors of LUAD cells.

### The effect of SETD6 on apoptosis of LUAD cells

**Fig. 4:**
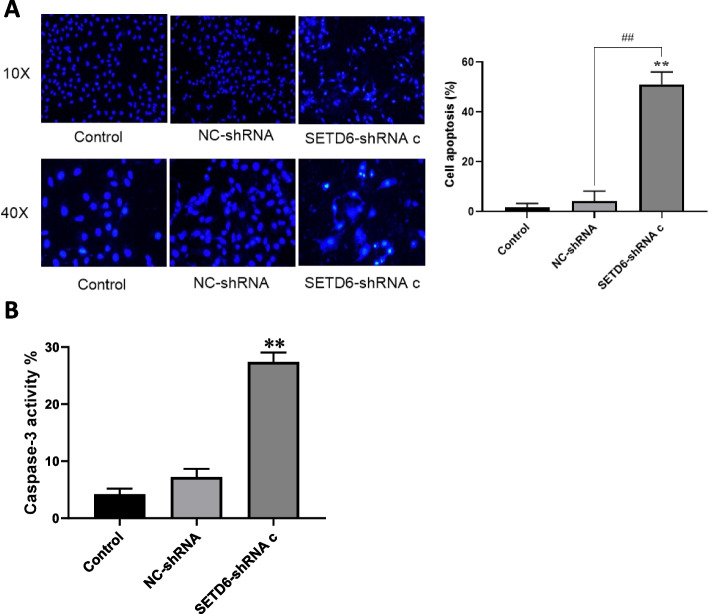
The effect of SETD6 knockdown on apoptosis of A549 cells. **A** Phenomenon of cell apoptosis with Hoechst stains was observed by fluorescent microscop under the ten times scope and forty times scope. Cell apoptosis rates were analyzed in control, NC-shRNA or SETD6-shRNA c transfected A549 cells. **B** Caspase-3 activities of control, NC-shRNA or SETD6-shRNA c transfected A549 cells were determined by pNA concentrations. **, *P* < 0.01 compared to control group; ^##^, *P* < 0.01 compared to NC-shRNA group

The function of SETD6 in regulating cell apoptosis was evaluated by transfecting SETD6-shRNA c into A549 cells, cell apoptosis and caspase-3 activity were detected accordingly. The results showed that silencing of SETD6 with SEDT6-shRNA c caused increased apoptosis of A549 cells (Fig. 4A), with significantly enhanced caspase-3 activity (Fig. 4B).

### The regulatory effect of SETD6 on NF-κB and Nrf2 signaling pathways

Given that activation of NF-κB and Nrf2 signaling pathways were reported as critical signals that can suppress cell apoptosis and promote malignant behaviors such as cell proliferation in transformed cells, we further investigated if NF-κB and Nrf2 were regulated by SETD6 in LUAD cells.

**Fig. 5:**
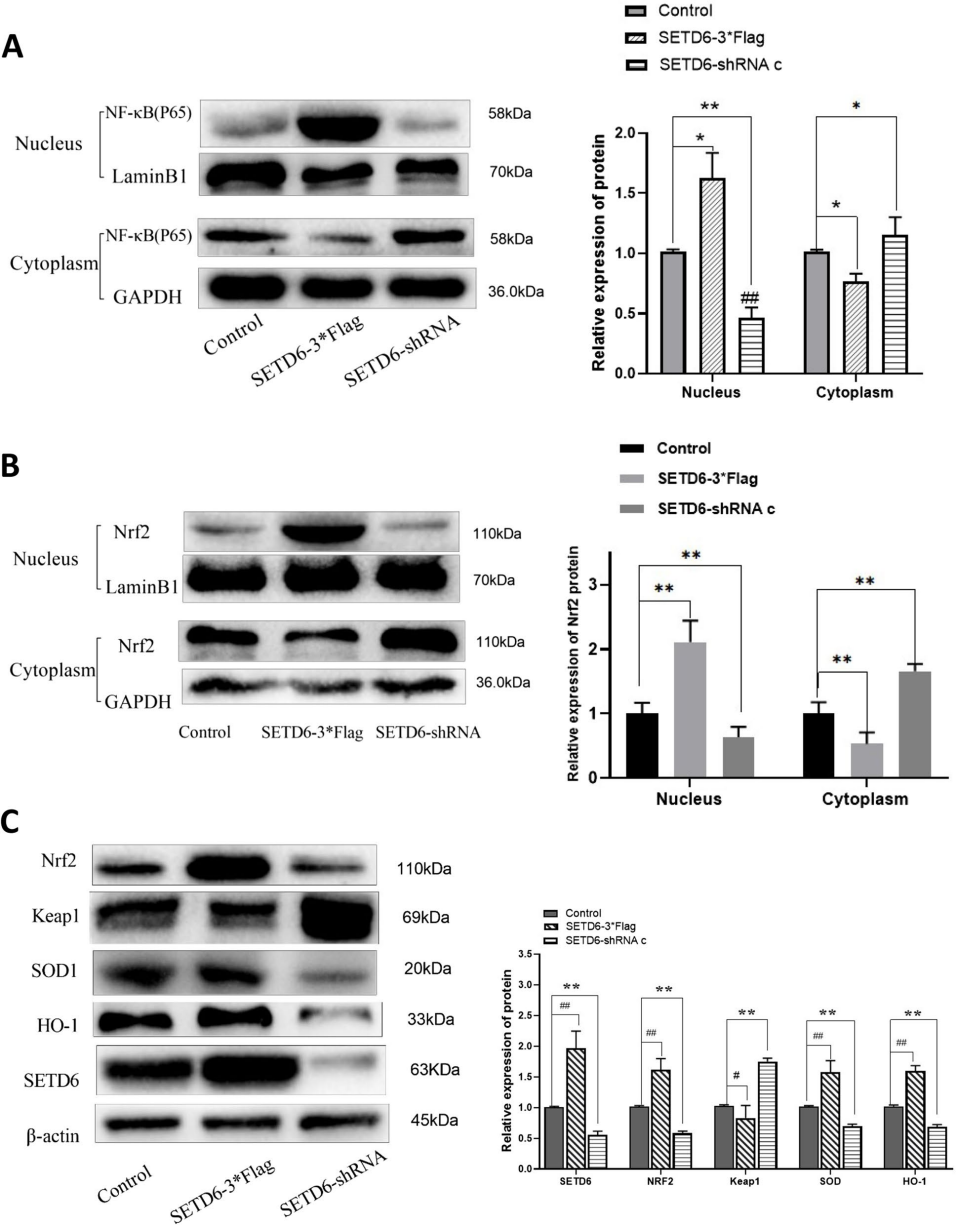
The regulatory effect of SETD6 on NF-κB and Nrf2 signaling pathways. The protein levels of (**A**) intra or extranuclear P65, and (**B**) intra or extranuclear Nrf2, in control, SEDT6-3*Flag or SETD6-shRNA c transfected A549 cells were analyzed by Western blot. LaminB1 served as loading control for nuclear protein and GAPDH served as loading control for cytoplasm protein. **C** The protein levels of SETD6, Nrf2, Keap1, HO-1, SOD-1 were measured by Western blot. β-actin served as loading control. Protein bands were quantified by Quantity One 4.6.2 software analysis. *, *P* < 0.05 compared to control group; **, *P* < 0.01 compared to control group; ^#^, *P* < 0.05 compared to SEDT6-3*Flag group; ^##^, *P* < 0.01 compared to SEDT6-3*Flag group

By transfecting A549 cells with SEDT6-3*Flag, we found increased NF-κB (P65) and Nrf2 level in nucleus but decreased NF-κB (P65) and Nrf2 level in cytoplasm (Fig. 5A, B), suggesting that NF-κB (P65) and Nrf2 translocated from cytoplasm to nucleus, which is a sign of NF-κB and Nrf2 signal activation. In addition, Keap1, a repressor protein that binds to Nrf2 and promotes its degradation, was downregulated after SEDT6-3*Flag transfection, and downstream targets of Nrf2 signaling like SOD1, HO-1 were all increased upon SEDT6 overexpression (Fig. 5C). In contrast, knockdown of SEDT6 with SETD6-shRNA c caused the opposite effect (Fig. 5A, B, C). Taking together, these data indicated that NF-κB and Nrf2 signalings are regulated by SEDT6 in A549 cells and may be involved in SEDT6-mediated cell apoptosis and proliferation processes.

## Discussion

The occurrence and development of tumor involve not only genetic changes, but also epigenetic changes [13]. PKMTs are critical for epigenetic regulation of gene expression by adding methyl group(s) to lysine residues of histones and non-histone proteins such as transcription factors, thereby participating in a variety of biological and pathological process [14]. Over the past decades, inhibitors that target PKMTs have been shown promising anti-tumor effects, and have been developed as precision cancer therapeutic tools [15]. For instance, enhancer of zeste homolog 2 (EZH2), which is a well-known histone-lysine N-methyltransferase enzyme, was found highly expressed in a variety of cancers, and a selective inhibitor such as tazemetostat that block EZH2 activity was shown to exert profound anti-tumor effects [16, 17]. Other PKMTs like JMJD2C, SMYD2, have also been reported in human cancers such as colorectal cancer, breast cancer [18, 19], but information on PKMTs are generally lacking in LUAD. Here in our study, we identified SETD6, which is a novel PKMT, to be highly upregulated in LUAD tissues. This is in accordance with previous findings in other cancer types, showing that overexpression of SETD6 was present in diffuse gastric adenocarcinoma, adenocarcinoma of the colon, rectum, cecum, and rectal mucinous adenocarcinoma compared to their respective normal tissues [20]. Importantly, higher SETD6 expression was significantly associated with poorer overall survival and higher rates of regional lymph node metastasis in our study, indicating that SETD6 correlated with aggressive tumor behavior in LUAD patients.

As mentioned in earlier studies, SETD6 acts as an important regulator of the cell proliferation, apoptosis, invasion and metastasis [3–10]. In the present study, we showed that, in LUAD cells, SETD6 knockdown inhibited cell proliferative ability in vitro, and led to enhanced apoptosis, while an increase of SETD6 promoted cell proliferative ability. Consistent with its effects on proliferation, SETD6 expression level was also correlated with the colony formation and migrative potential of LUAD cells. Further in vivo studies using cell lines or patient-derived xenograft (PDX) models are required to elucidate the role of SETD6 in promoting malignant behaviors of LUAD.

Previous studies in multiple cancer types have attempted to elucidate the potential mechanisms of SETD6-mediated cancer development. NF-κB is a critical signaling that plays an important role in inflammation and innate immunity, and also participates in cancer development by inhibiting tumor cell apoptosis [21, 22]. In bladder cancer, SETD6 was reported to activate NF-κB signaling and promote urothelial cell survival [9]. In accordance with the aforementioned studies, the results of present study also demonstrated that SETD6 overexpression activated NF-κB signaling in LUAD cells, while SETD6 down-regulation suppressed NF-κB signaling and promoted Caspase-3 activity and apoptosis in LUAD cells. Moreover, Nrf2/Keap1 signaling, which is a key signaling pathway that can enhance anti-oxidative stress capacity of cancer cells and exert anti-apoptotic effects [23, 24], was also regulated by SETD6 in our study. Compared with the SETD6 overexpression group, the down-regulation of SETD6 increased the expression of Keap1, which is a suppressor of Nrf2, and significantly inhibited the Nrf2 protein level in nucleus. Furthermore, Nrf2 downstream targets like SOD and HO-1 were also suppressed upon SETD6 knockdown. Taking together, these data indicated that SETD6 may participate in the process of apoptosis and oxidative stress of LUAD cells through NF-κB and Keap1/Nrf2 pathway, thus affecting the development of tumor. In this regard, further screening and identification of selective inhibitors of SETD6 may exert anti-cancer effects in LUAD. However, further investigations will be required in animal models targeting SETD6 to substantiate its therapeutic capability.

There are some advantages and limitations of our study. This is a novel study which for the first time evaluated the clinical relevance of SETD6 in patients with LUAD, and uncovered intrinsic mechanisms which are involved in SETD6-mediated malignant behaviors of LUAD, paving the way for novel therapeutic target development. However, our research also suffered from several limitations. First, this was a study based on limited sample size from one medical center, which may have resulted in bias. Further validation of our findings in a larger cohort of patients from multi-centers is critical to minimize the biases possibly deriving from the heterogeneity of LUAD. Second, our molecular studies were only carried out in one cell model. Although the A549 cell line is a widely used human LUAD cell line, comprehensive molecular investigations are warranted to be carried out in additional cells lines to further validate our findings in the foreseeable future.

## Conclusion

In summary, this study demonstrated that SETD6 is upregulated in LUAD tumor tissues, and its overexpression significantly correlates with higher rates of regional lymph node metastasis and poor prognosis in patients with LUAD, which indicated that SETD6 has a potential role in promoting LUAD development. Molecular studies showed that SETD6 overexpression could promote LUAD cell proliferation, migration, colony formation and inhibit cell apoptosis. Further mechanistic investigations revealed that NF-κB and Nrf2 signaling pathways may participate in SETD6-regulated malignant behaviors of LUAD cells. Our findings shed new light on mechanisms of LUAD development, and suggest that SETD6 could be a promising therapeutic target to consider for LUAD treatment.

## Supplementary Information

**Additional file 1.**

## Data Availability

The datasets used and/or analyzed during the current study are available from the corresponding author on reasonable request.

## Abbreviations

LUAD: Lung Adenocarcinoma
SETD6: SET domain containing 6
PKMTs: Protein lysine methyltransferases
IASLC: International Association for the study of lung cancer
NF-κB: Nuclear factor-κB
Nrf2: Nuclear factor erythroid 2–related factor 2
EZH2: Enhancer of zeste homolog 2
PDX: Patient-derived xenograft
