# Supplementary material for: Investigating the functional role of SETD6 in lung adenocarcinoma
Source: BMC Cancer. 2023 Jan 6;23:18. doi: 10.1186/s12885-022-10476-9 (PMC9817333; doi:10.1186/s12885-022-10476-9)
Supplement: Supplementary file 1 — Additional file 1. [file 12885_2022_10476_MOESM1_ESM.pptx]

## Slide 1
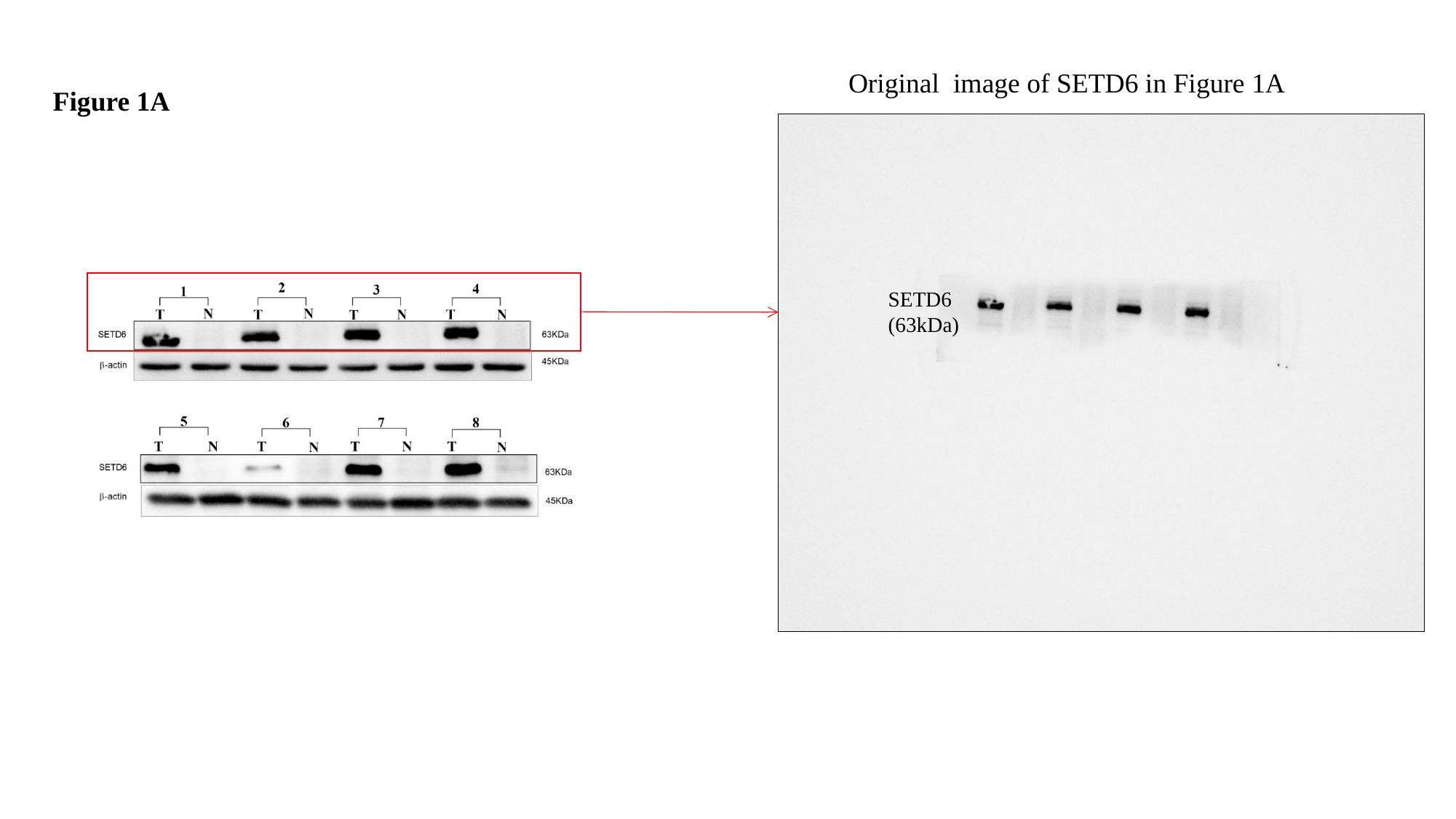

Original image of SETD6 in Figure 1A
Figure 1A
SETD6
(63kDa)

## Slide 2
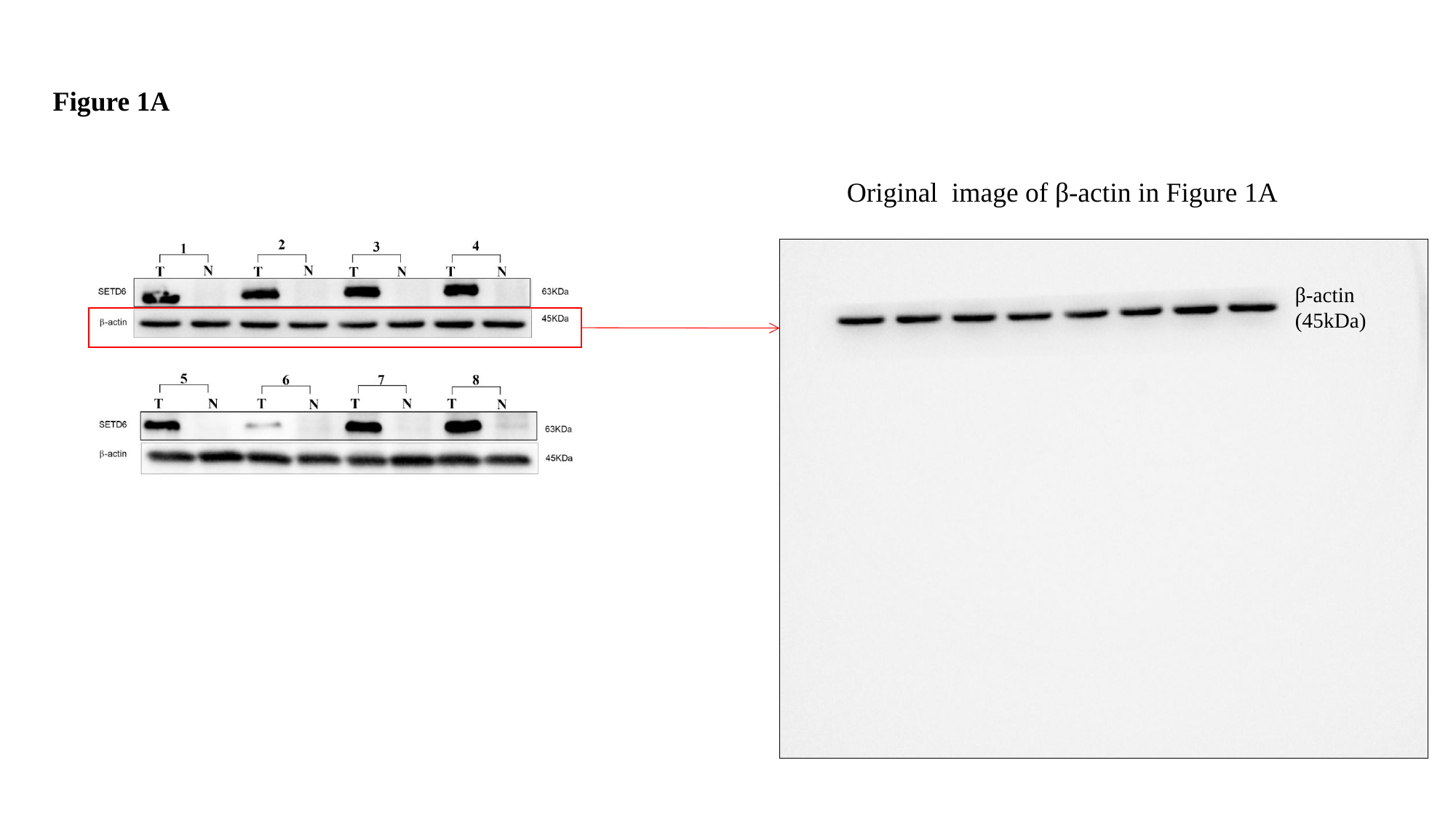

Figure 1A
Original image of β-actin in Figure 1A
β-actin (45kDa)

## Slide 3
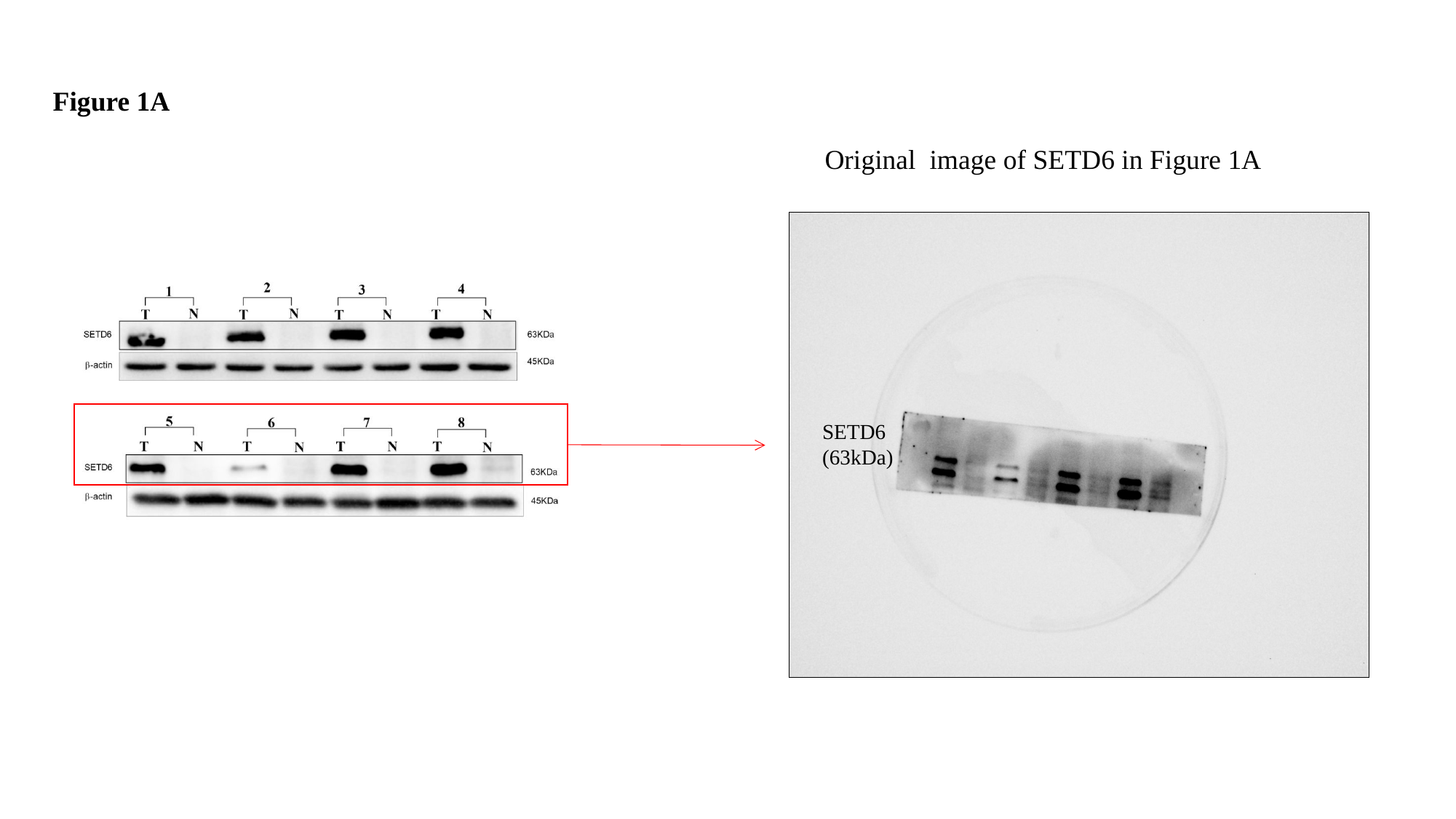

Figure 1A
Original image of SETD6 in Figure 1A
SETD6
(63kDa)

## Slide 4
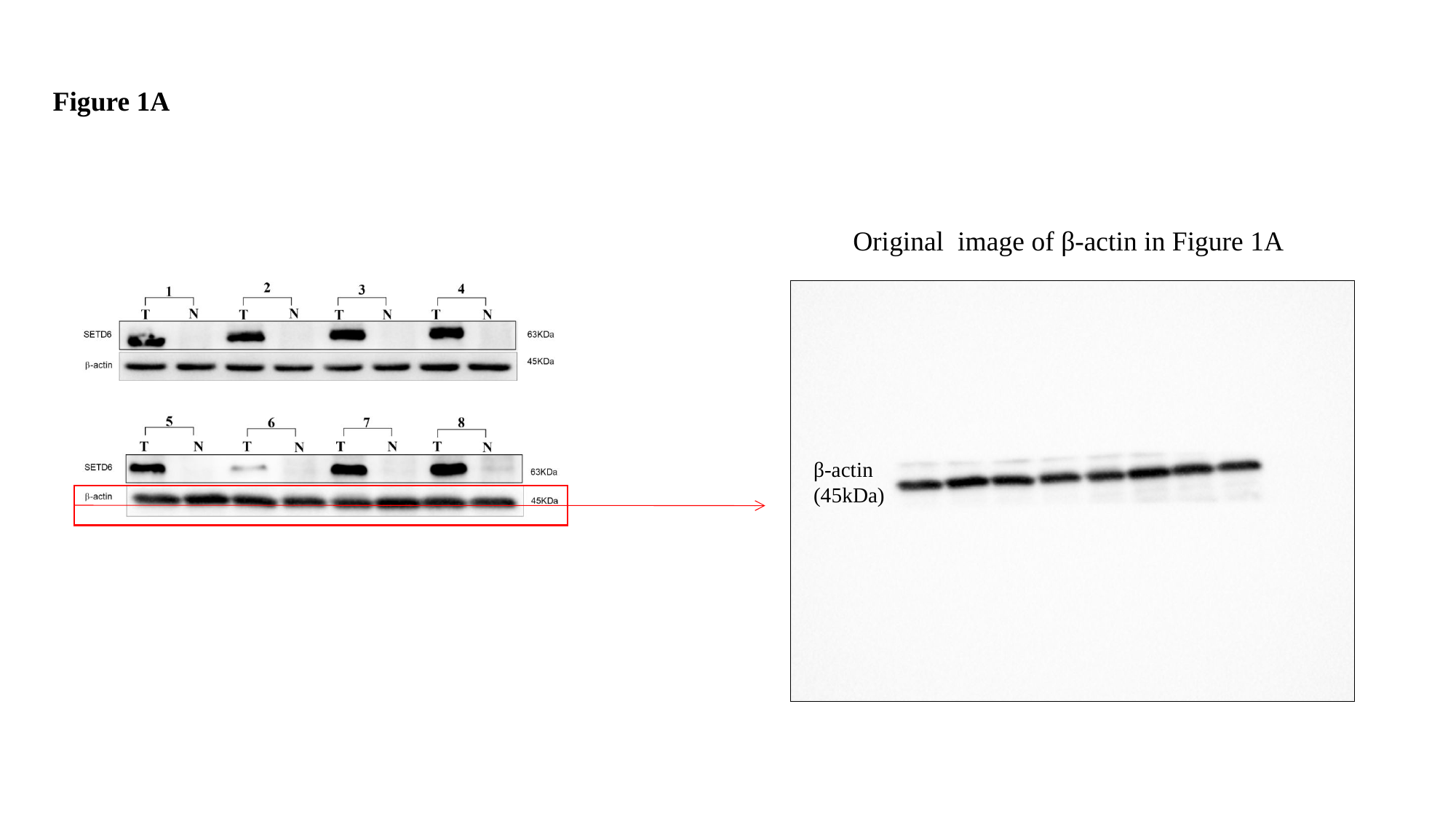

Figure 1A
Original image of β-actin in Figure 1A
β-actin (45kDa)

## Slide 5
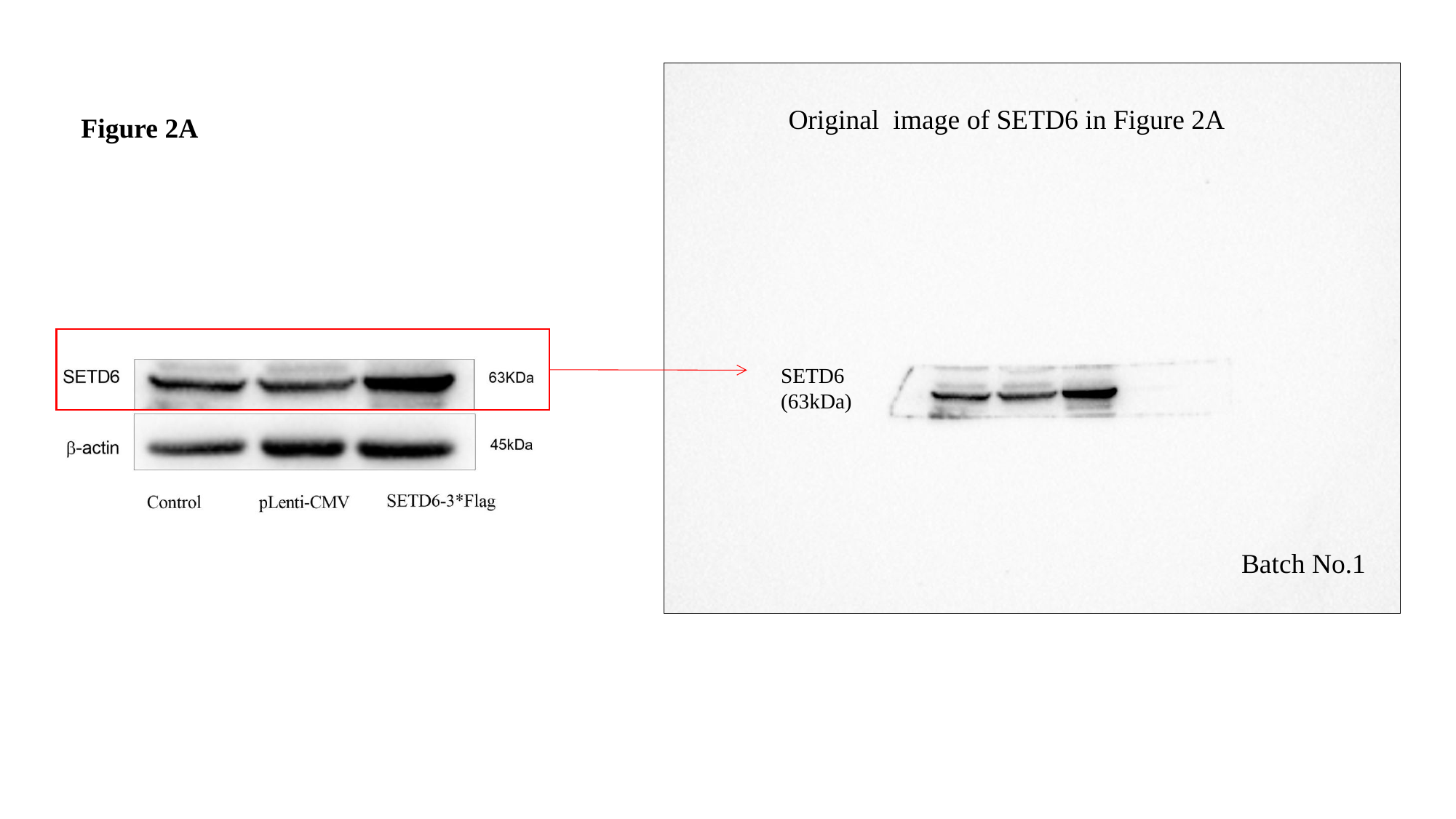

Original image of SETD6 in Figure 2A
Figure 2A
SETD6
(63kDa)
Batch No.1

## Slide 6
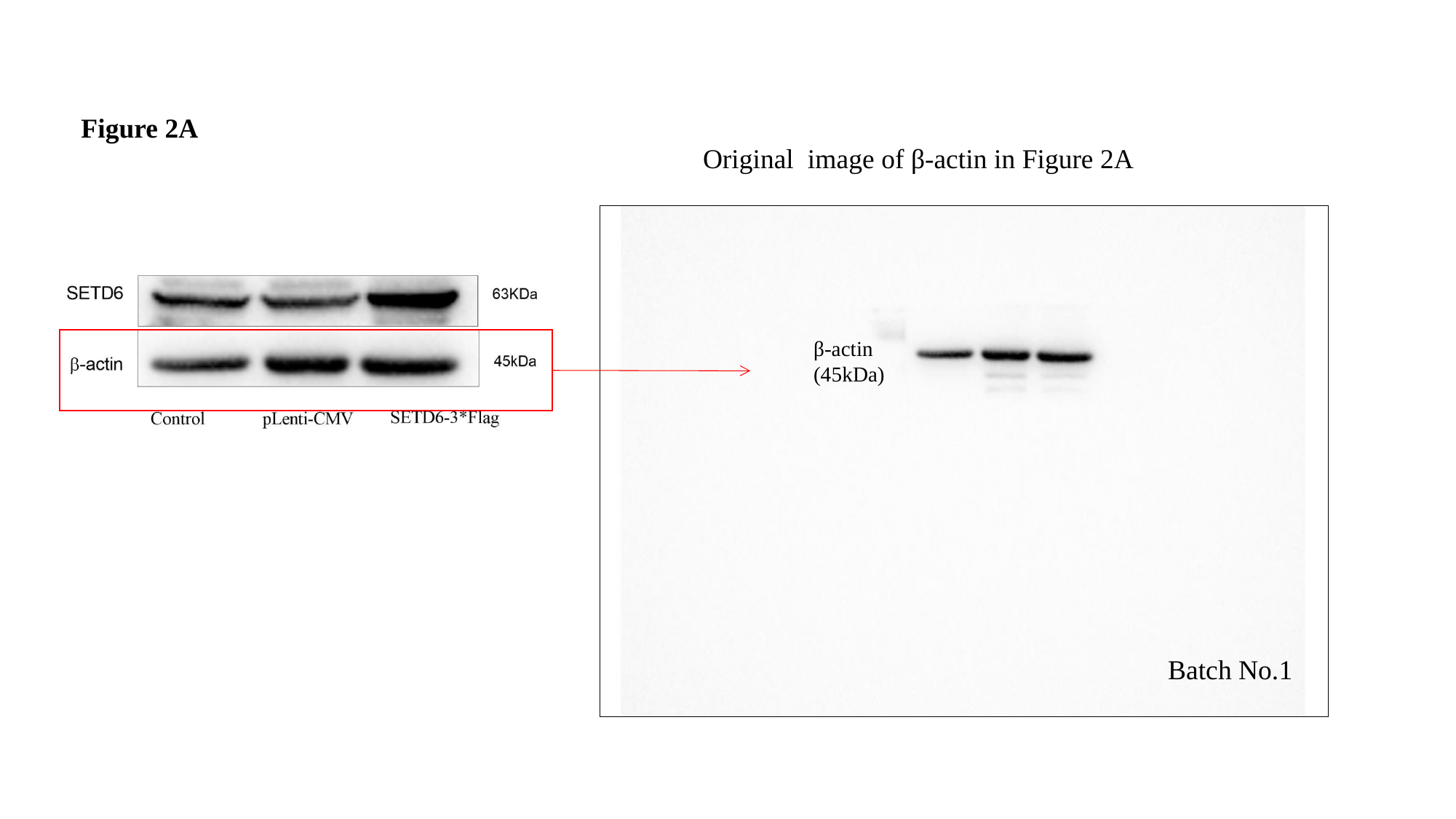

Figure 2A
Original image of β-actin in Figure 2A
β-actin (45kDa)
Batch No.1

## Slide 7
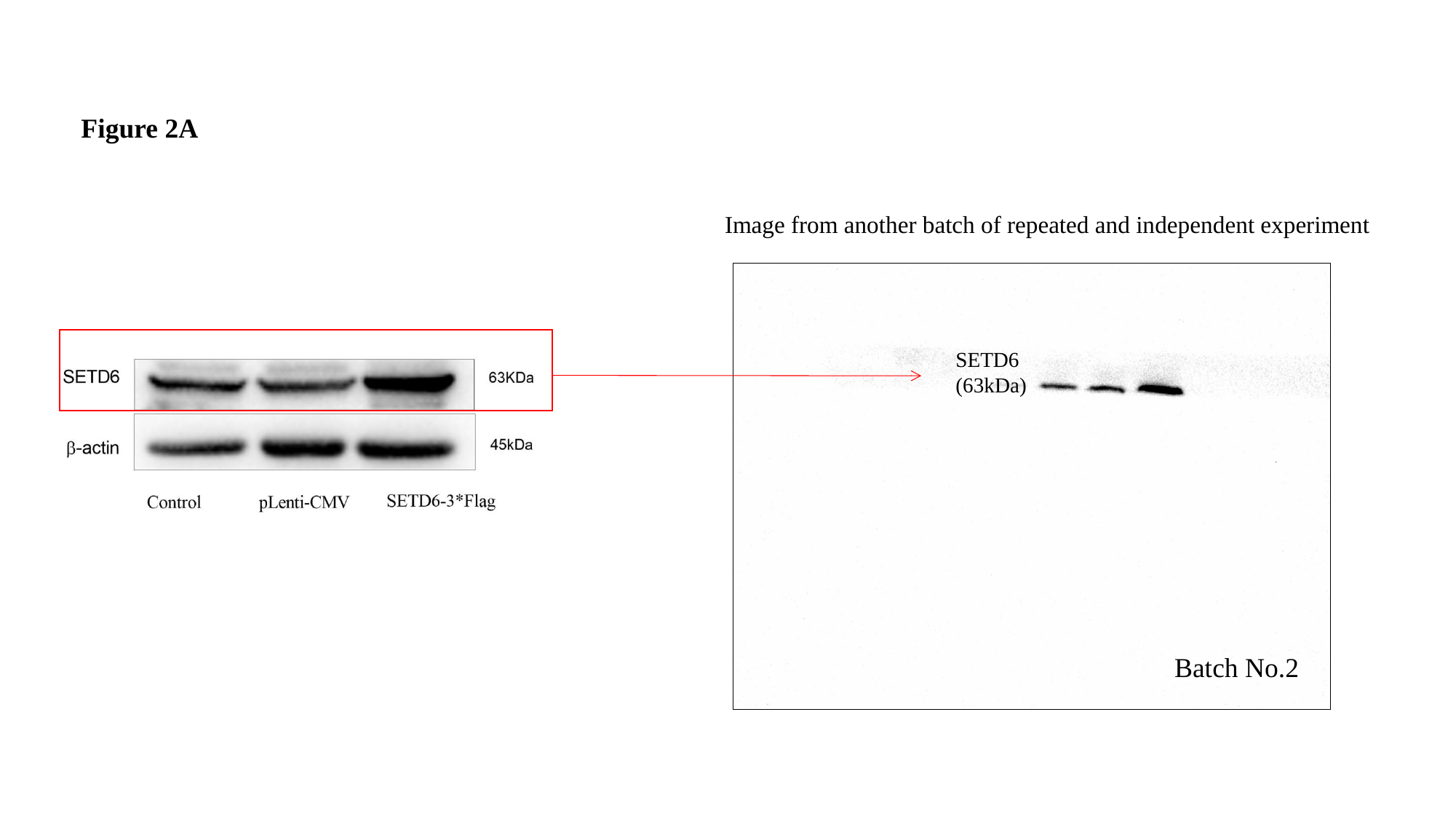

Figure 2A
Image from another batch of repeated and independent experiment
SETD6
(63kDa)
Batch No.2

## Slide 8
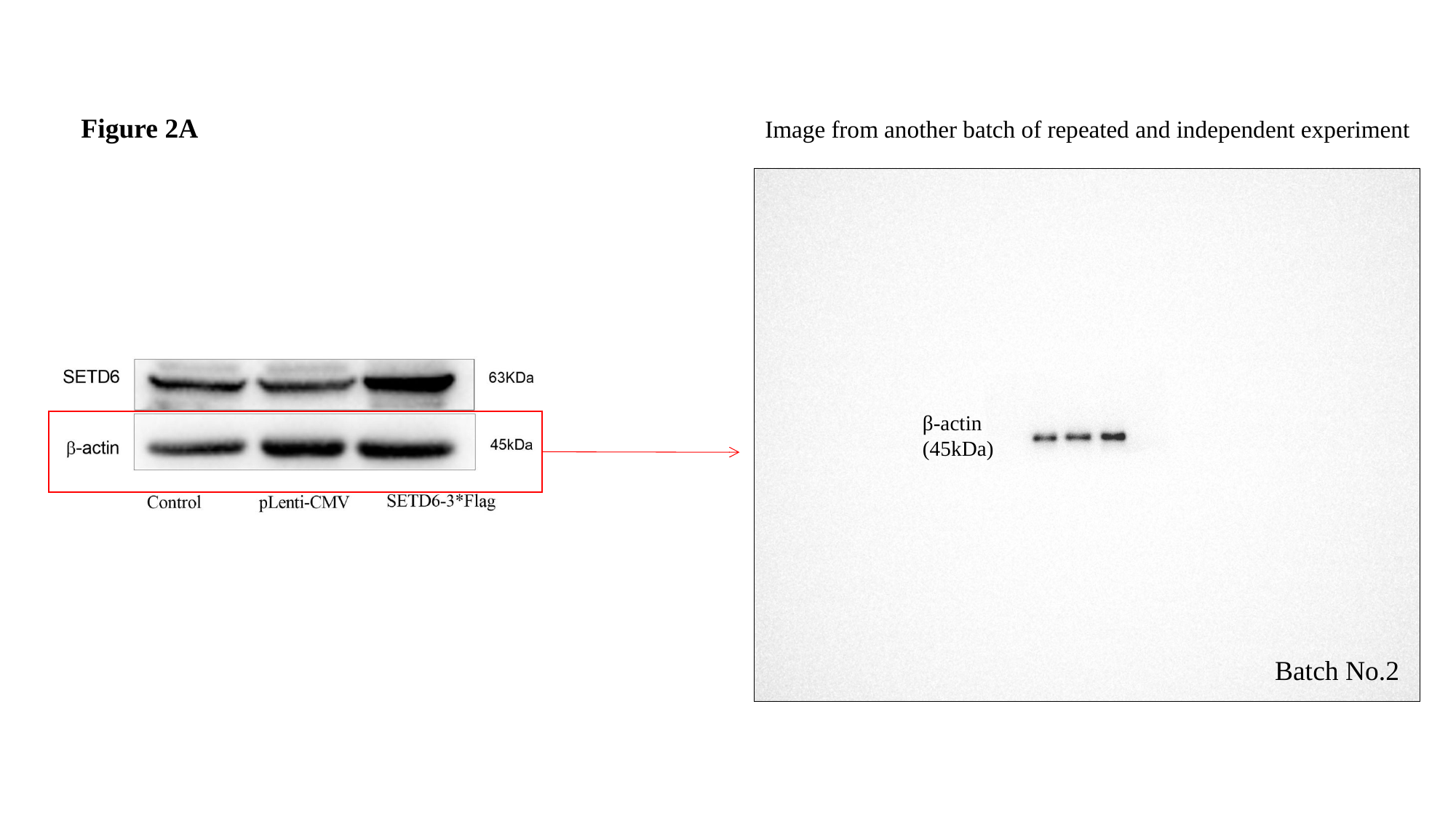

Figure 2A
Image from another batch of repeated and independent experiment
β-actin (45kDa)
Batch No.2

## Slide 9
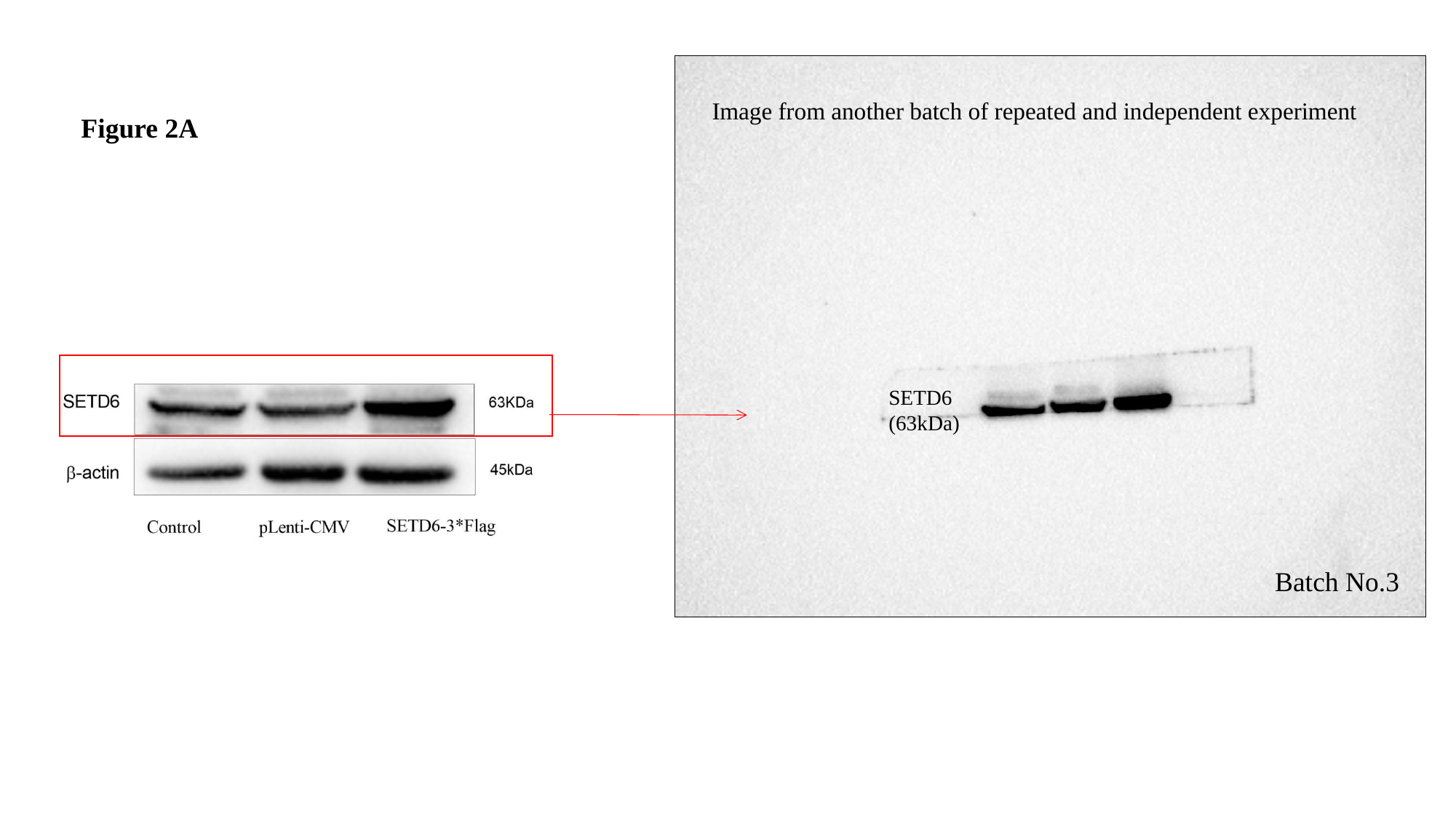

Image from another batch of repeated and independent experiment
Figure 2A
SETD6
(63kDa)
Batch No.3

## Slide 10
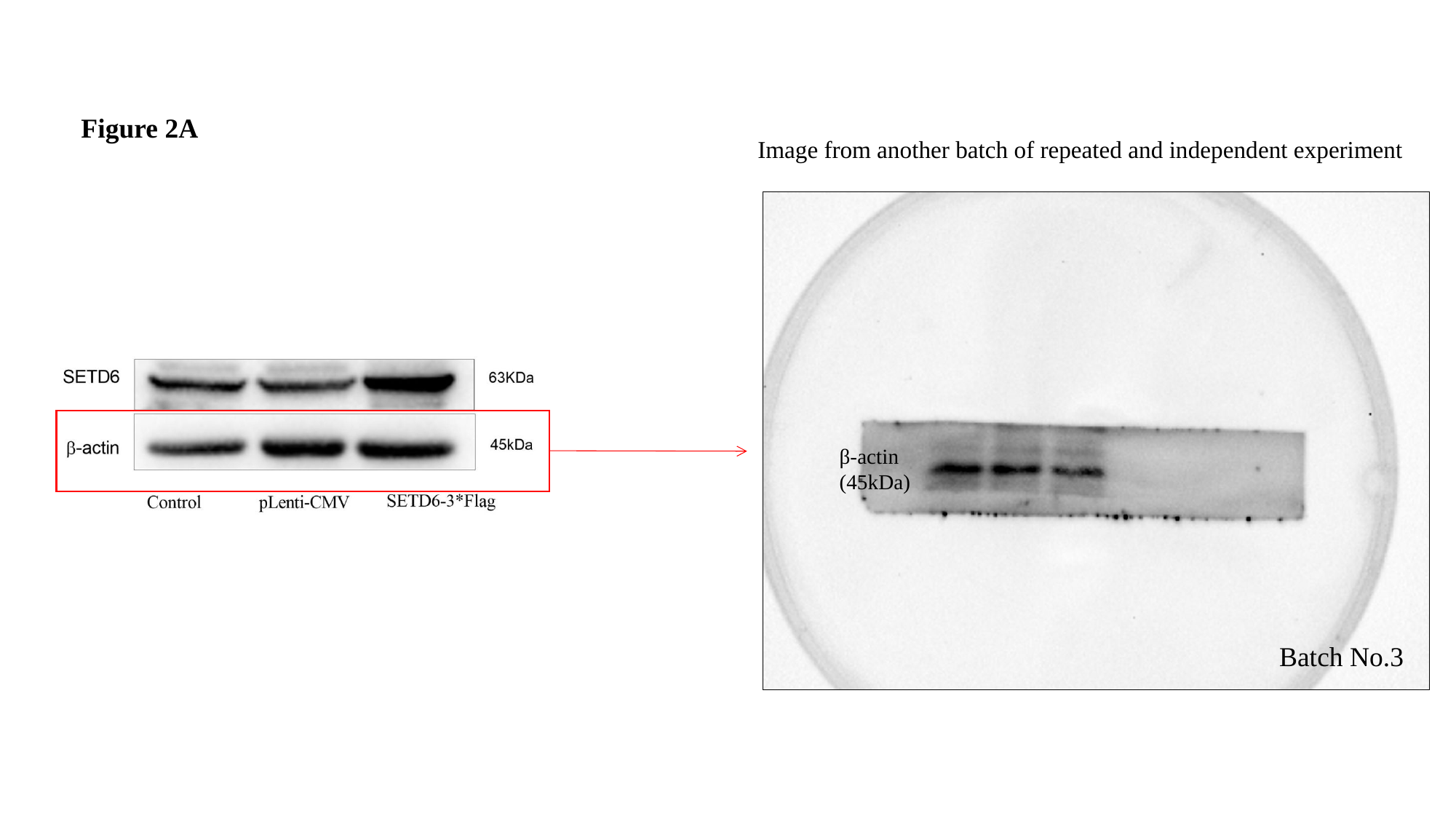

Figure 2A
Image from another batch of repeated and independent experiment
β-actin (45kDa)
Batch No.3

## Slide 11
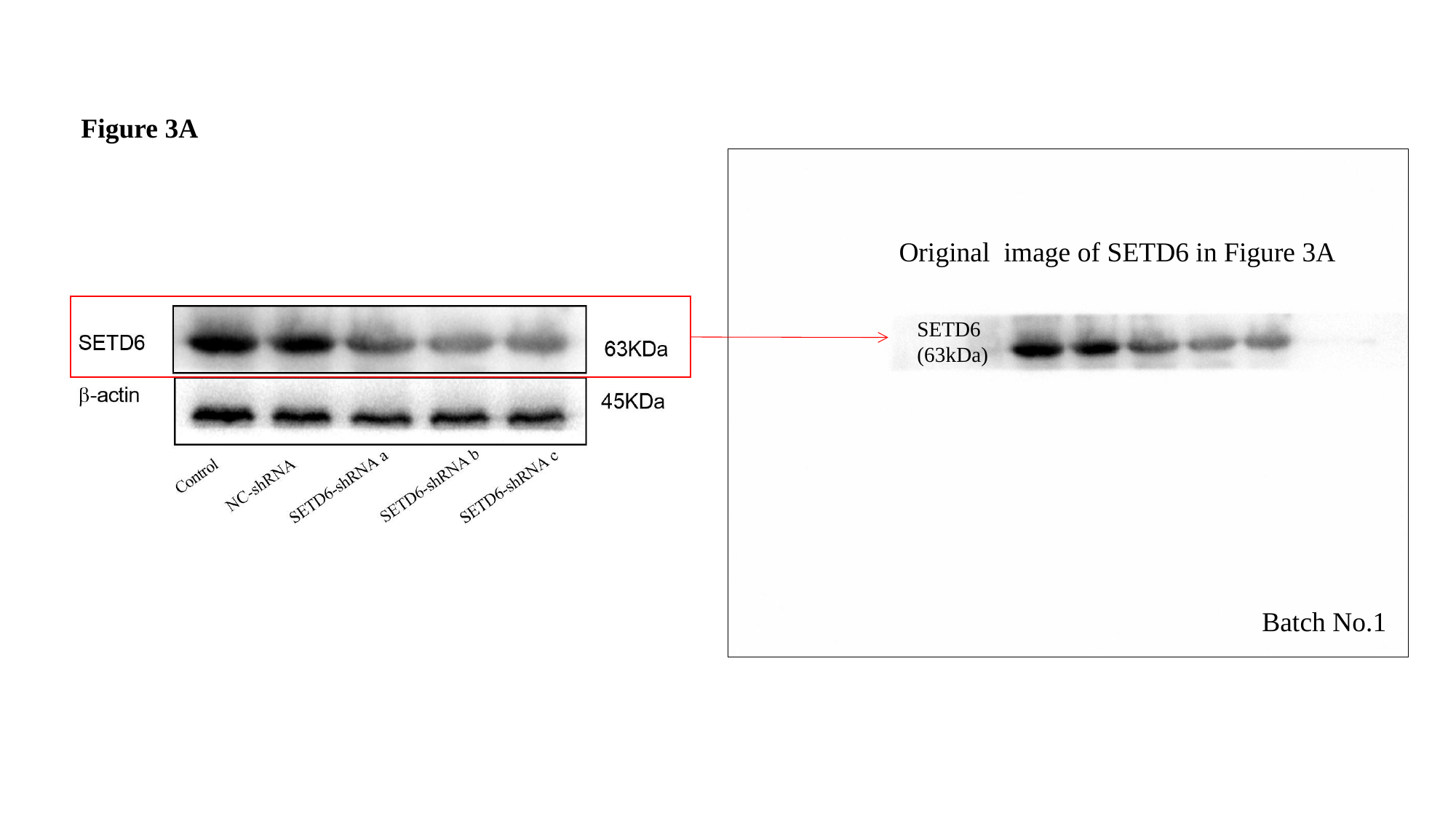

Figure 3A
Original image of SETD6 in Figure 3A
SETD6
(63kDa)
Batch No.1

## Slide 12
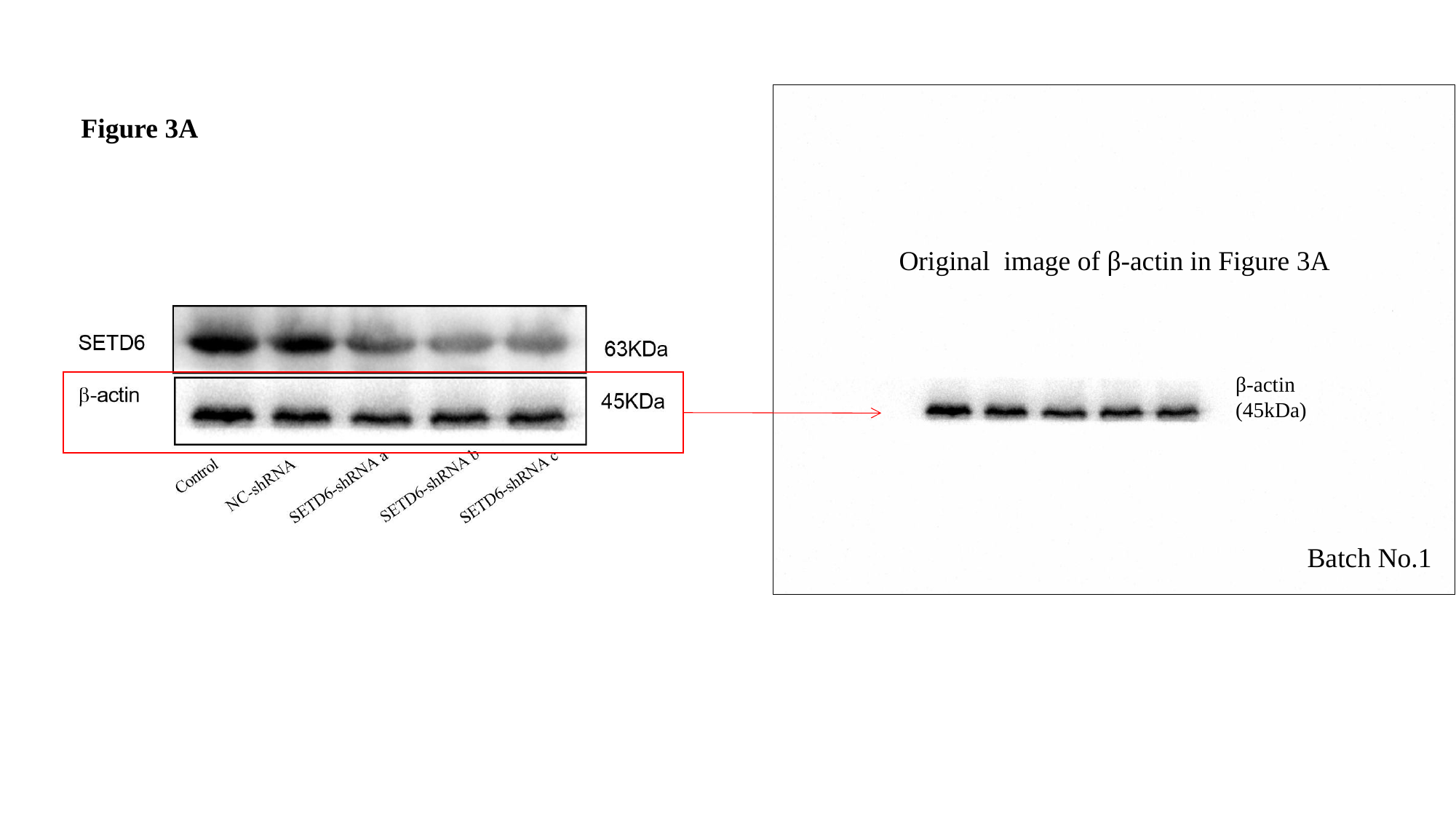

Figure 3A
Original image of β-actin in Figure 3A
β-actin (45kDa)
Batch No.1

## Slide 13
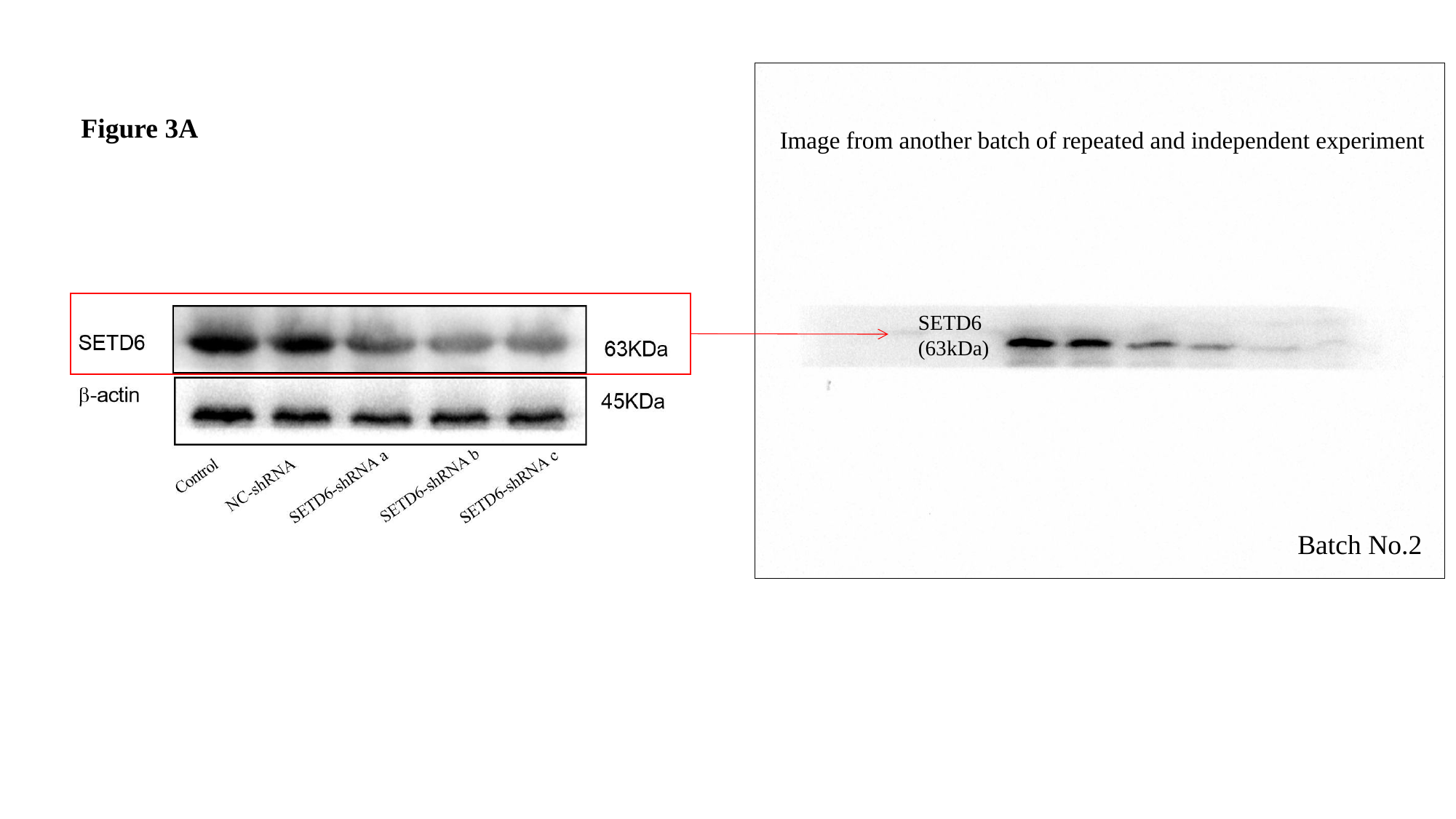

Figure 3A
Image from another batch of repeated and independent experiment
SETD6
(63kDa)
Batch No.2

## Slide 14
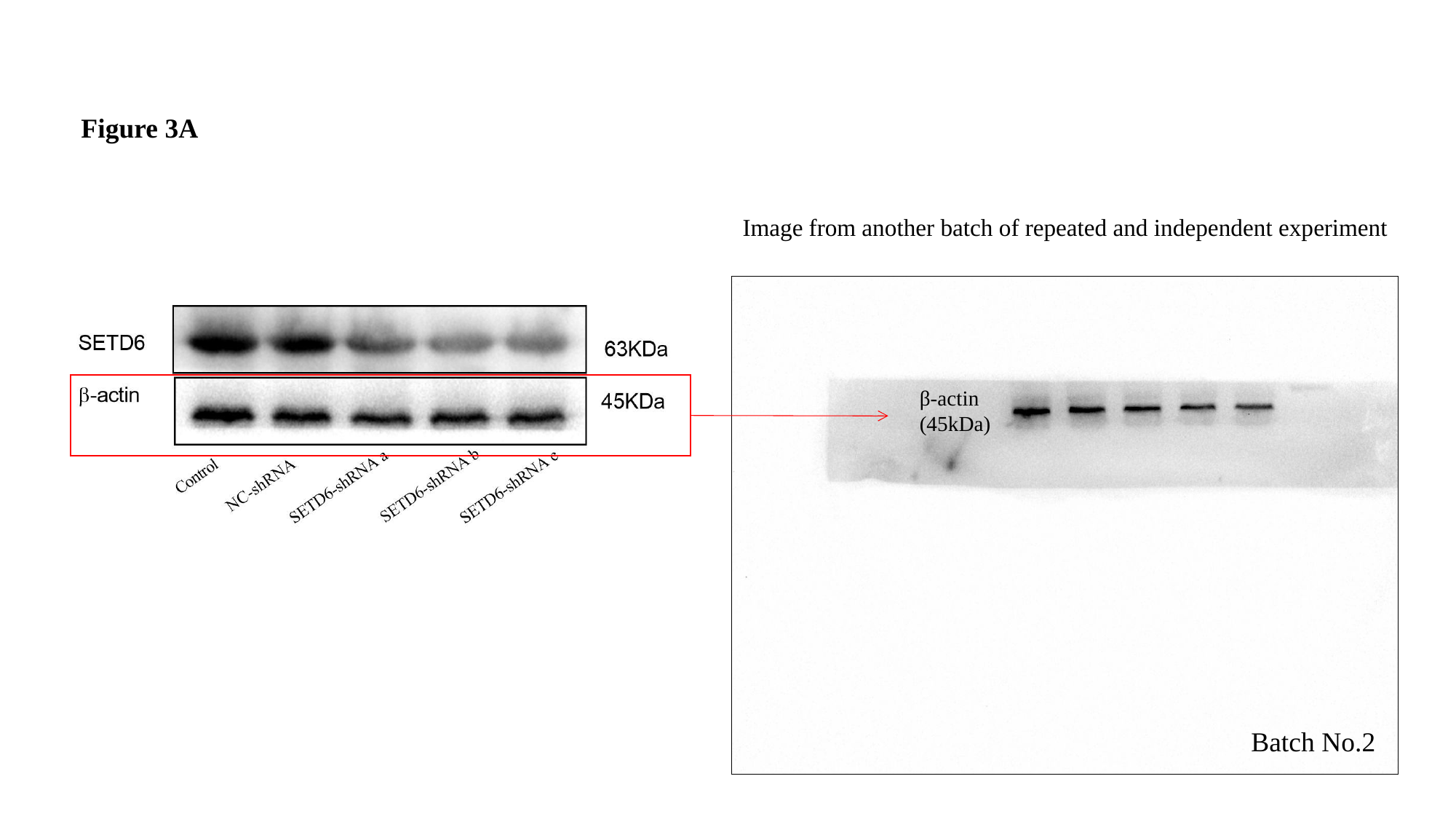

Figure 3A
Image from another batch of repeated and independent experiment
β-actin (45kDa)
Batch No.2

## Slide 15
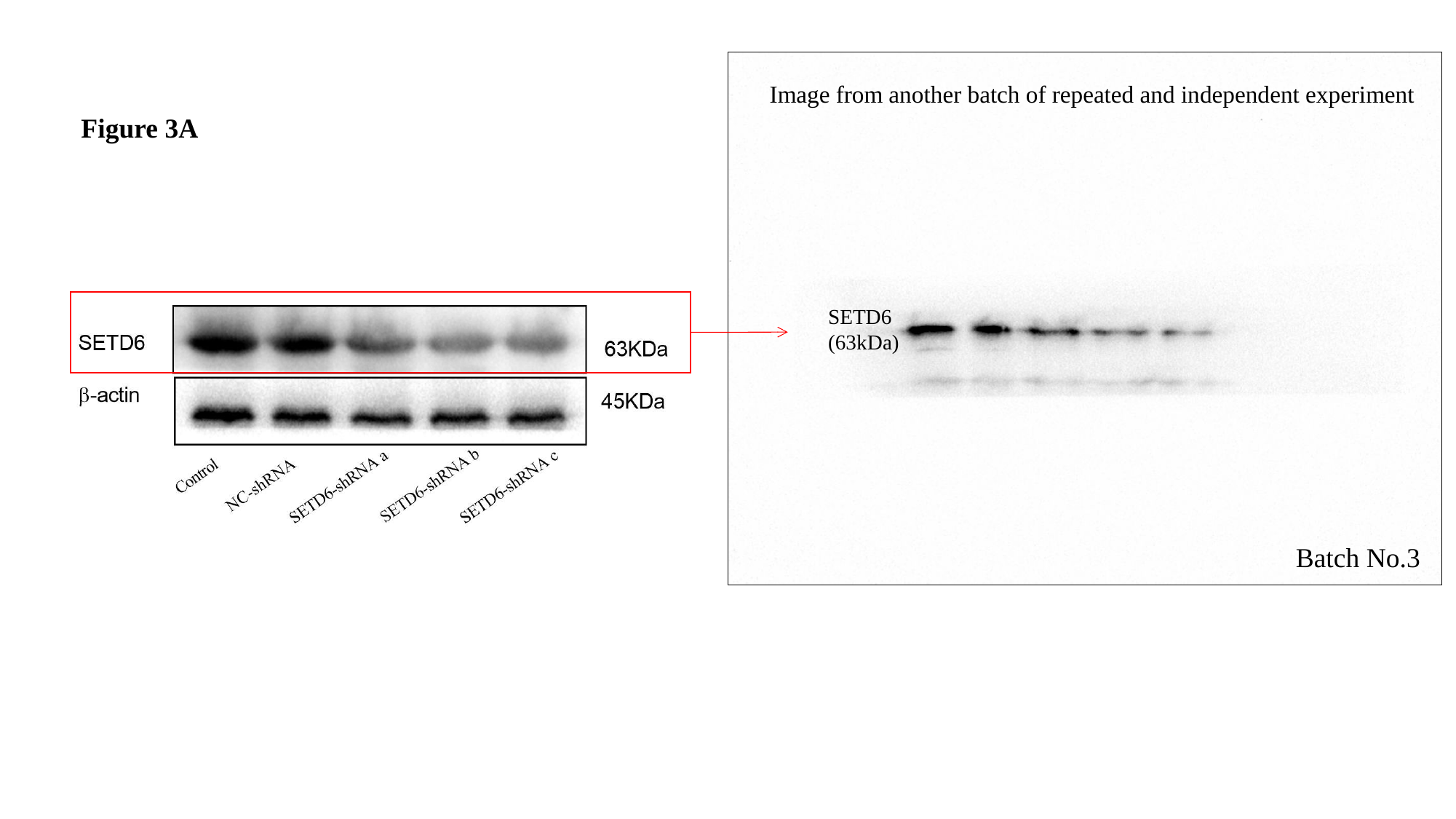

Image from another batch of repeated and independent experiment
Figure 3A
SETD6
(63kDa)
Batch No.3

## Slide 16
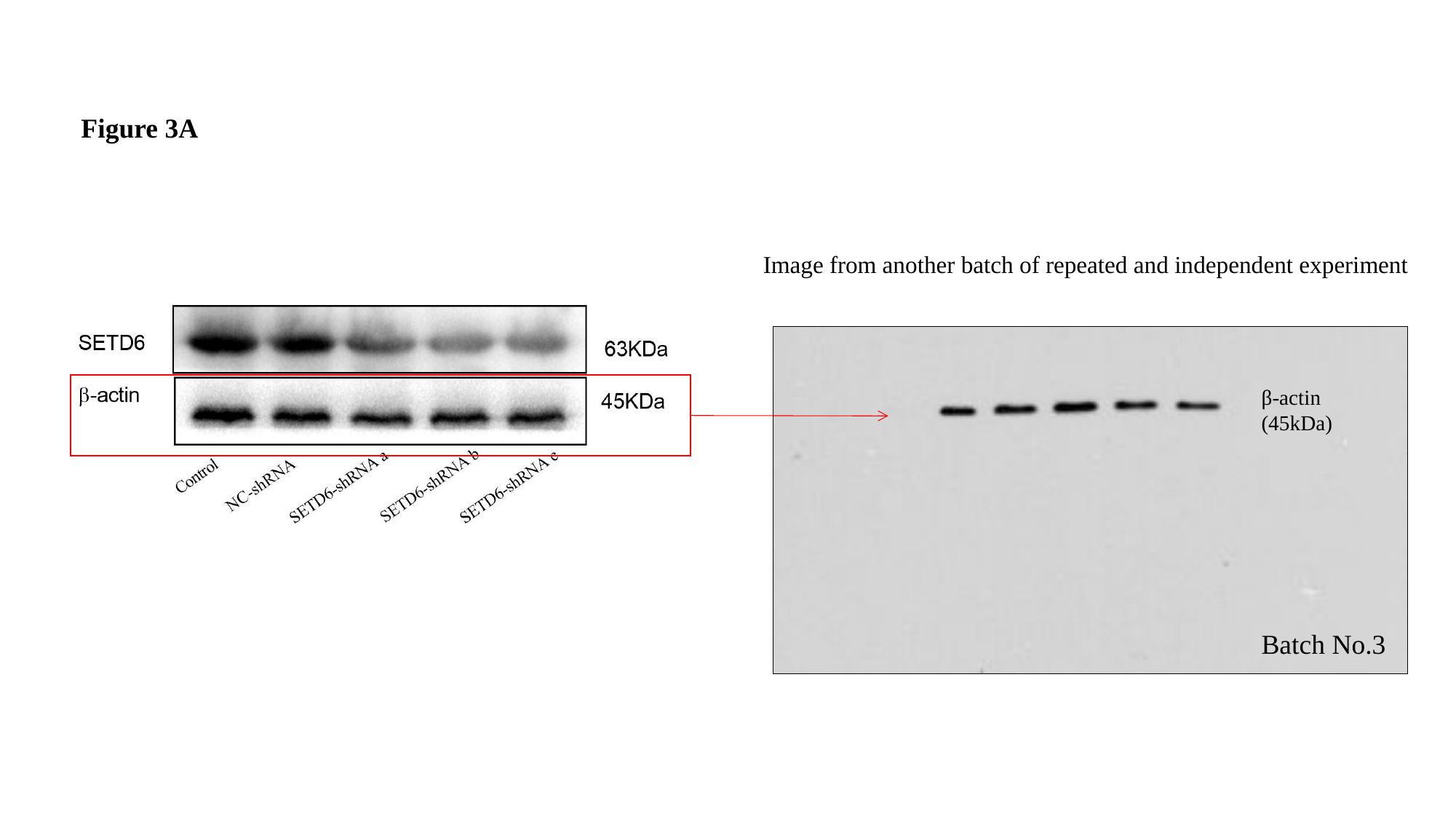

Figure 3A
Image from another batch of repeated and independent experiment
β-actin (45kDa)
Batch No.3

## Slide 17
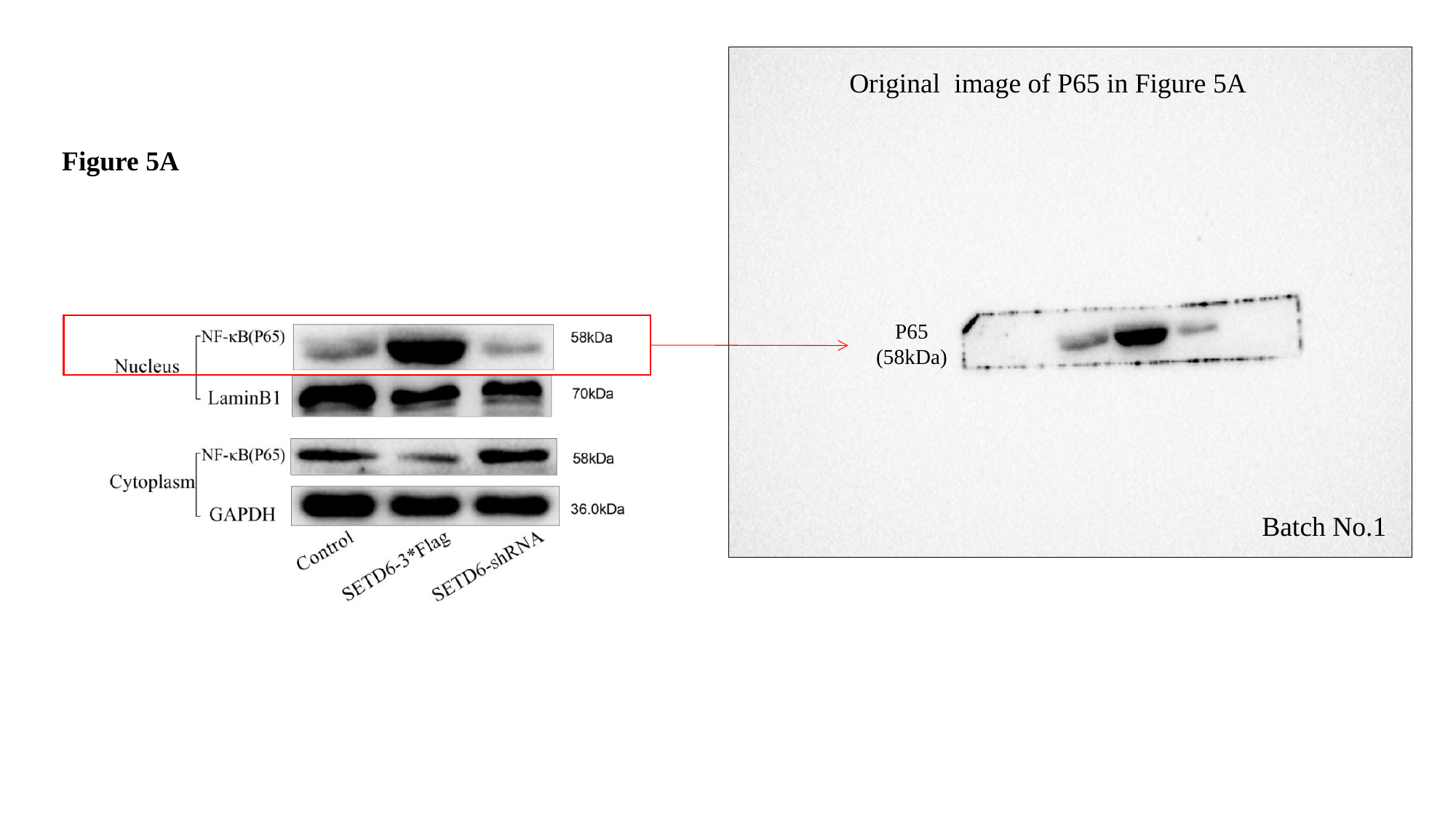

Original image of P65 in Figure 5A
Figure 5A
P65
(58kDa)
Batch No.1

## Slide 18
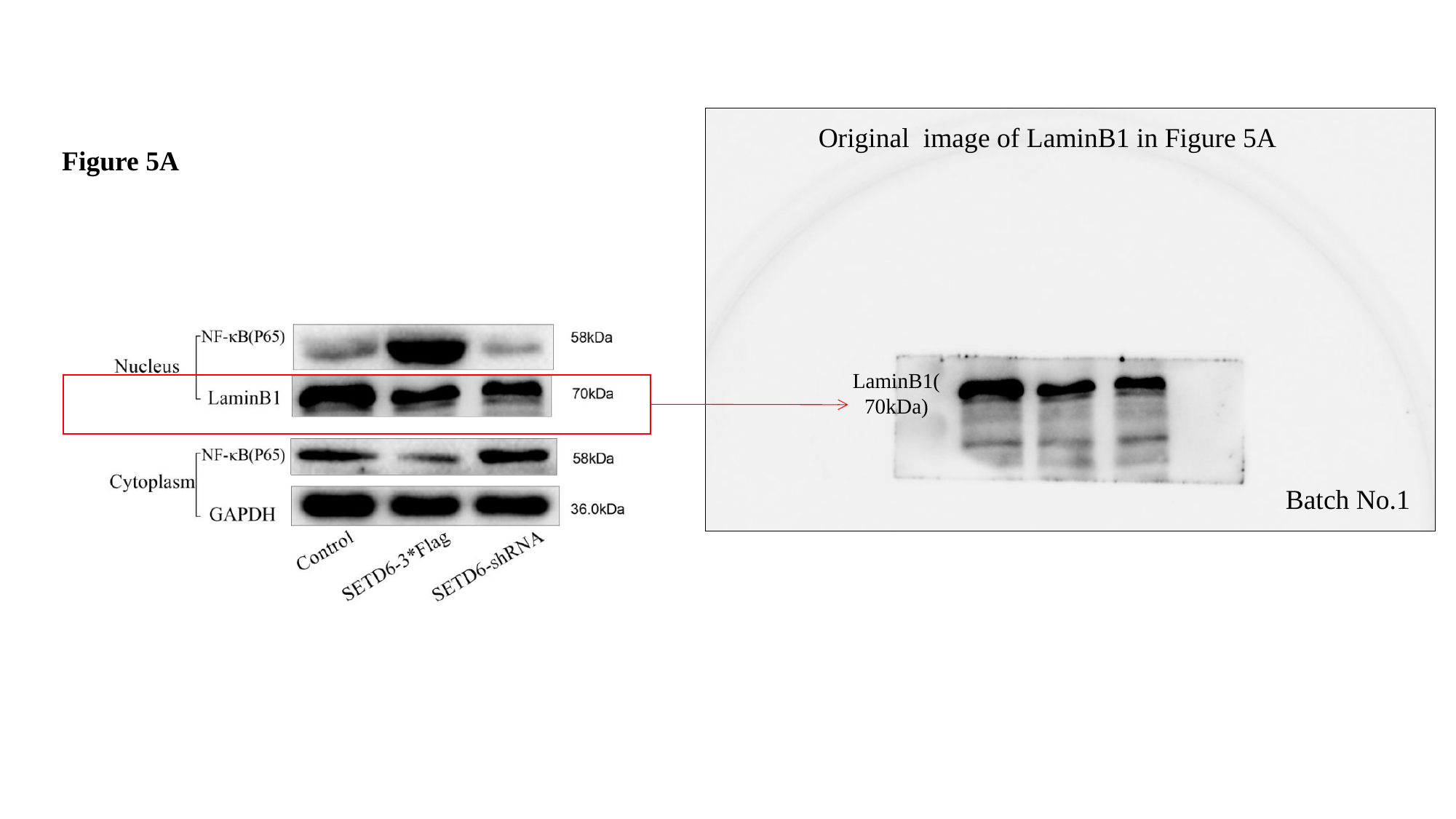

Original image of LaminB1 in Figure 5A
Figure 5A
LaminB1(70kDa)
Batch No.1

## Slide 19
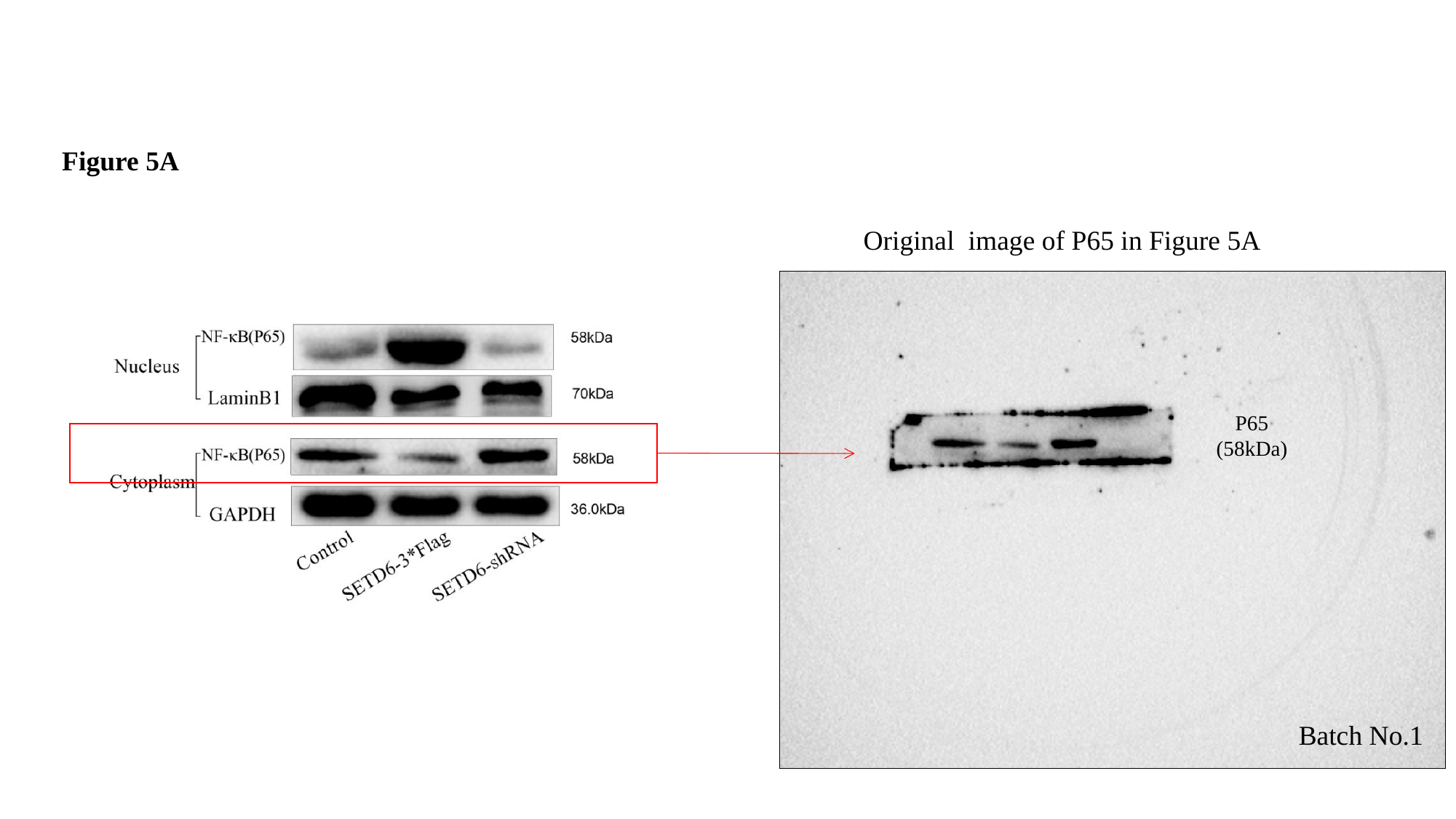

Figure 5A
Original image of P65 in Figure 5A
P65
(58kDa)
Batch No.1

## Slide 20
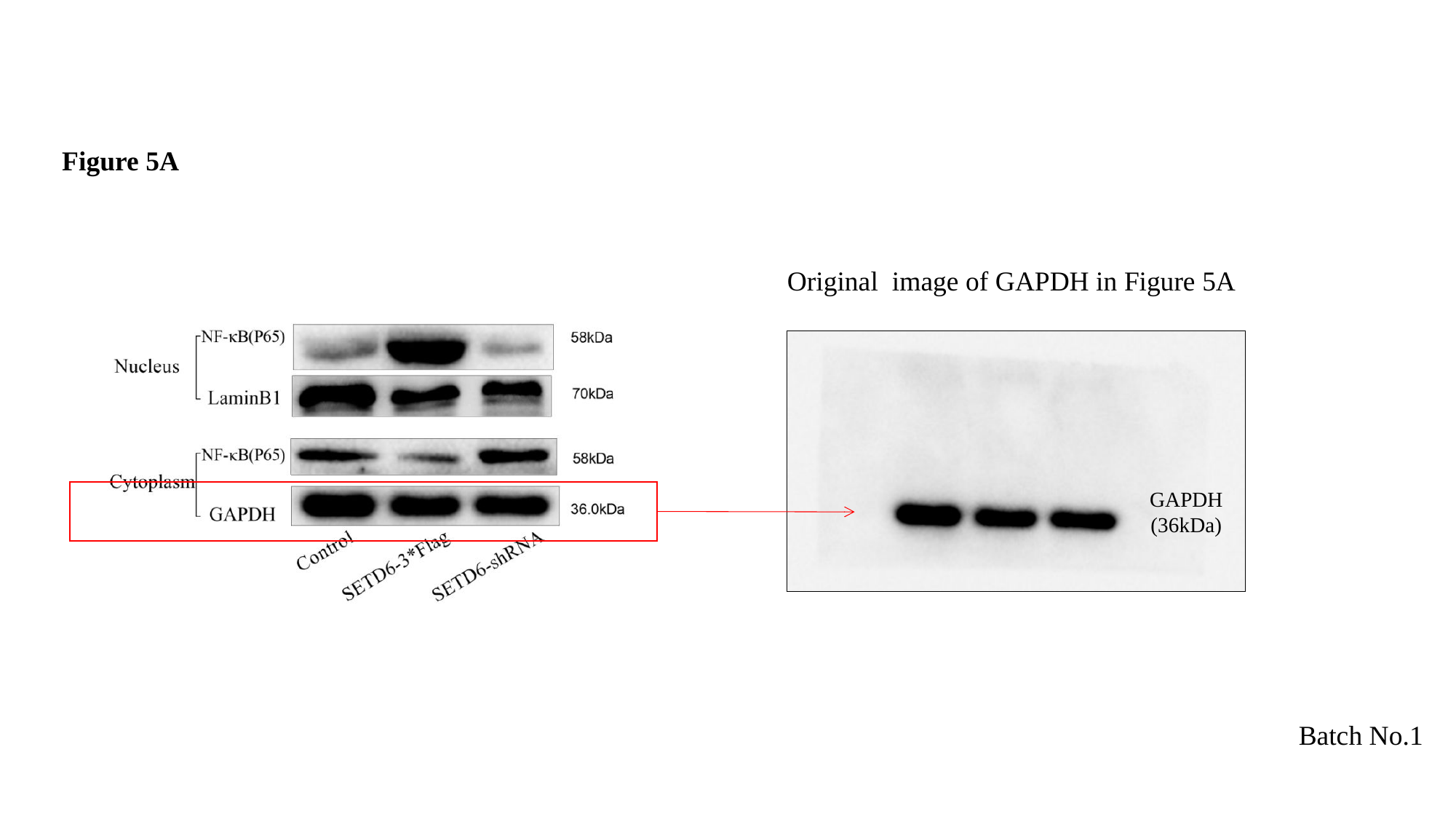

Figure 5A
Original image of GAPDH in Figure 5A
GAPDH
(36kDa)
Batch No.1

## Slide 21
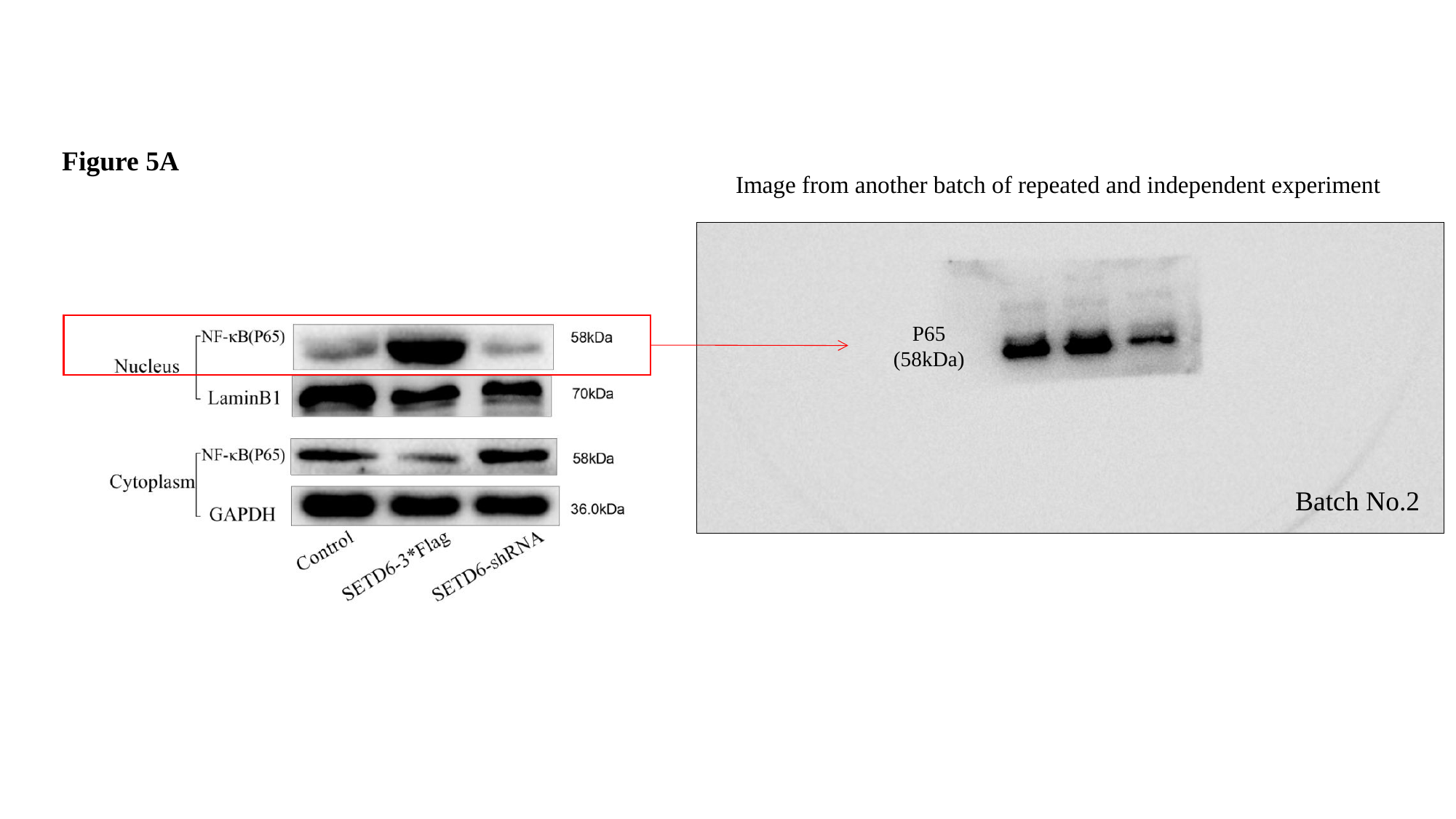

Figure 5A
Image from another batch of repeated and independent experiment
P65
(58kDa)
Batch No.2

## Slide 22
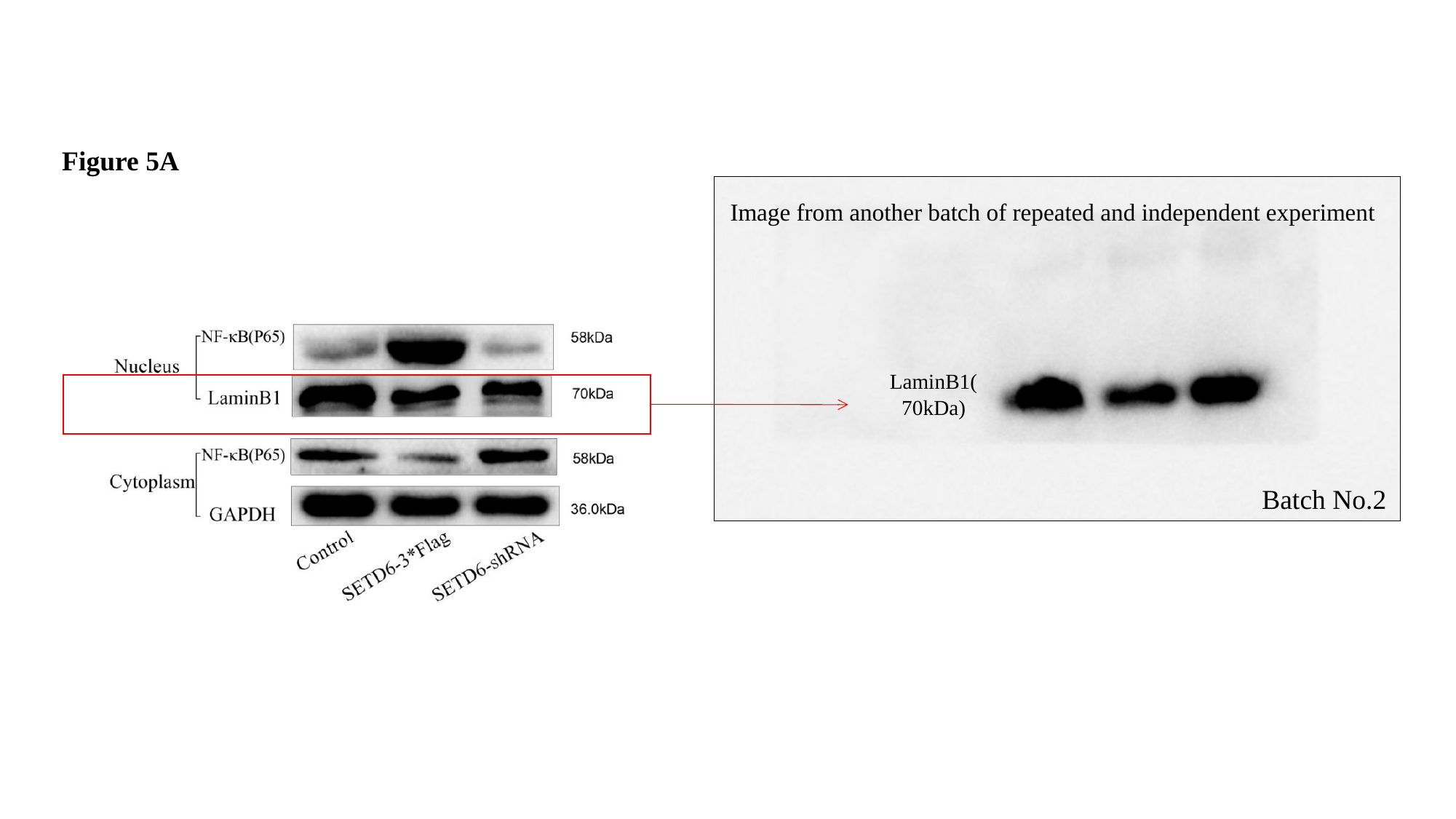

Figure 5A
Image from another batch of repeated and independent experiment
LaminB1(70kDa)
Batch No.2

## Slide 23
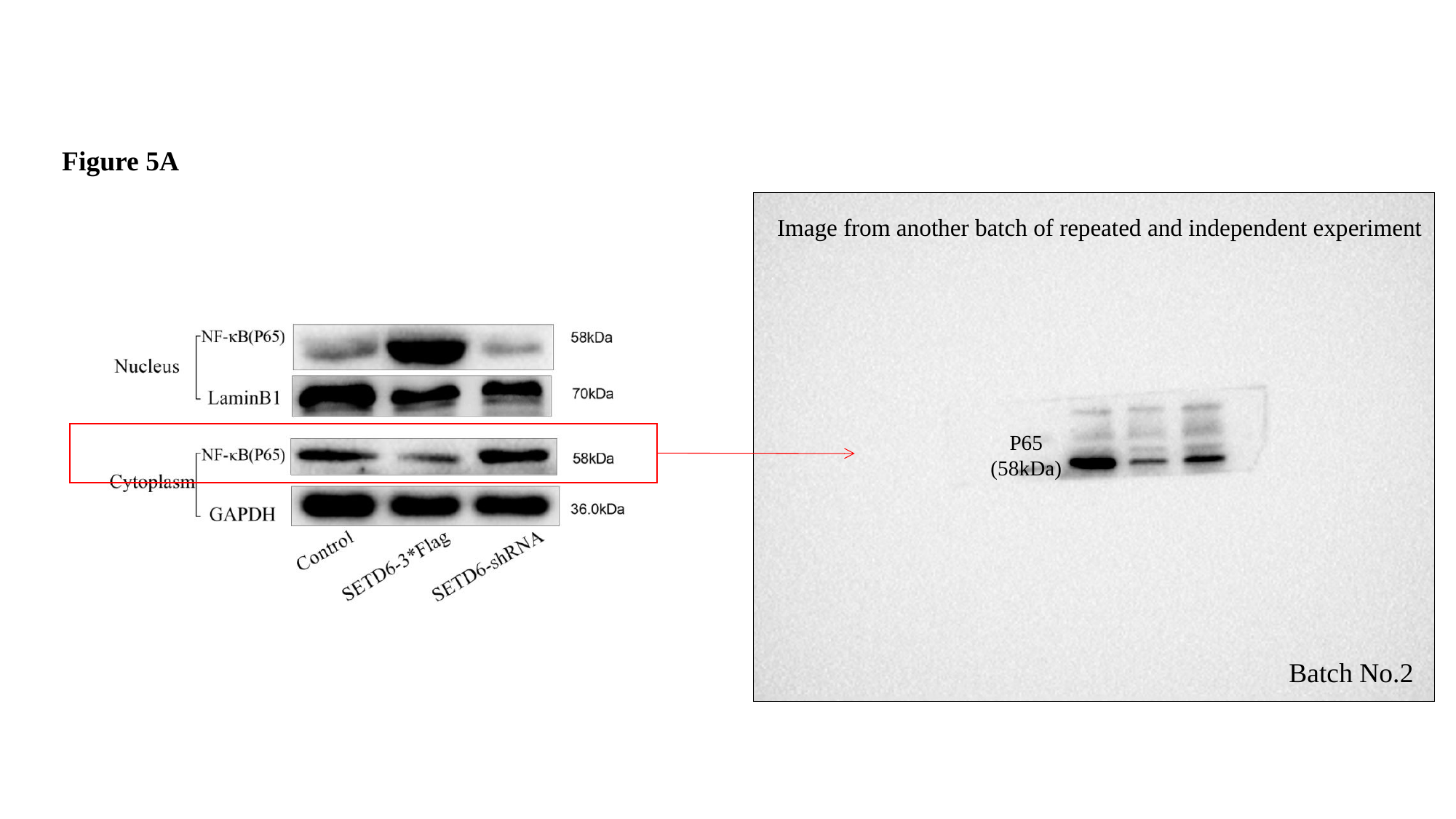

Figure 5A
Image from another batch of repeated and independent experiment
P65
(58kDa)
Batch No.2

## Slide 24
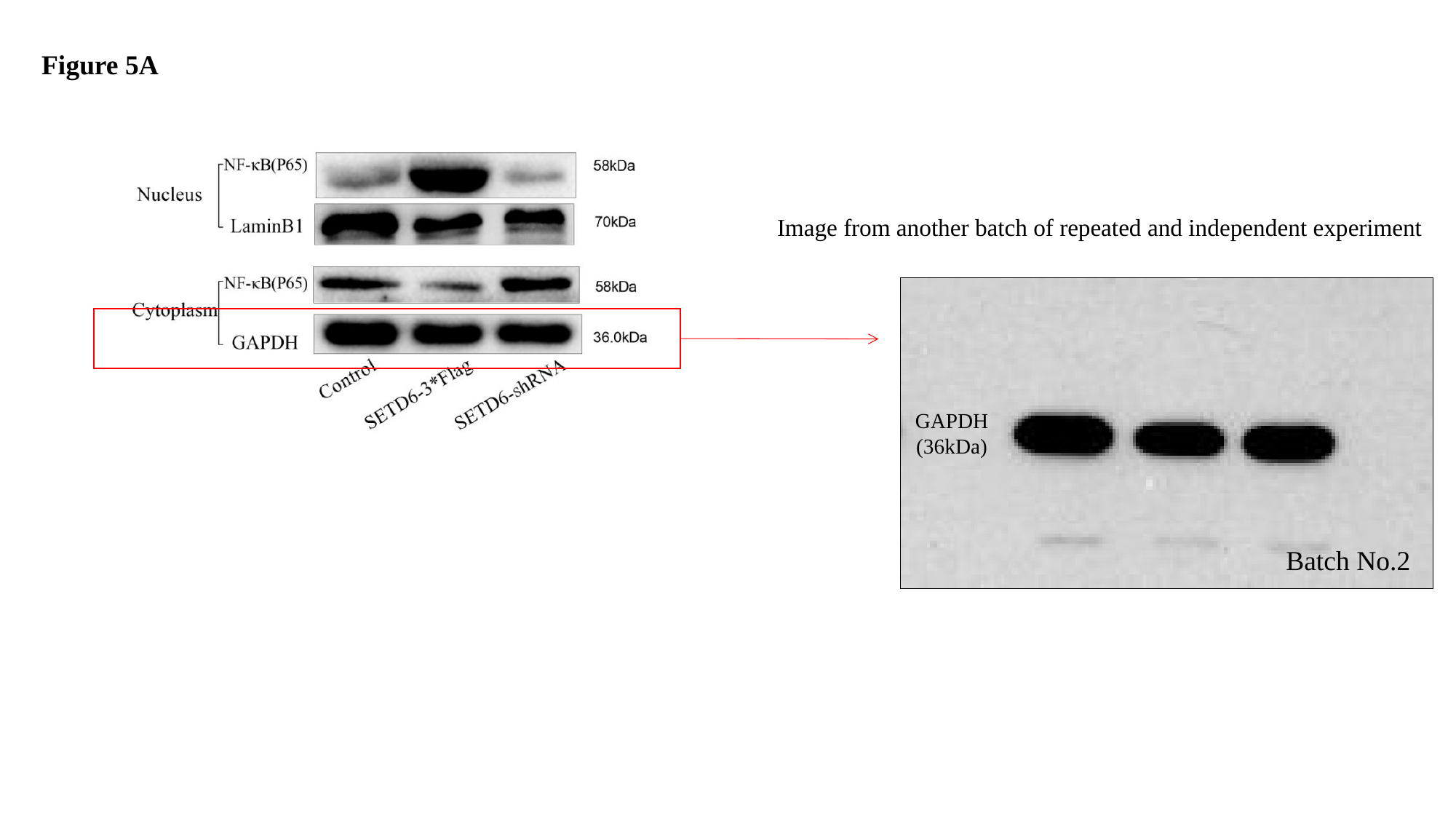

Figure 5A
Image from another batch of repeated and independent experiment
GAPDH
(36kDa)
Batch No.2

## Slide 25
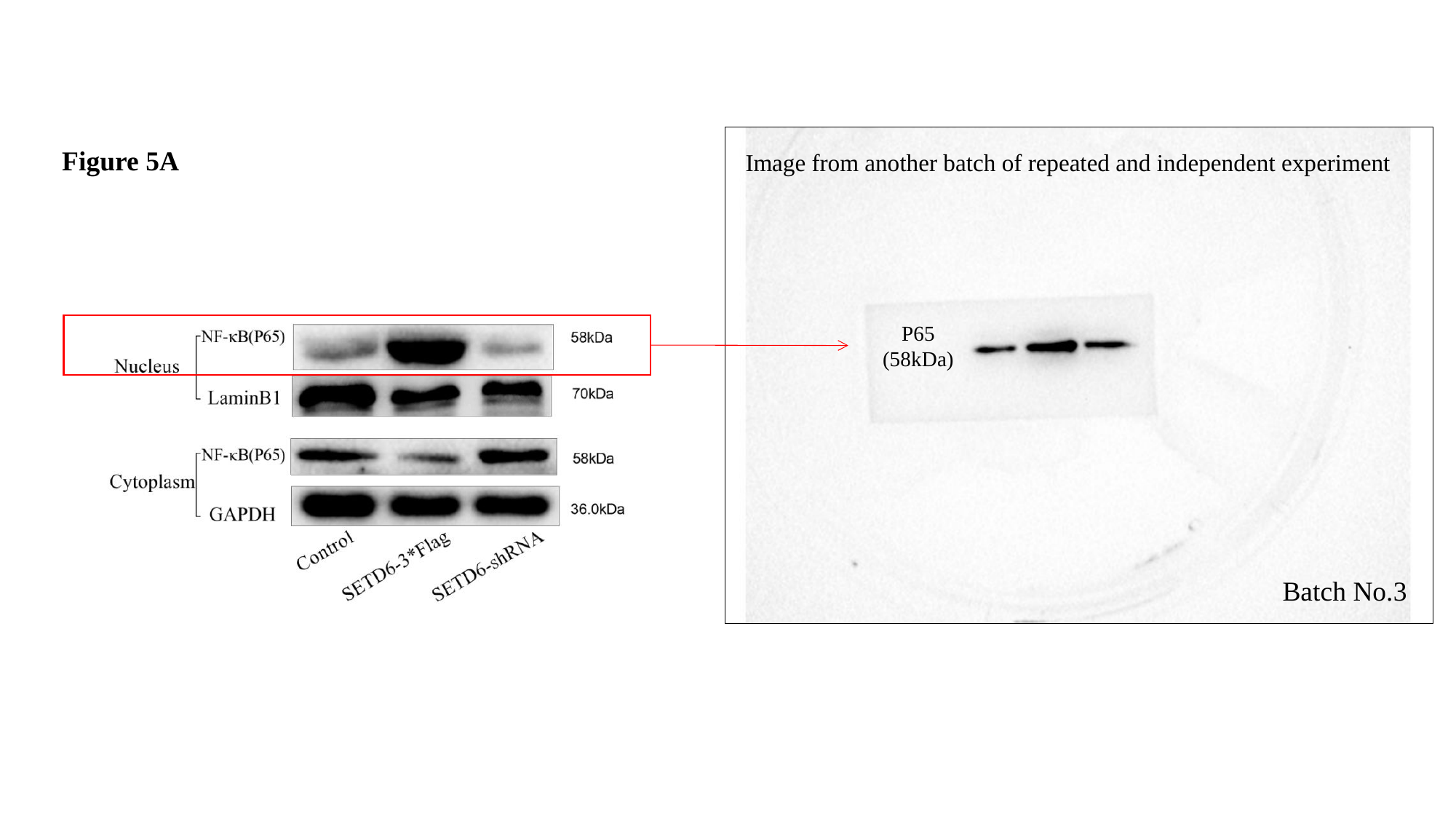

Figure 5A
Image from another batch of repeated and independent experiment
P65
(58kDa)
Batch No.3

## Slide 26
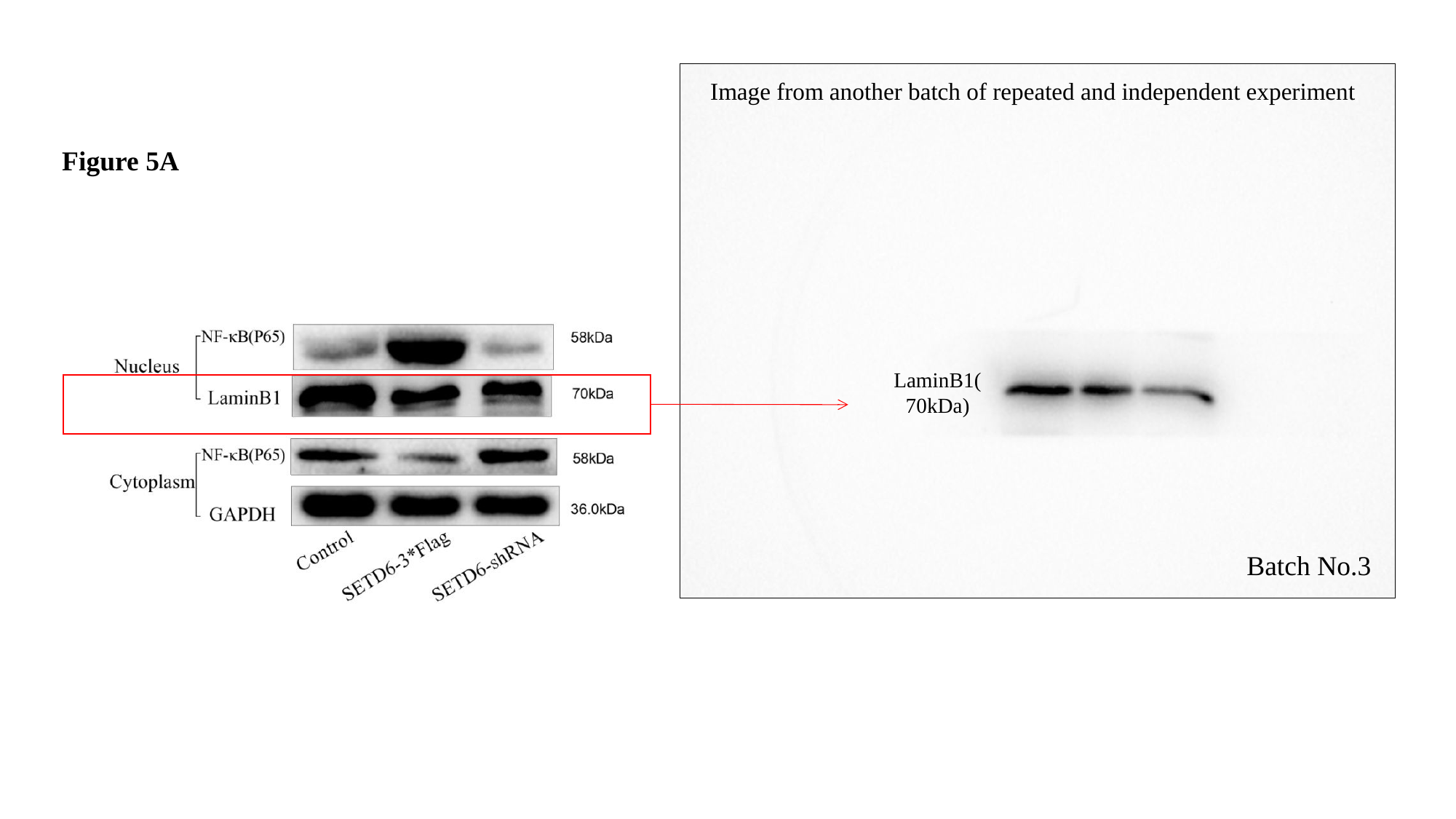

Image from another batch of repeated and independent experiment
Figure 5A
LaminB1(70kDa)
Batch No.3

## Slide 27
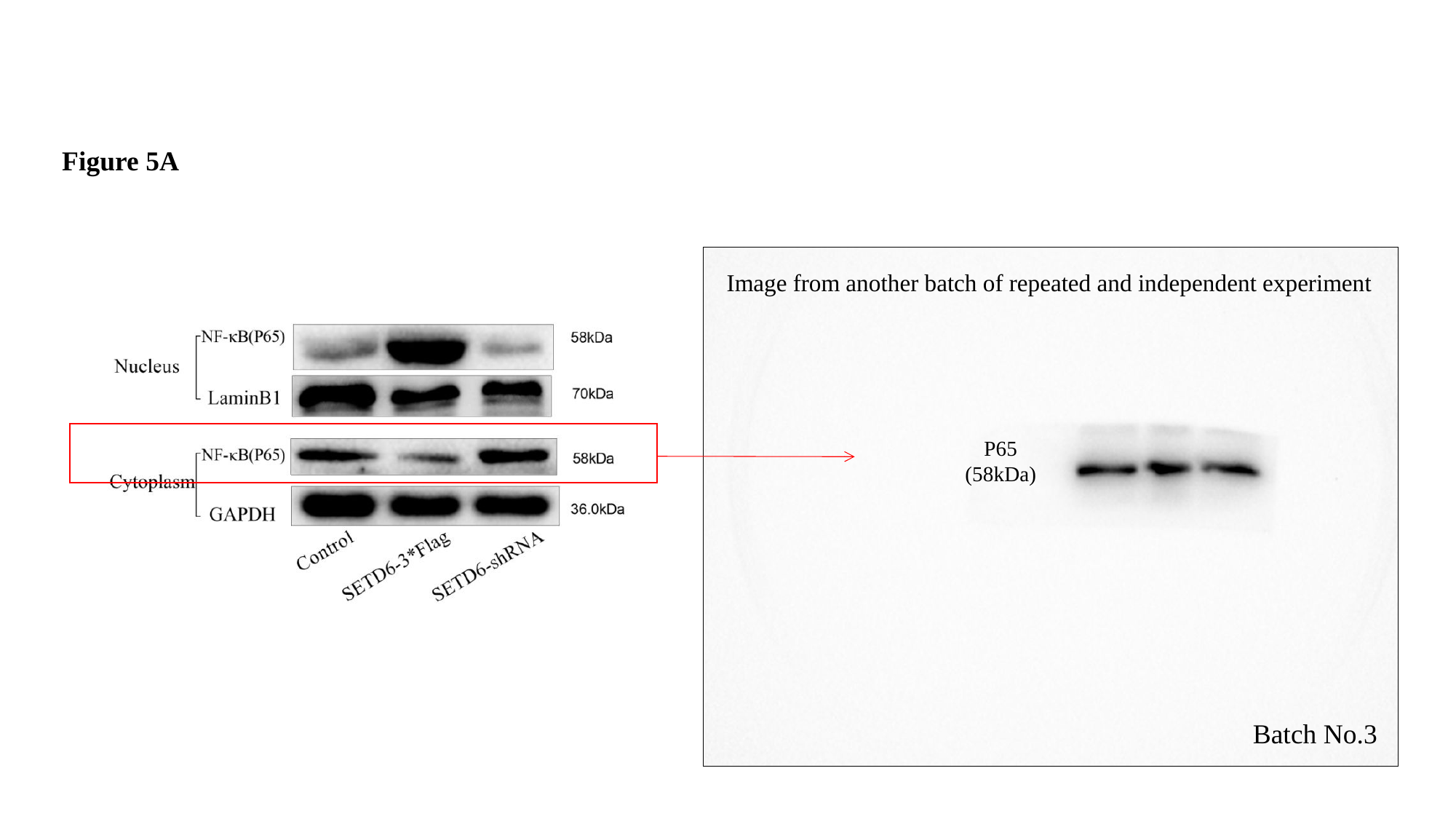

Figure 5A
Image from another batch of repeated and independent experiment
P65
(58kDa)
Batch No.3

## Slide 28
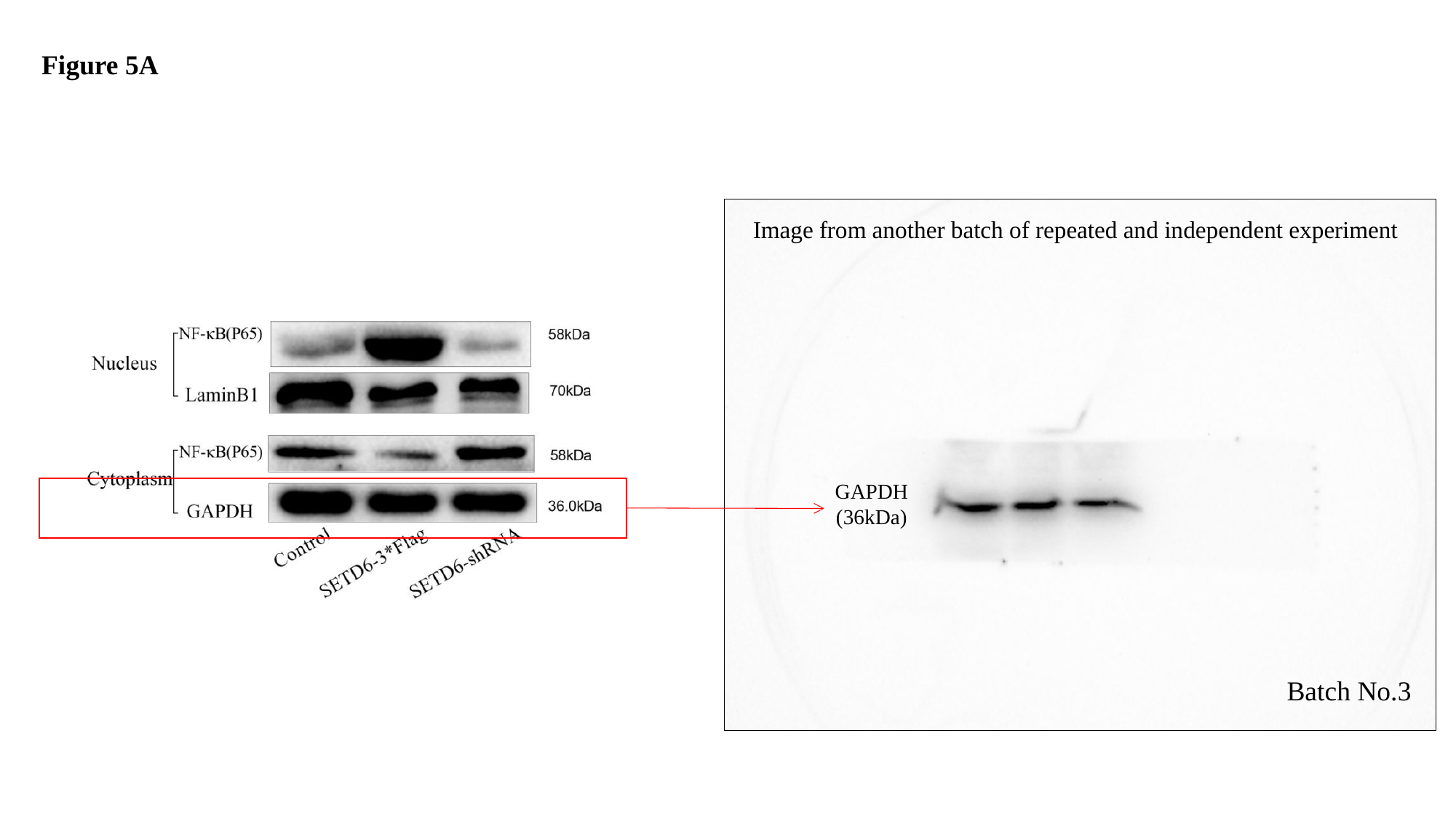

Figure 5A
Image from another batch of repeated and independent experiment
GAPDH
(36kDa)
Batch No.3

## Slide 29
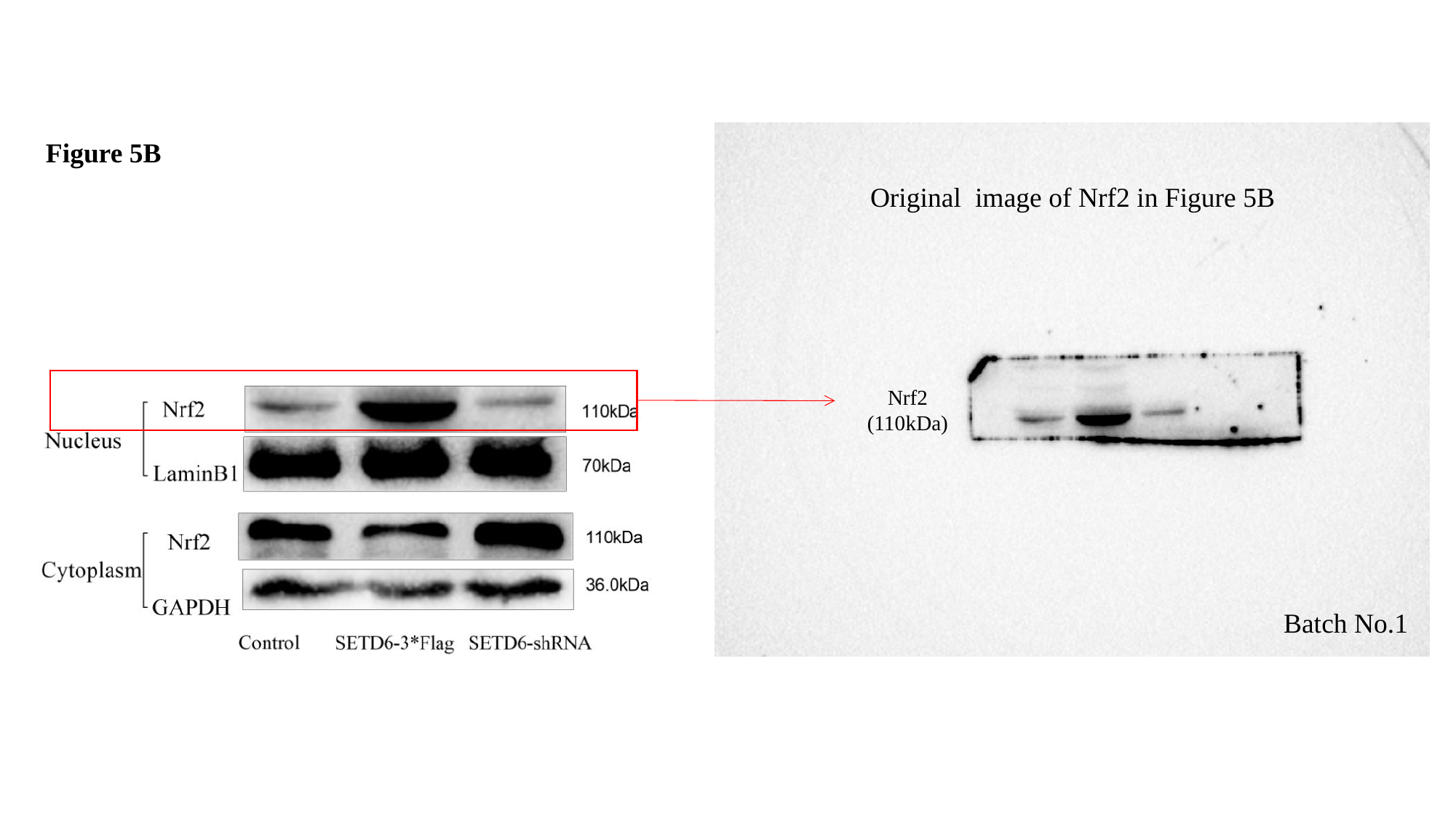

Figure 5B
Original image of Nrf2 in Figure 5B
Nrf2
(110kDa)
Batch No.1

## Slide 30
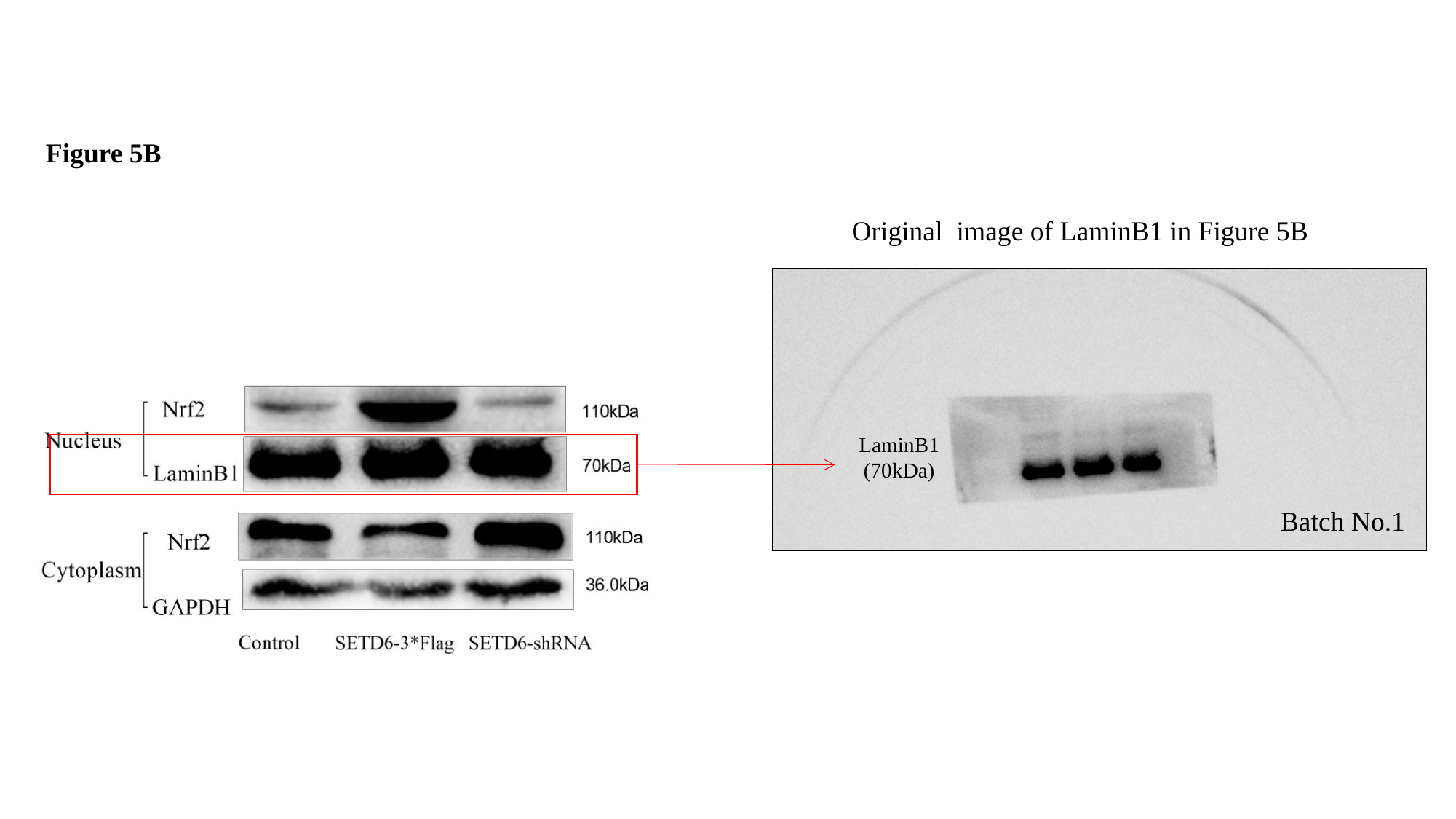

Figure 5B
Original image of LaminB1 in Figure 5B
LaminB1
(70kDa)
Batch No.1

## Slide 31
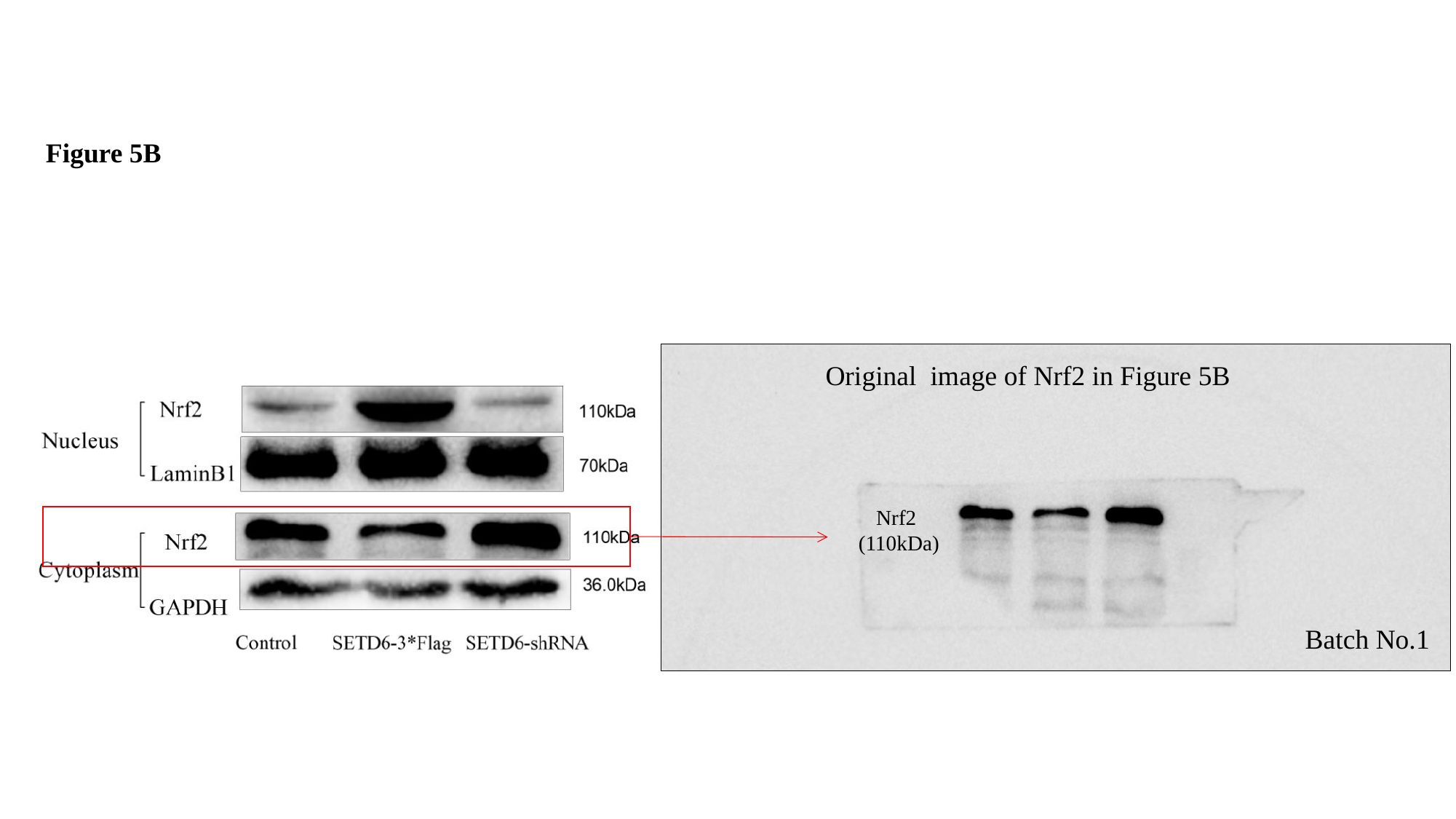

Figure 5B
Original image of Nrf2 in Figure 5B
Nrf2
(110kDa)
Batch No.1

## Slide 32
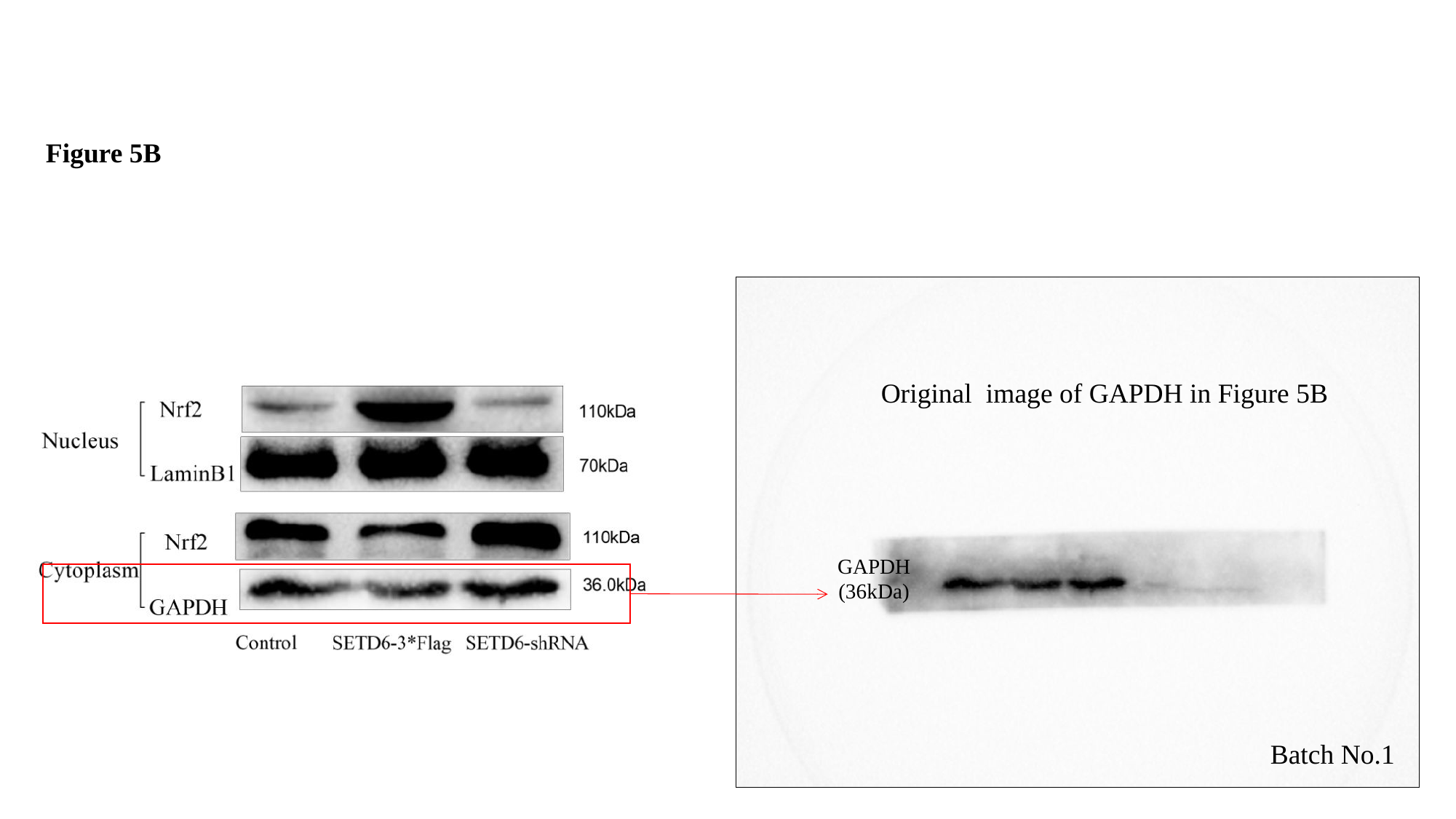

Figure 5B
Original image of GAPDH in Figure 5B
GAPDH
(36kDa)
Batch No.1

## Slide 33
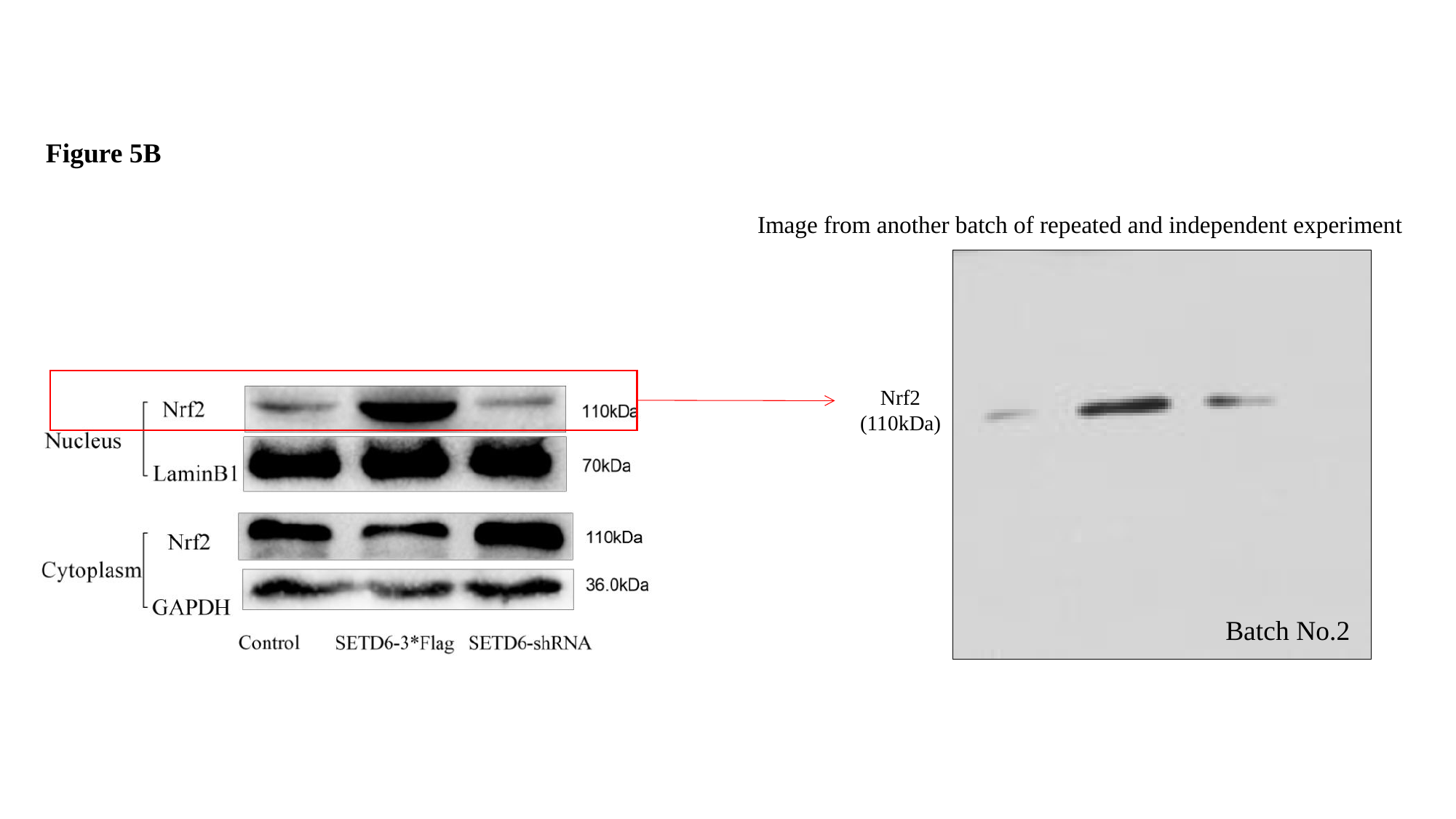

Figure 5B
Image from another batch of repeated and independent experiment
Nrf2
(110kDa)
Batch No.2

## Slide 34
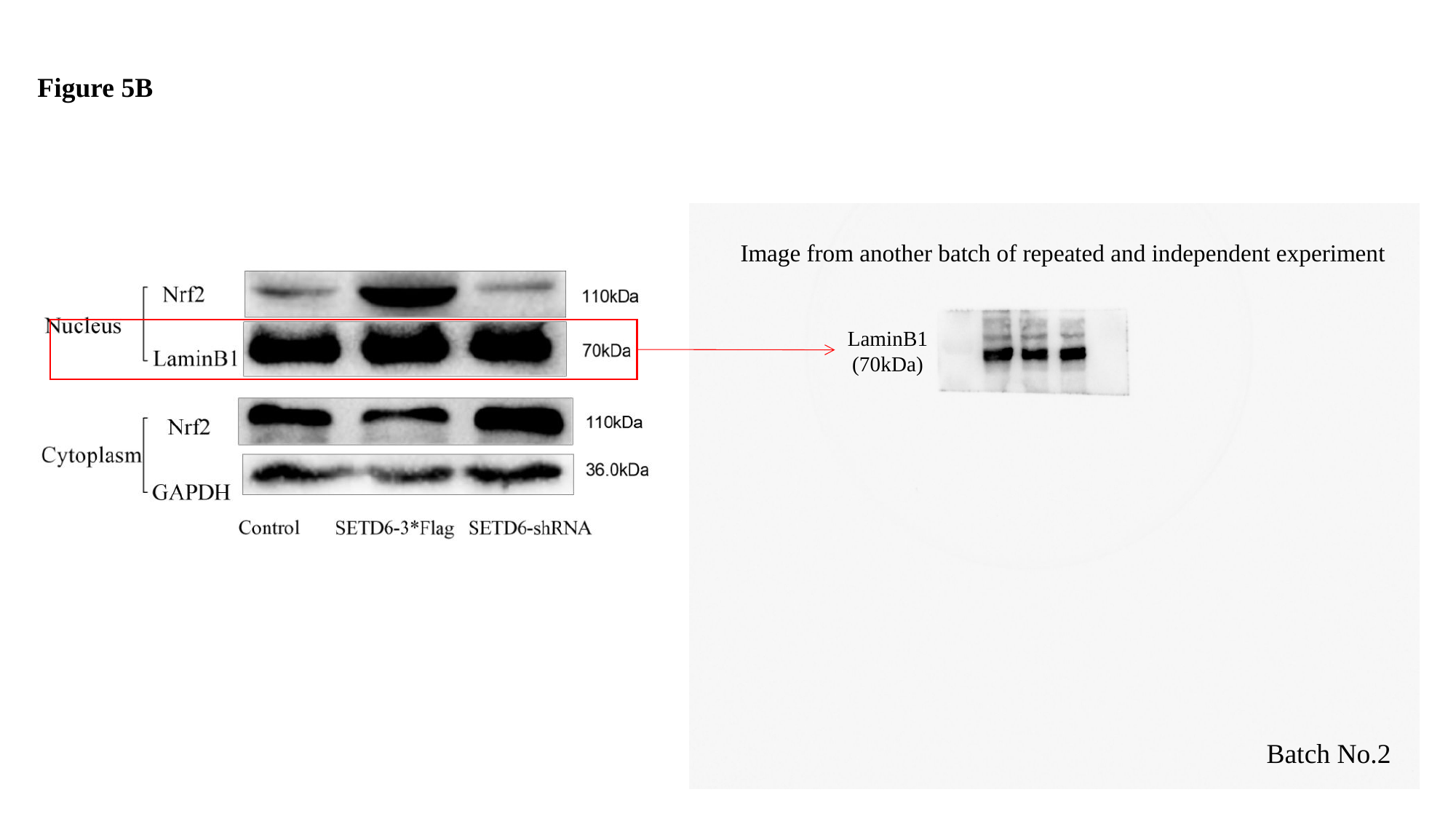

Figure 5B
Image from another batch of repeated and independent experiment
LaminB1
(70kDa)
Batch No.2

## Slide 35
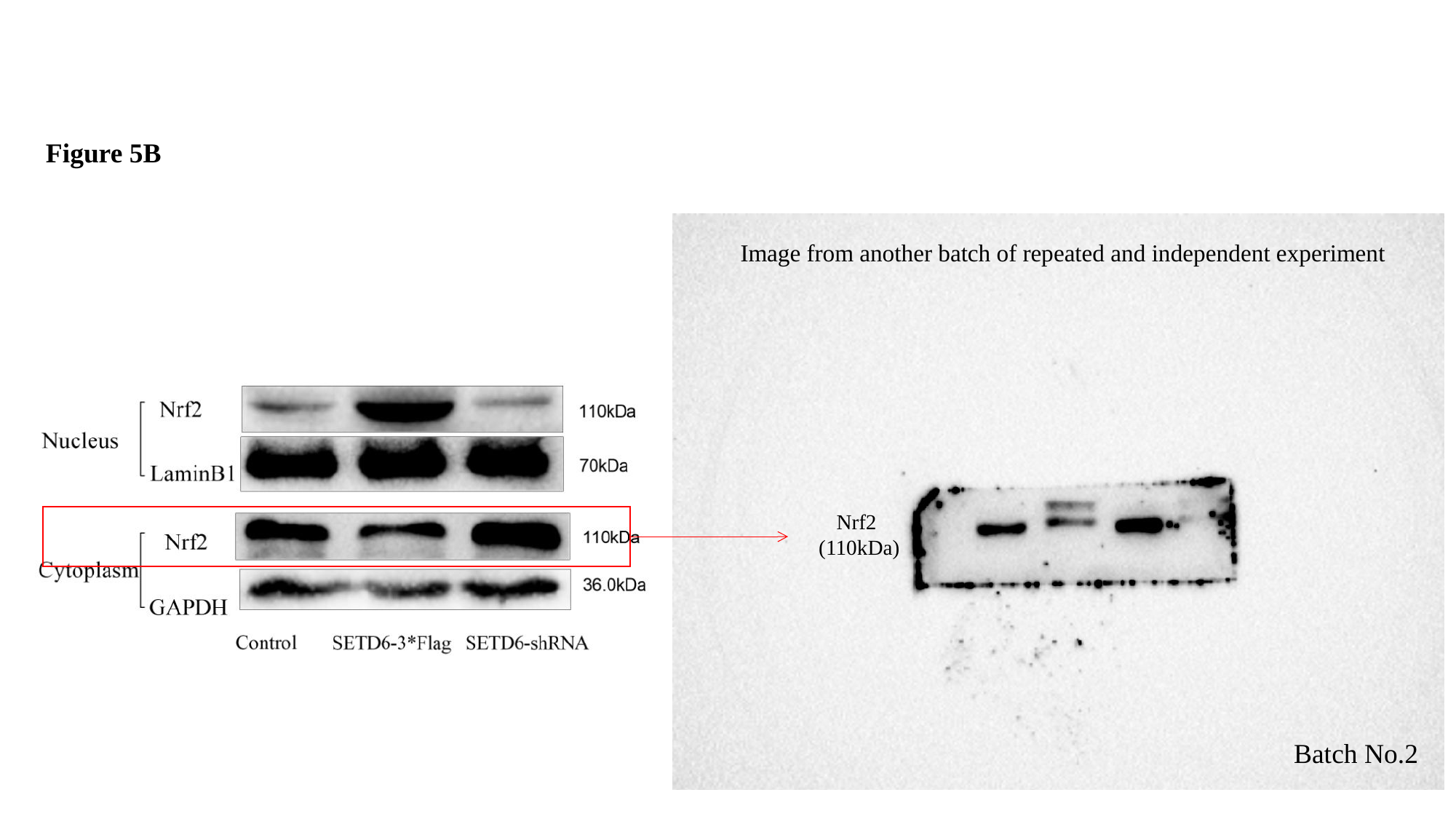

Figure 5B
Image from another batch of repeated and independent experiment
Nrf2
(110kDa)
Batch No.2

## Slide 36
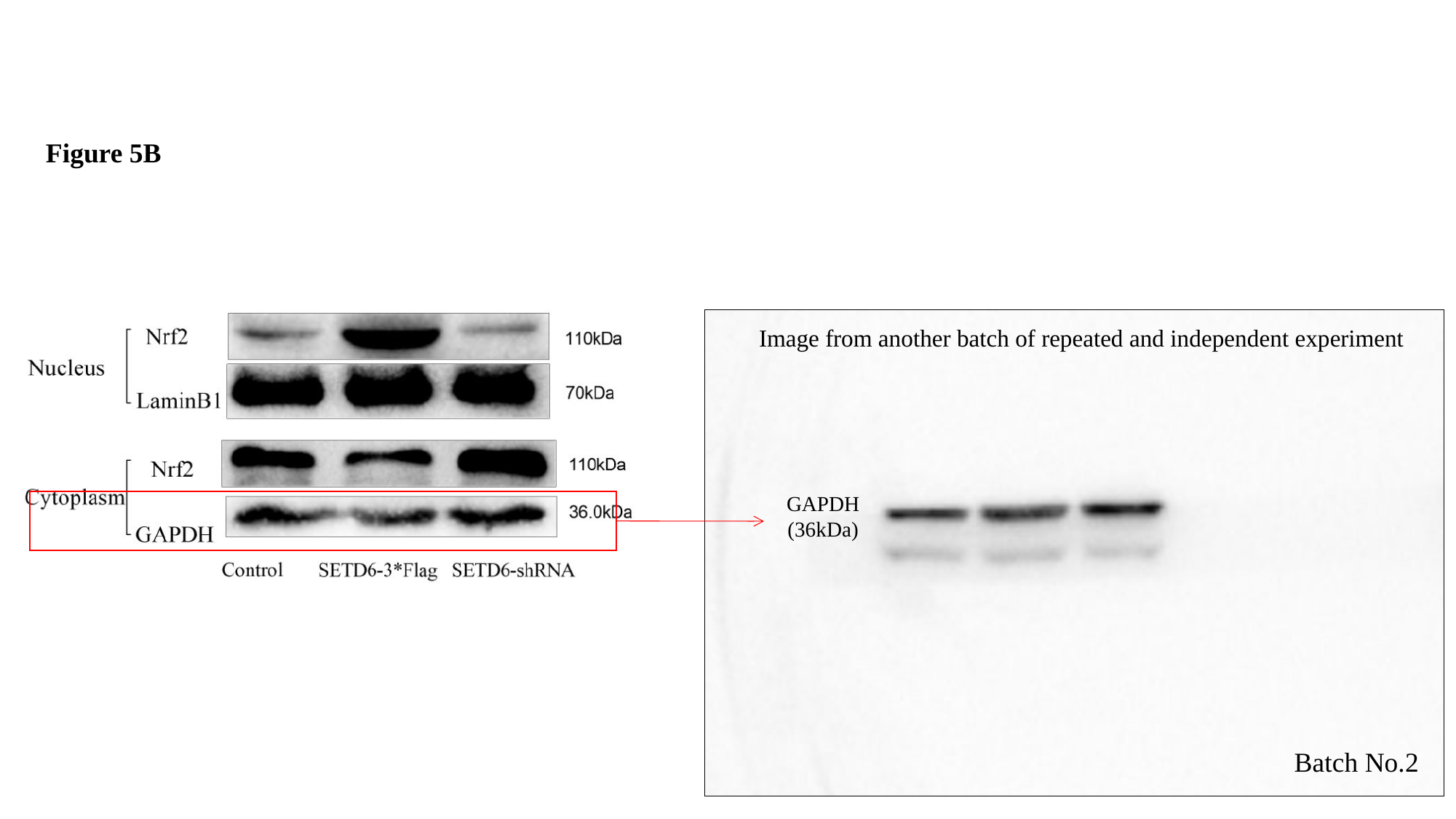

Figure 5B
Image from another batch of repeated and independent experiment
GAPDH
(36kDa)
Batch No.2

## Slide 37
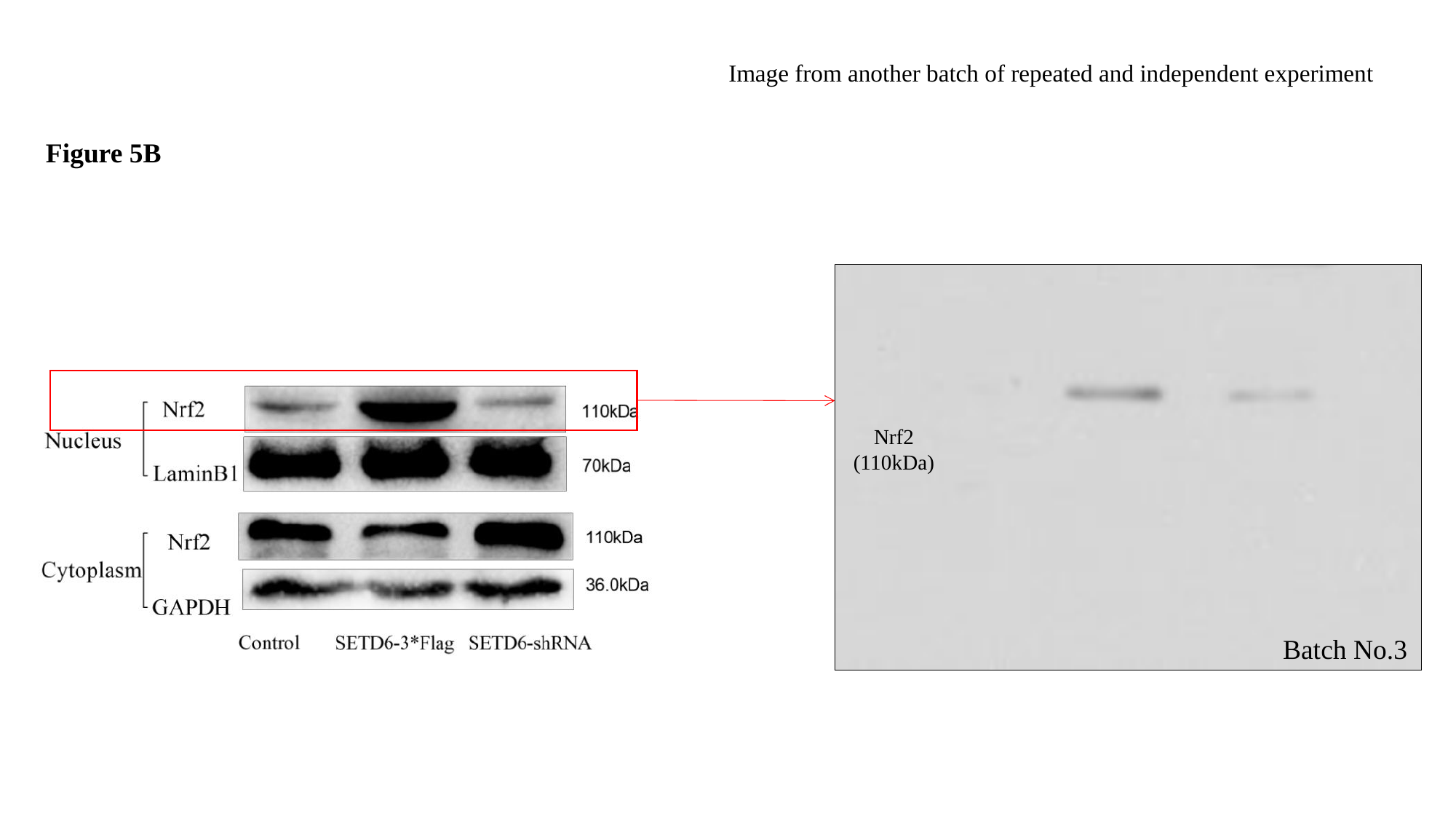

Image from another batch of repeated and independent experiment
Figure 5B
Nrf2
(110kDa)
Batch No.3

## Slide 38
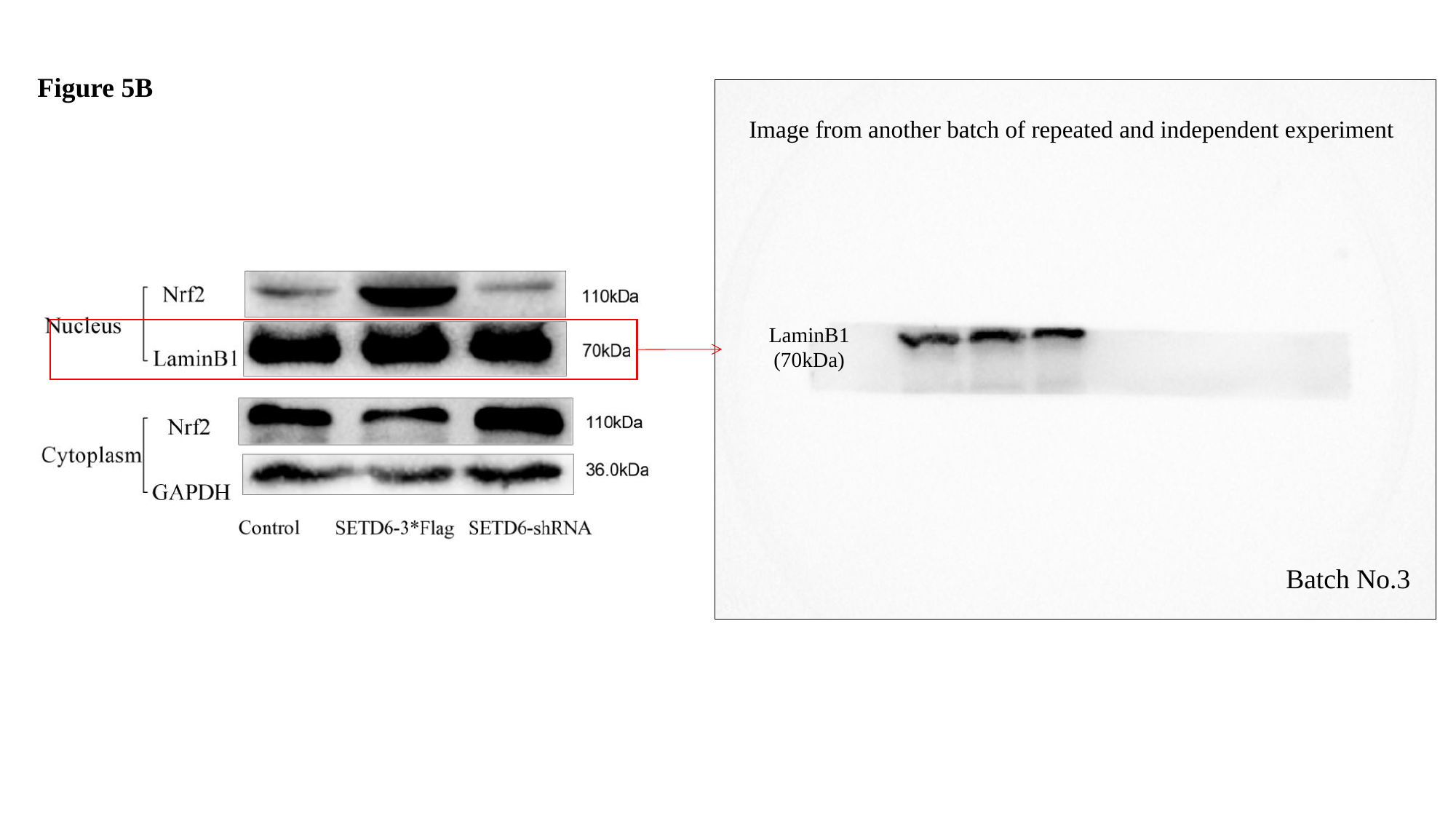

Figure 5B
Image from another batch of repeated and independent experiment
LaminB1
(70kDa)
Batch No.3

## Slide 39
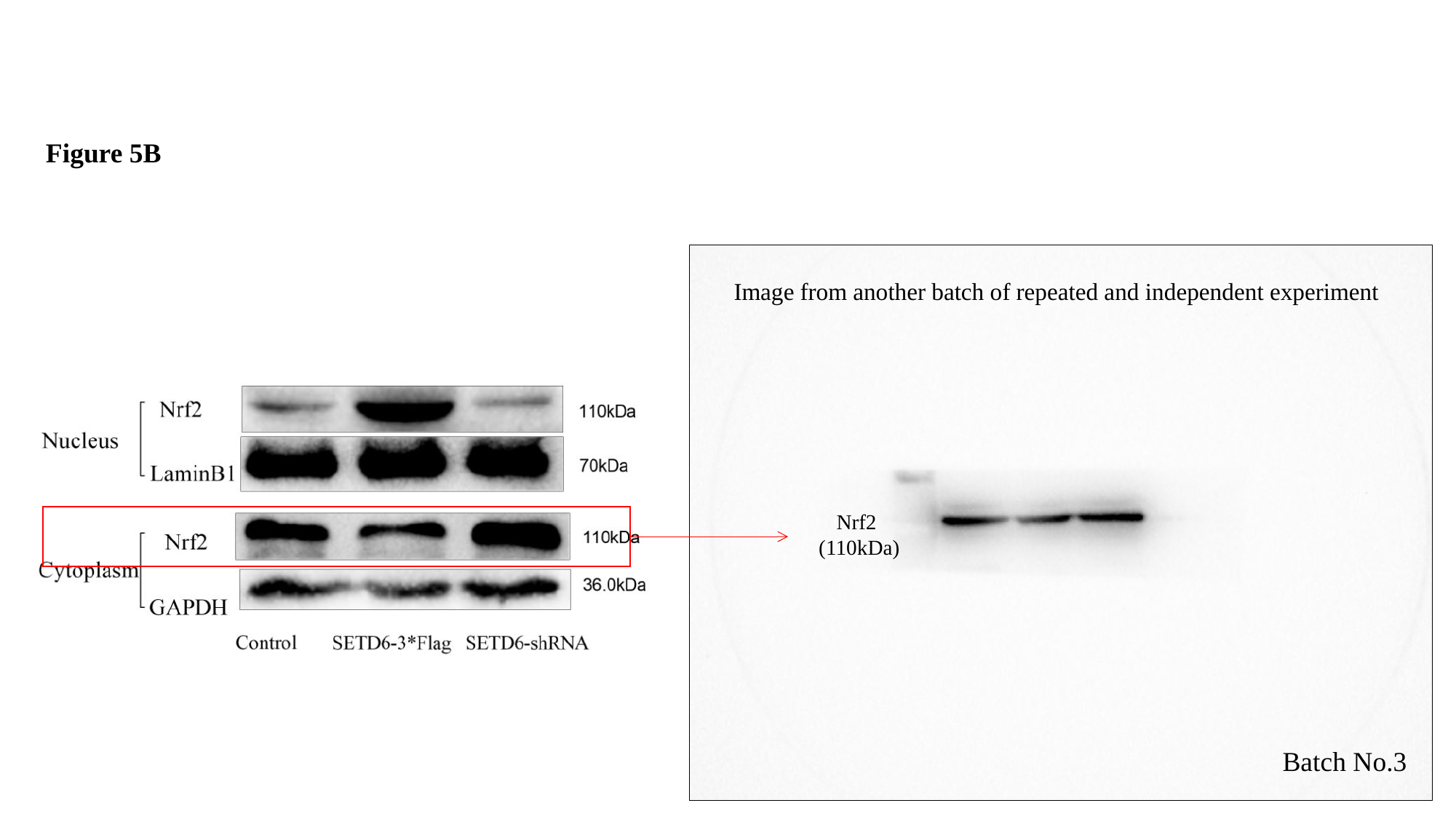

Figure 5B
Image from another batch of repeated and independent experiment
Nrf2
(110kDa)
Batch No.3

## Slide 40
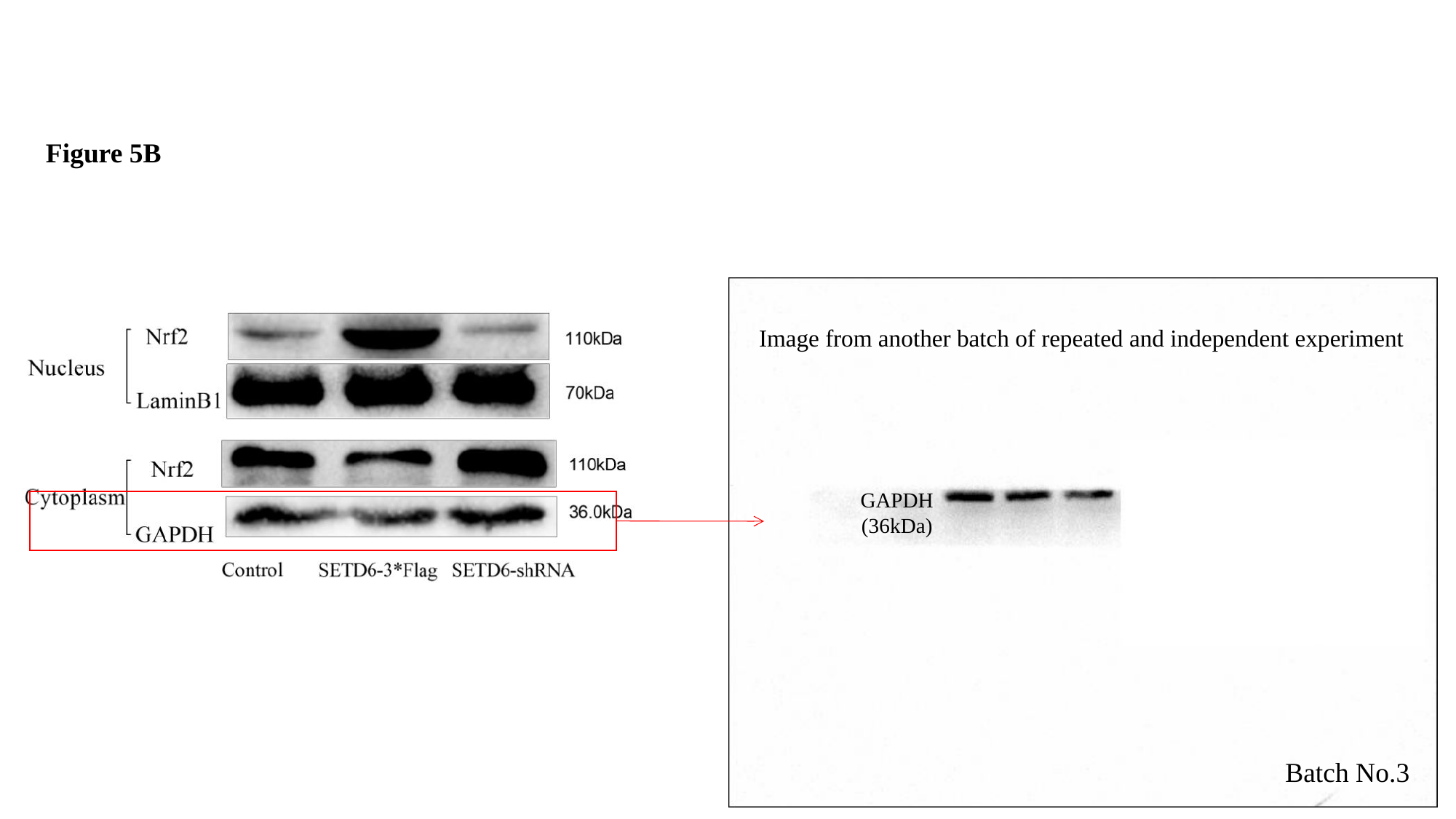

Figure 5B
Image from another batch of repeated and independent experiment
GAPDH
(36kDa)
Batch No.3

## Slide 41
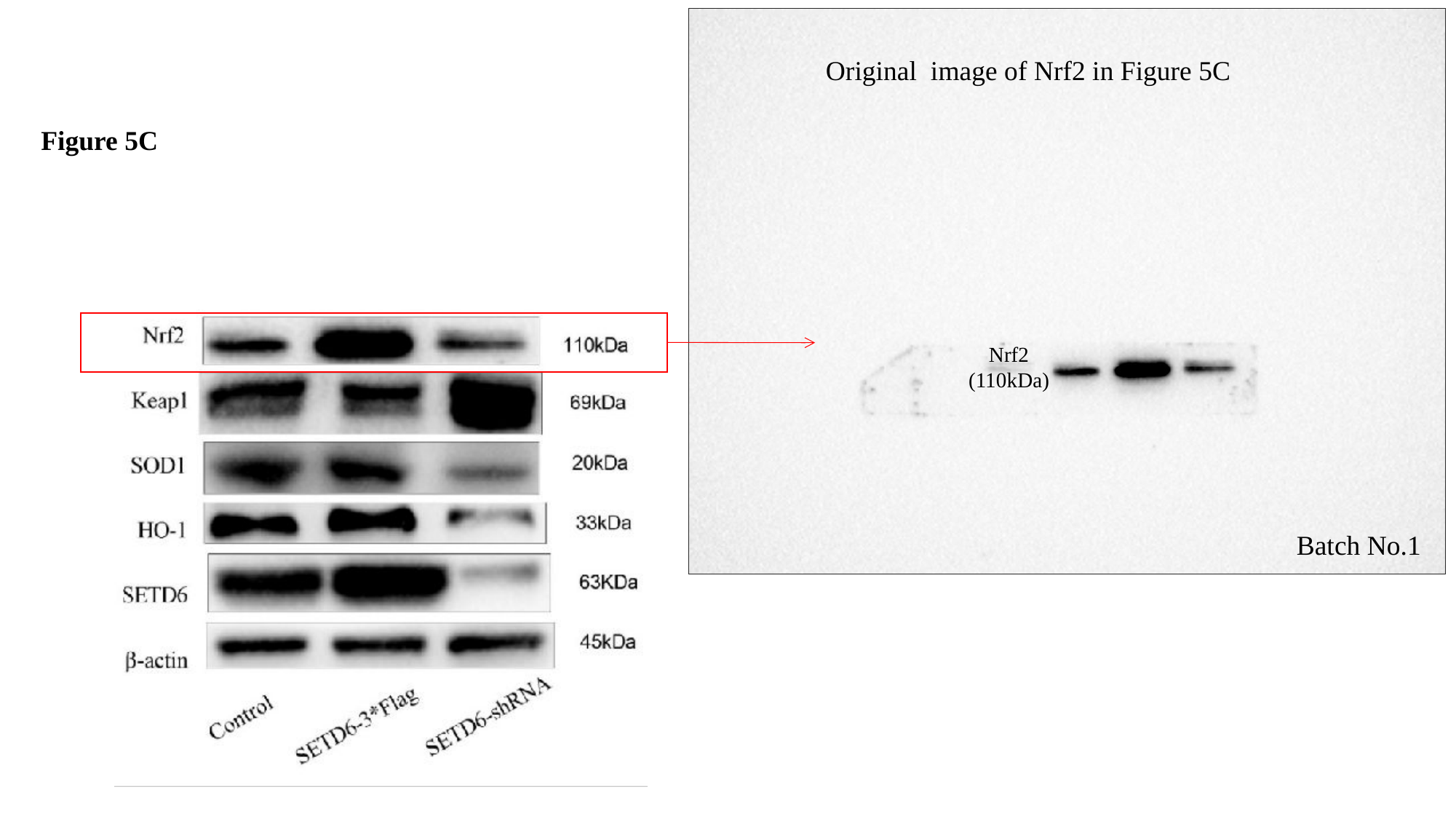

Original image of Nrf2 in Figure 5C
Figure 5C
Nrf2
(110kDa)
Batch No.1

## Slide 42
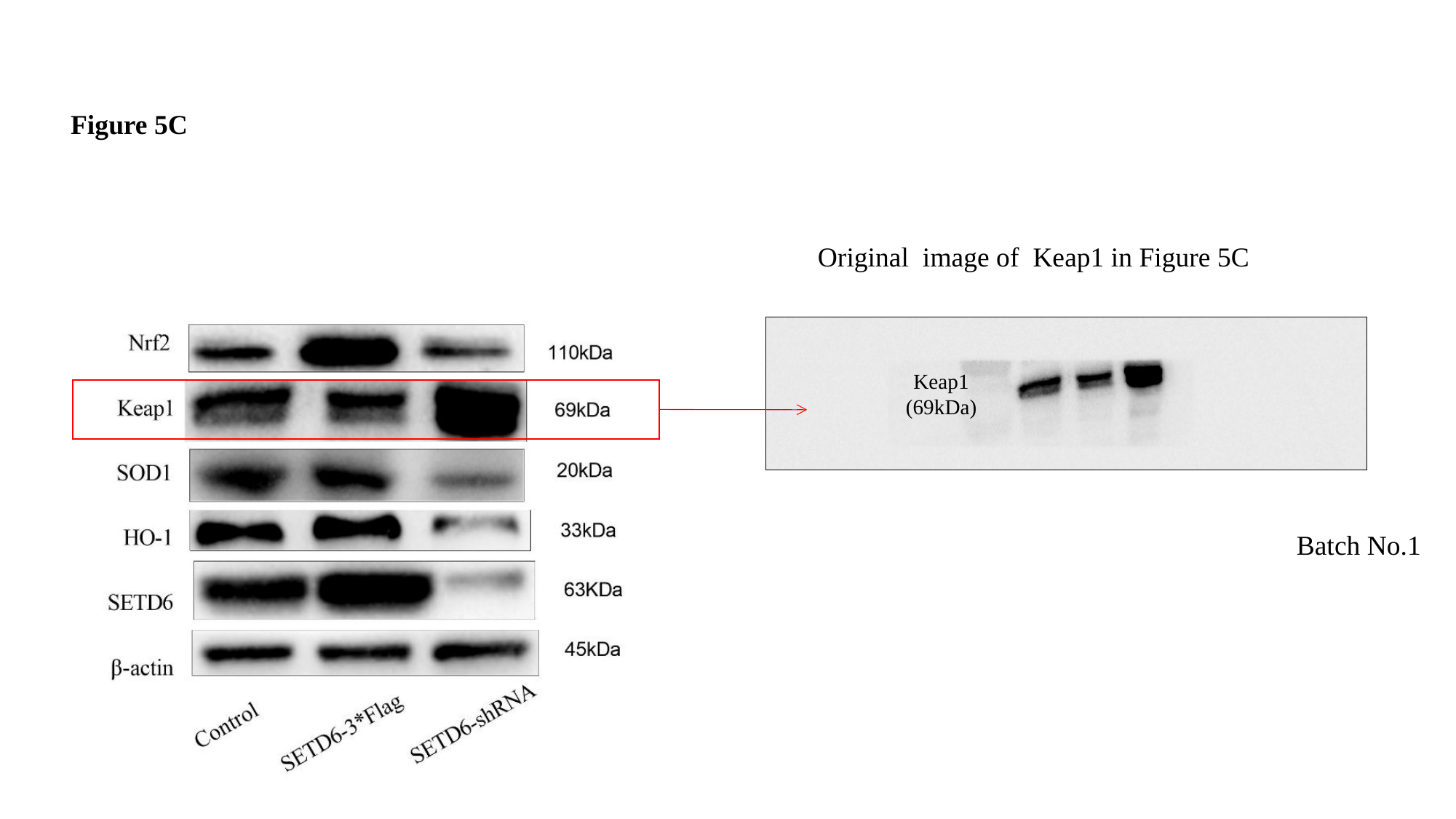

Figure 5C
Original image of Keap1 in Figure 5C
Keap1 (69kDa)
Batch No.1

## Slide 43
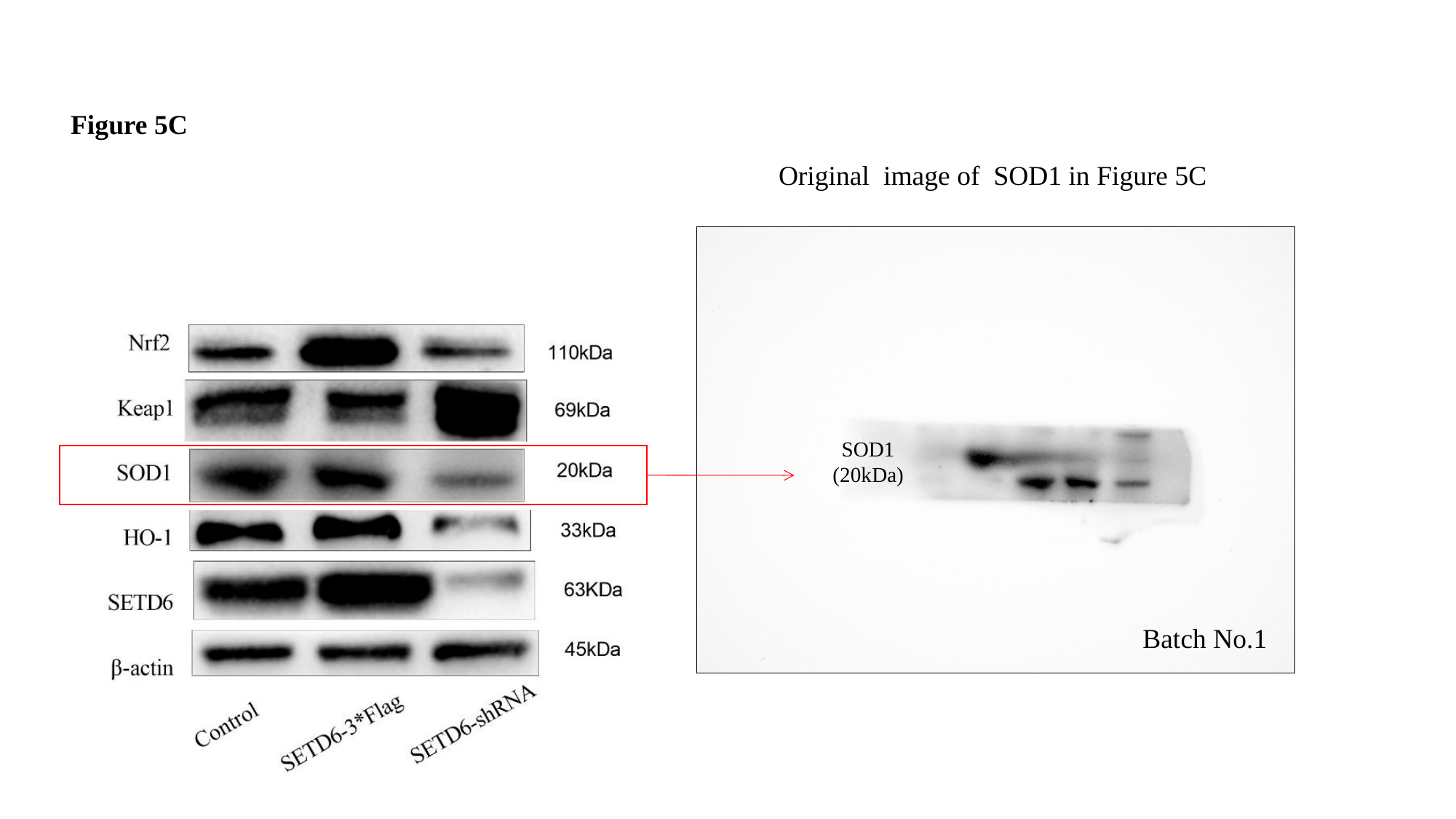

Figure 5C
Original image of SOD1 in Figure 5C
SOD1 (20kDa)
Batch No.1

## Slide 44
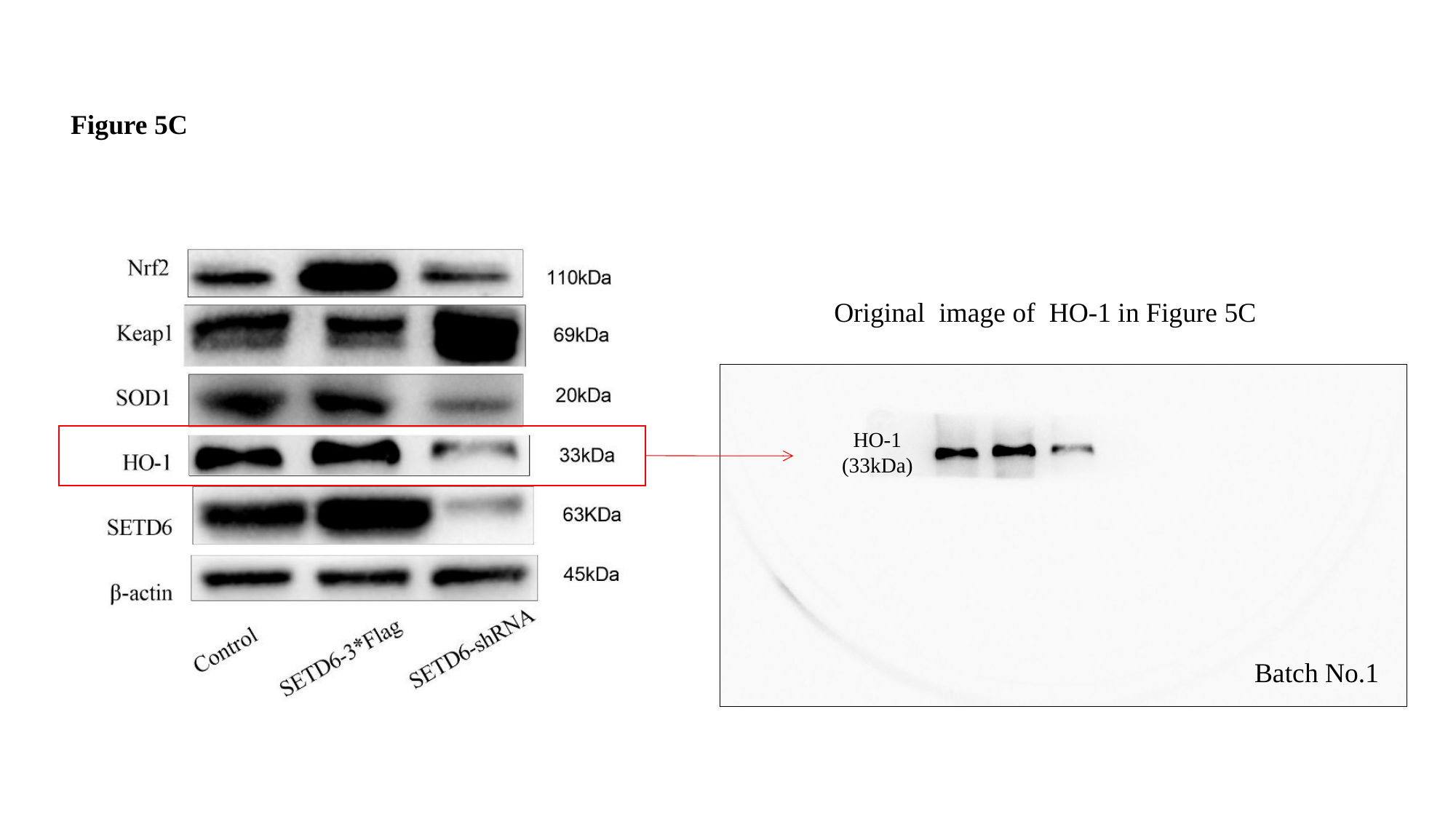

Figure 5C
Original image of HO-1 in Figure 5C
HO-1 (33kDa)
Batch No.1

## Slide 45
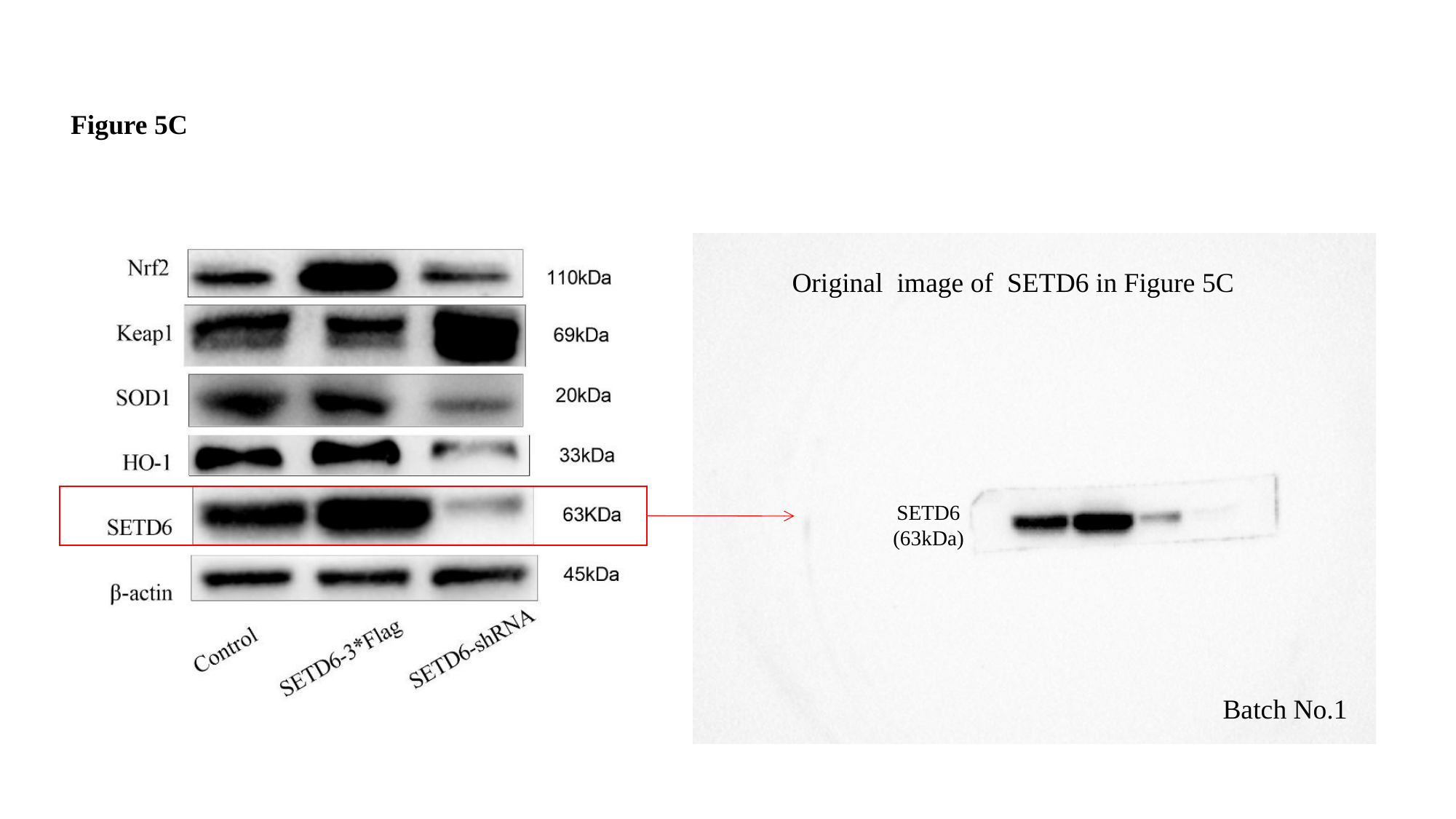

Figure 5C
Original image of SETD6 in Figure 5C
SETD6
(63kDa)
Batch No.1

## Slide 46
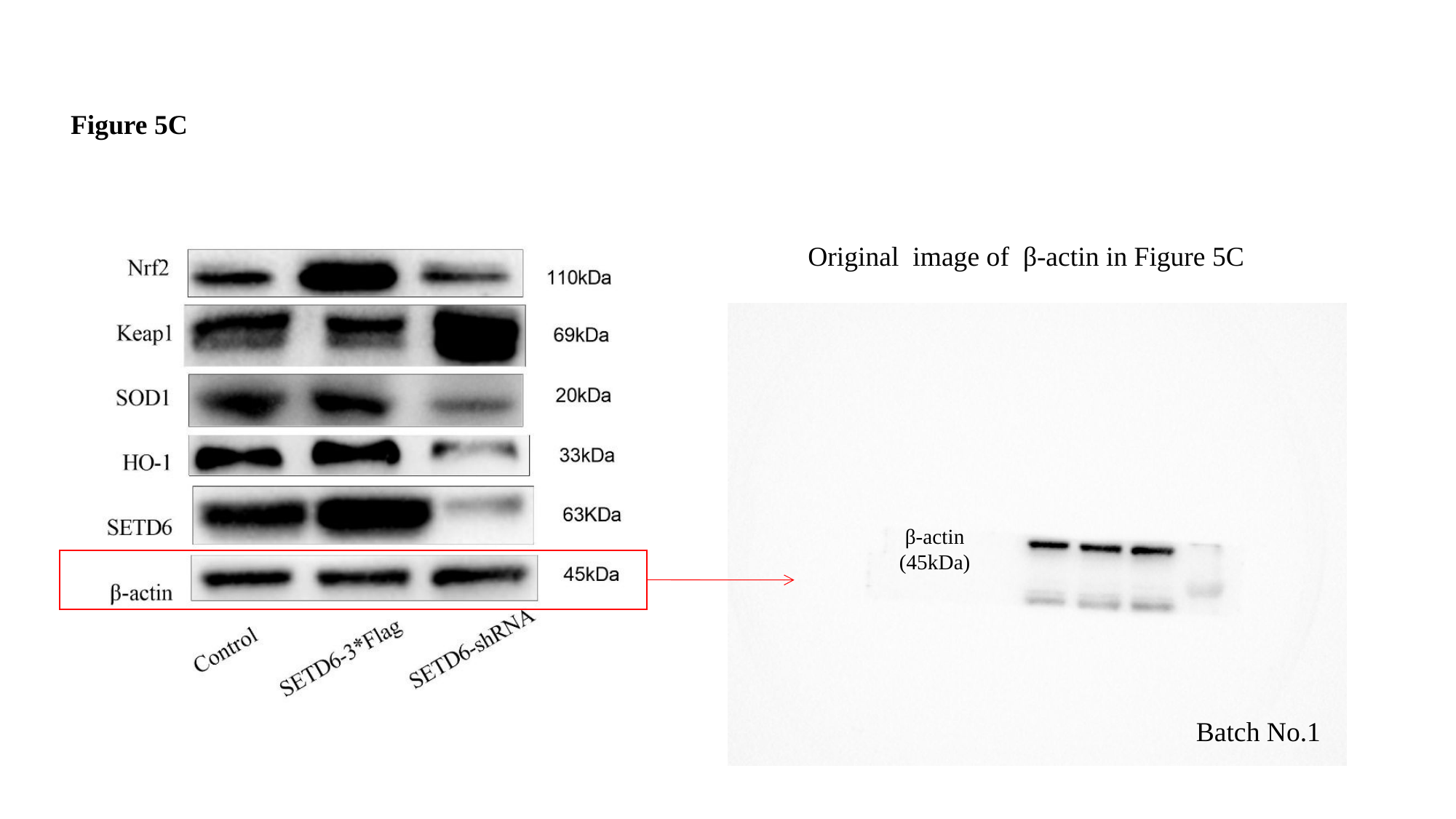

Figure 5C
Original image of β-actin in Figure 5C
β-actin
(45kDa)
Batch No.1

## Slide 47
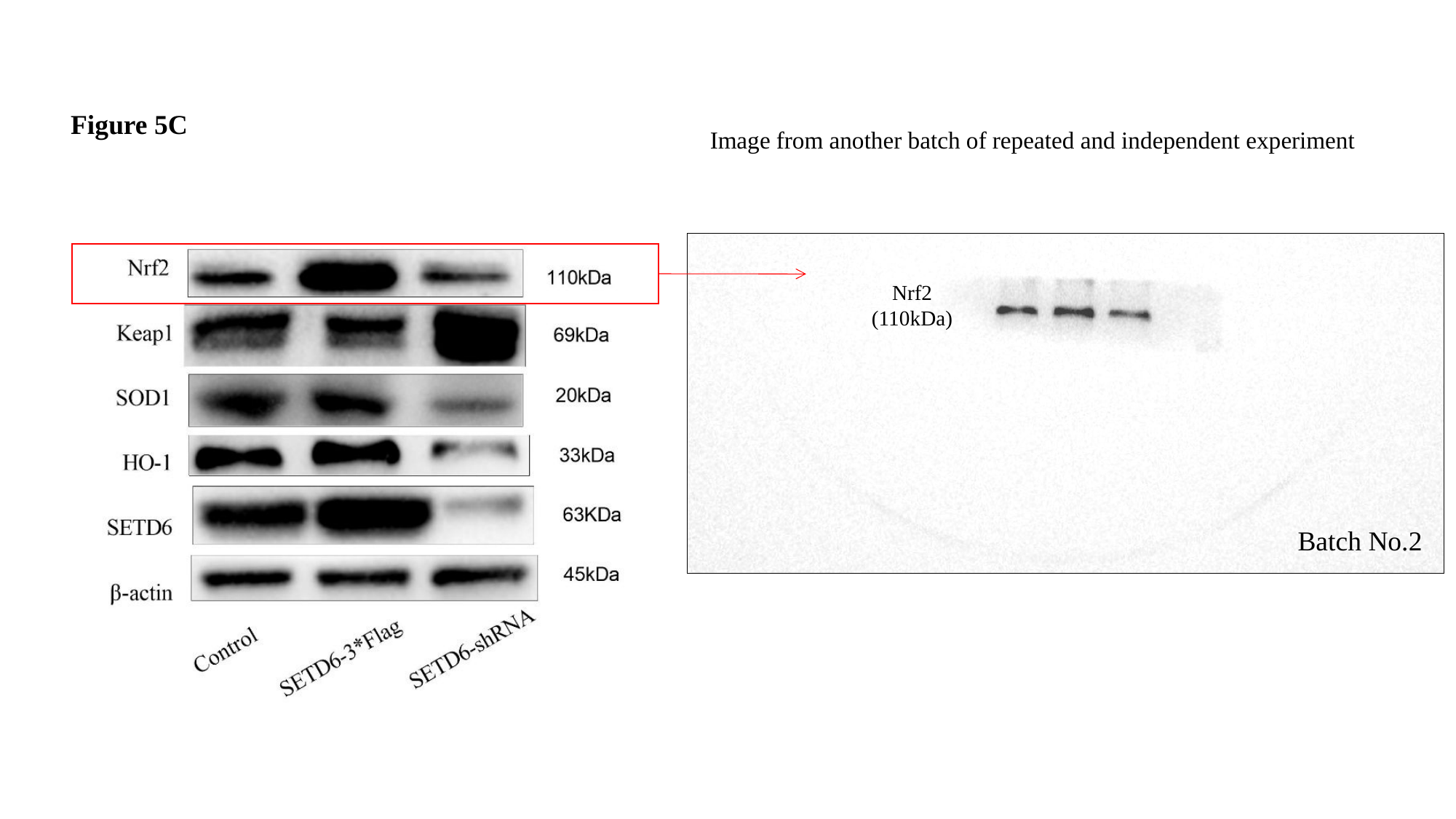

Figure 5C
Image from another batch of repeated and independent experiment
Nrf2
(110kDa)
Batch No.2

## Slide 48
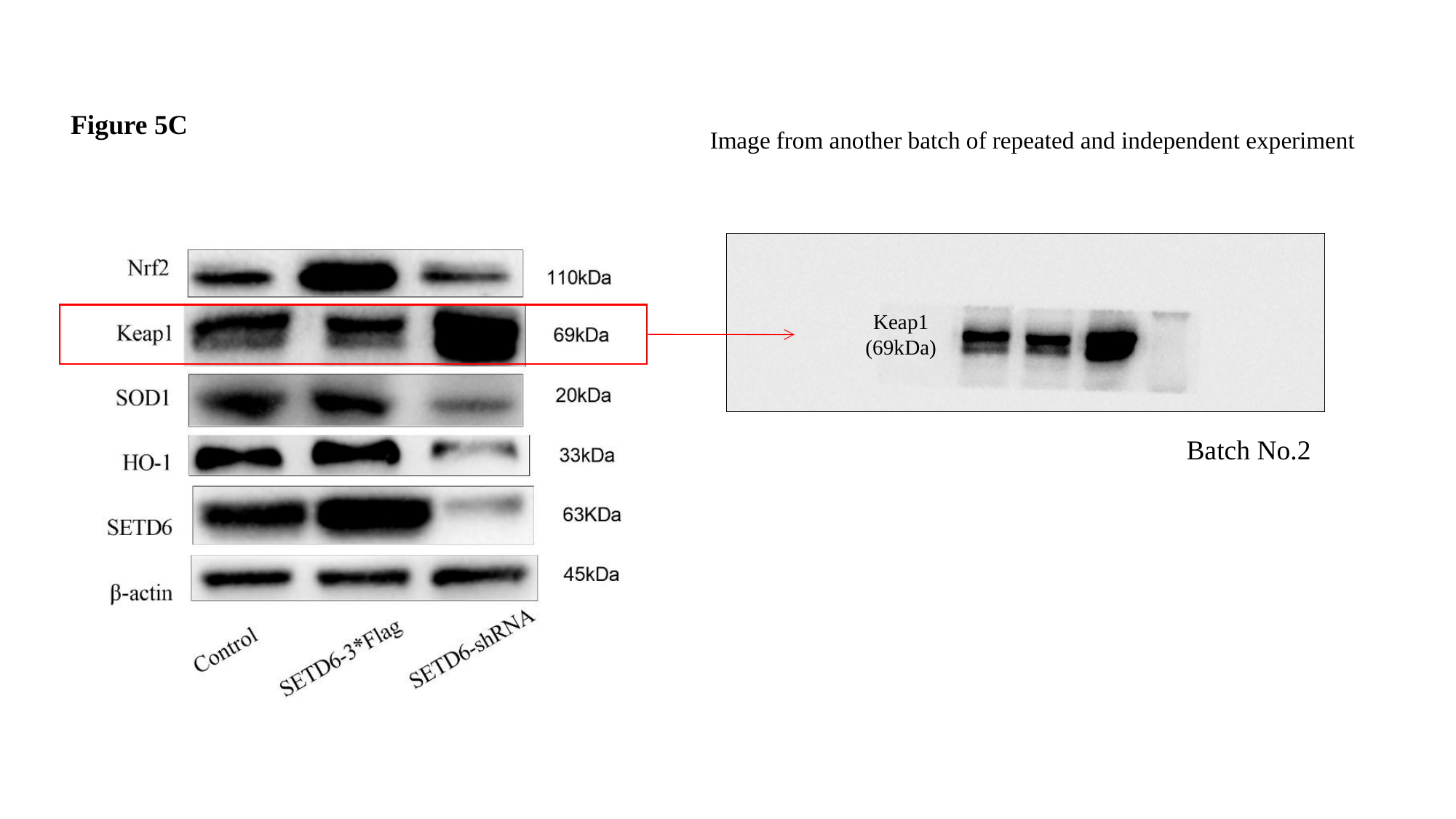

Figure 5C
Image from another batch of repeated and independent experiment
Keap1
(69kDa)
Batch No.2

## Slide 49
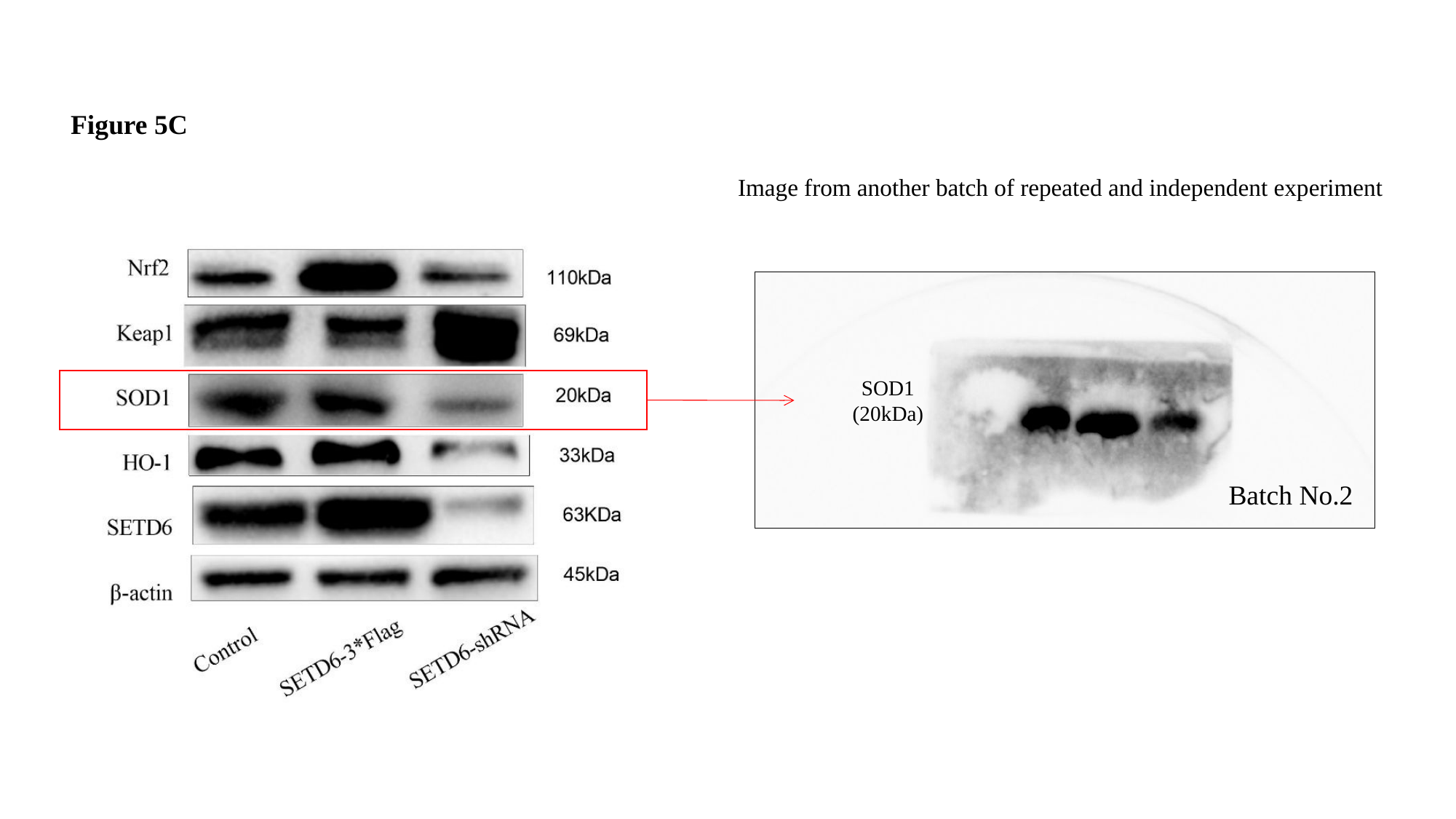

Figure 5C
Image from another batch of repeated and independent experiment
SOD1
(20kDa)
Batch No.2

## Slide 50
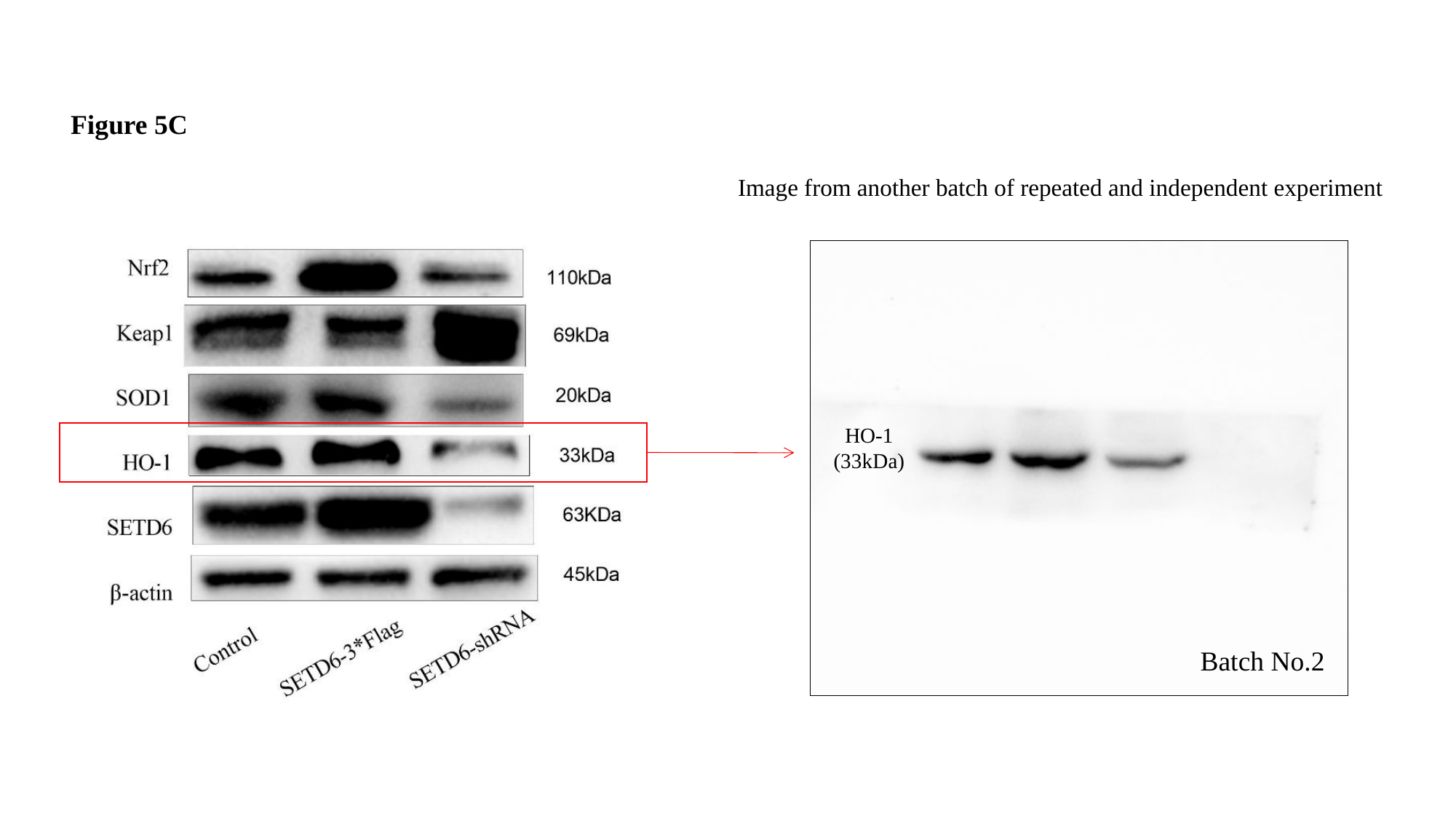

Figure 5C
Image from another batch of repeated and independent experiment
HO-1
(33kDa)
Batch No.2

## Slide 51
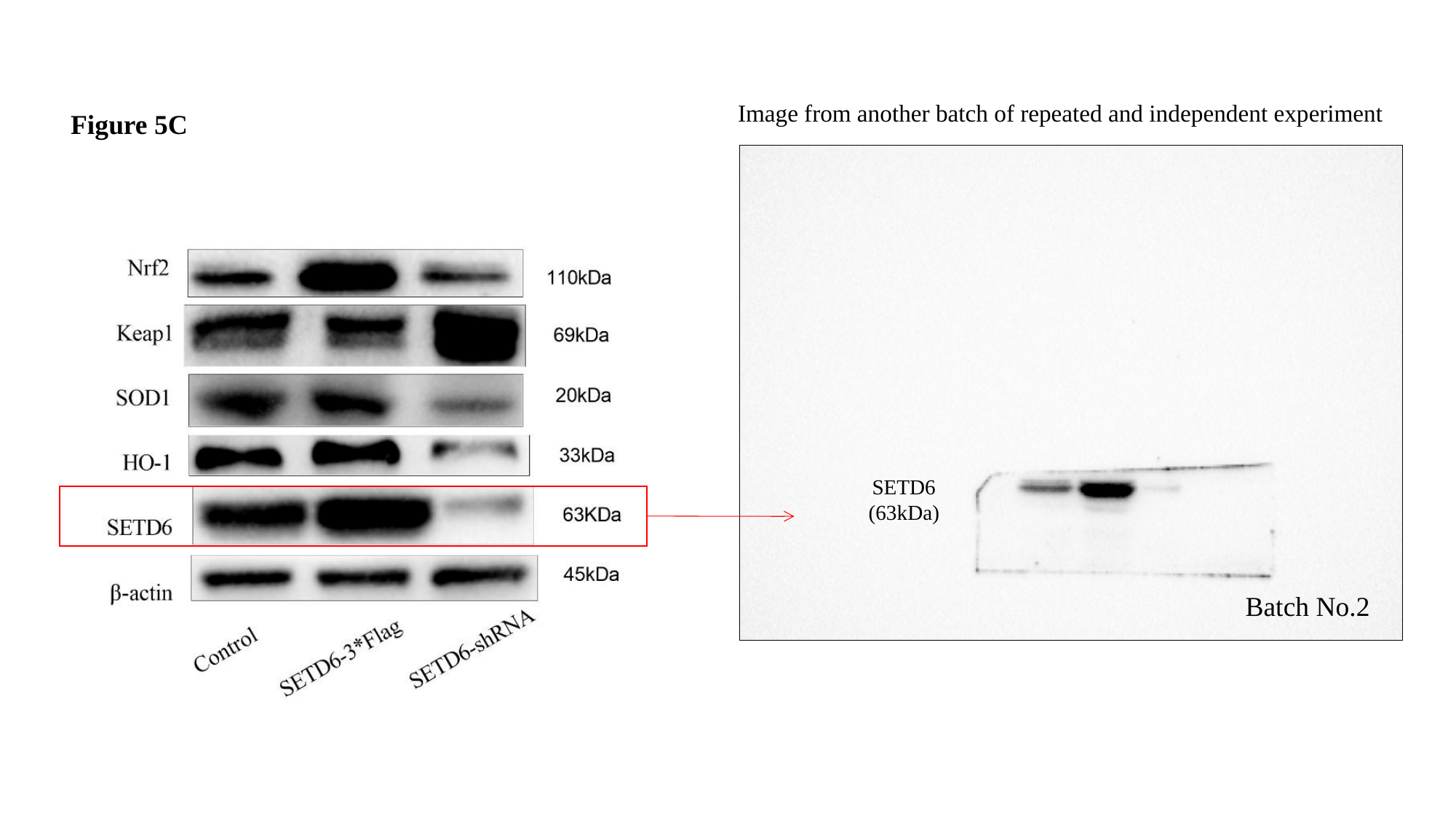

Image from another batch of repeated and independent experiment
Figure 5C
SETD6
(63kDa)
Batch No.2

## Slide 52
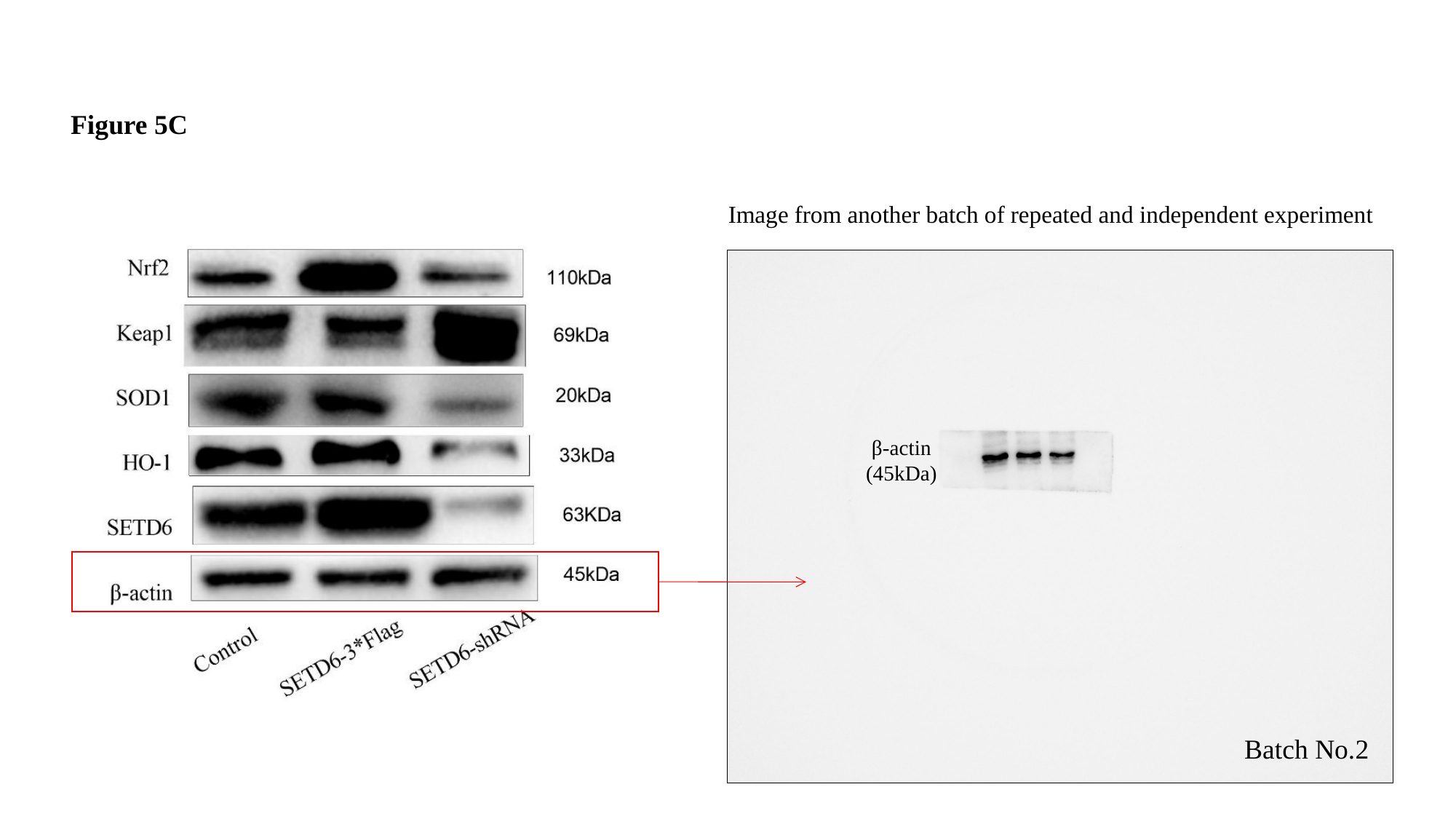

Figure 5C
Image from another batch of repeated and independent experiment
β-actin
(45kDa)
Batch No.2

## Slide 53
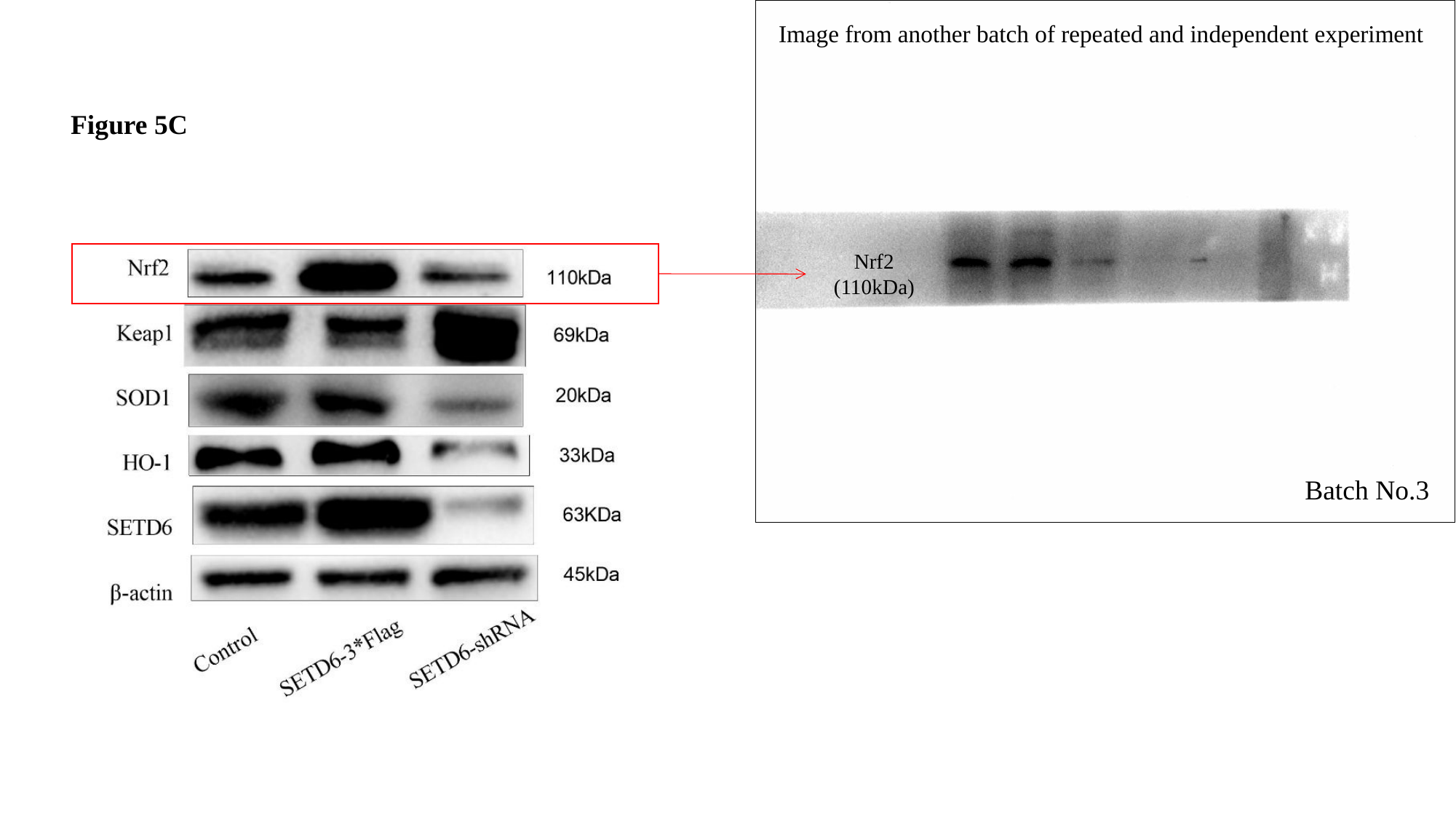

Image from another batch of repeated and independent experiment
Figure 5C
Nrf2
(110kDa)
Batch No.3

## Slide 54
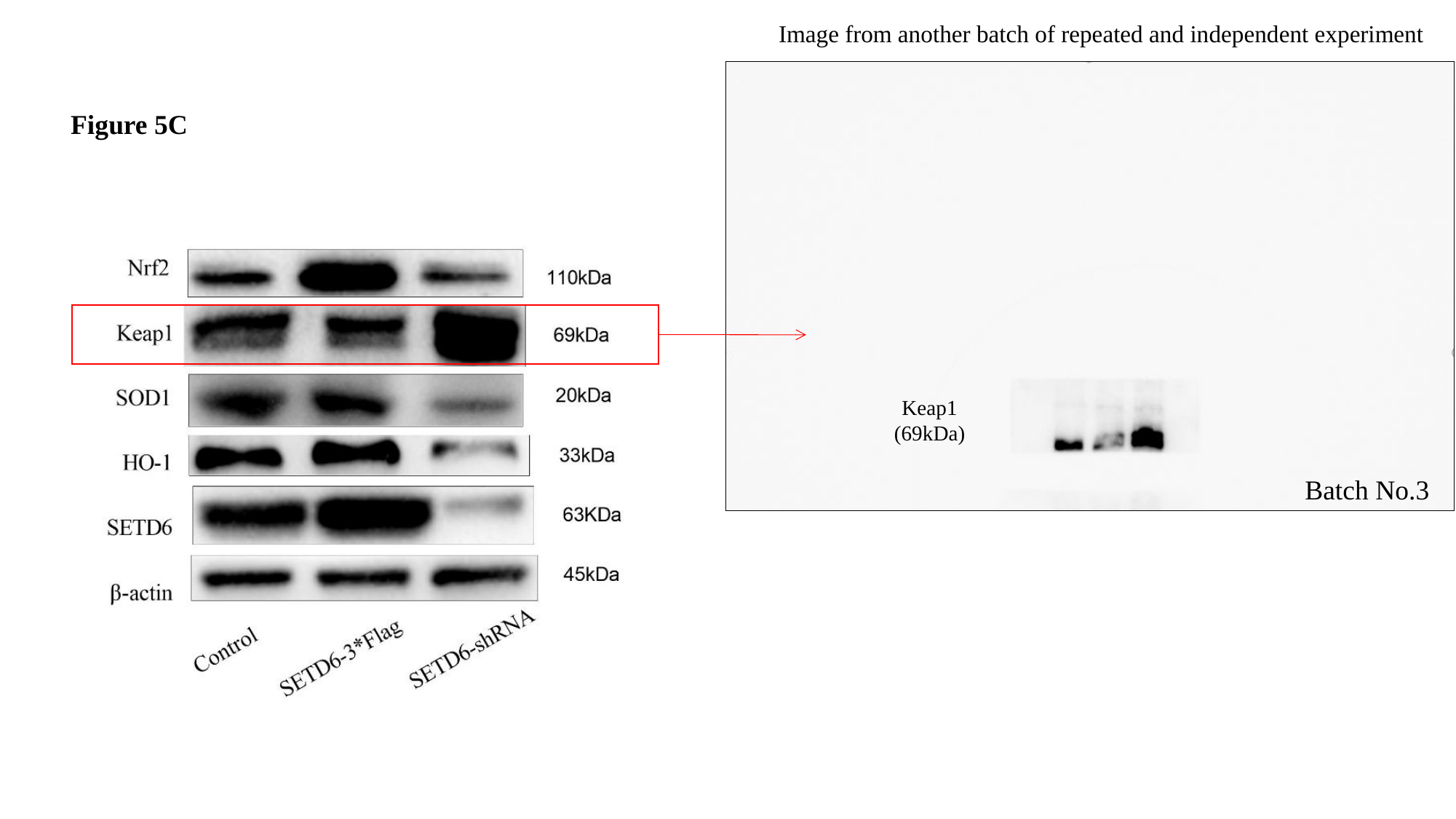

Image from another batch of repeated and independent experiment
Figure 5C
Keap1
(69kDa)
Batch No.3

## Slide 55
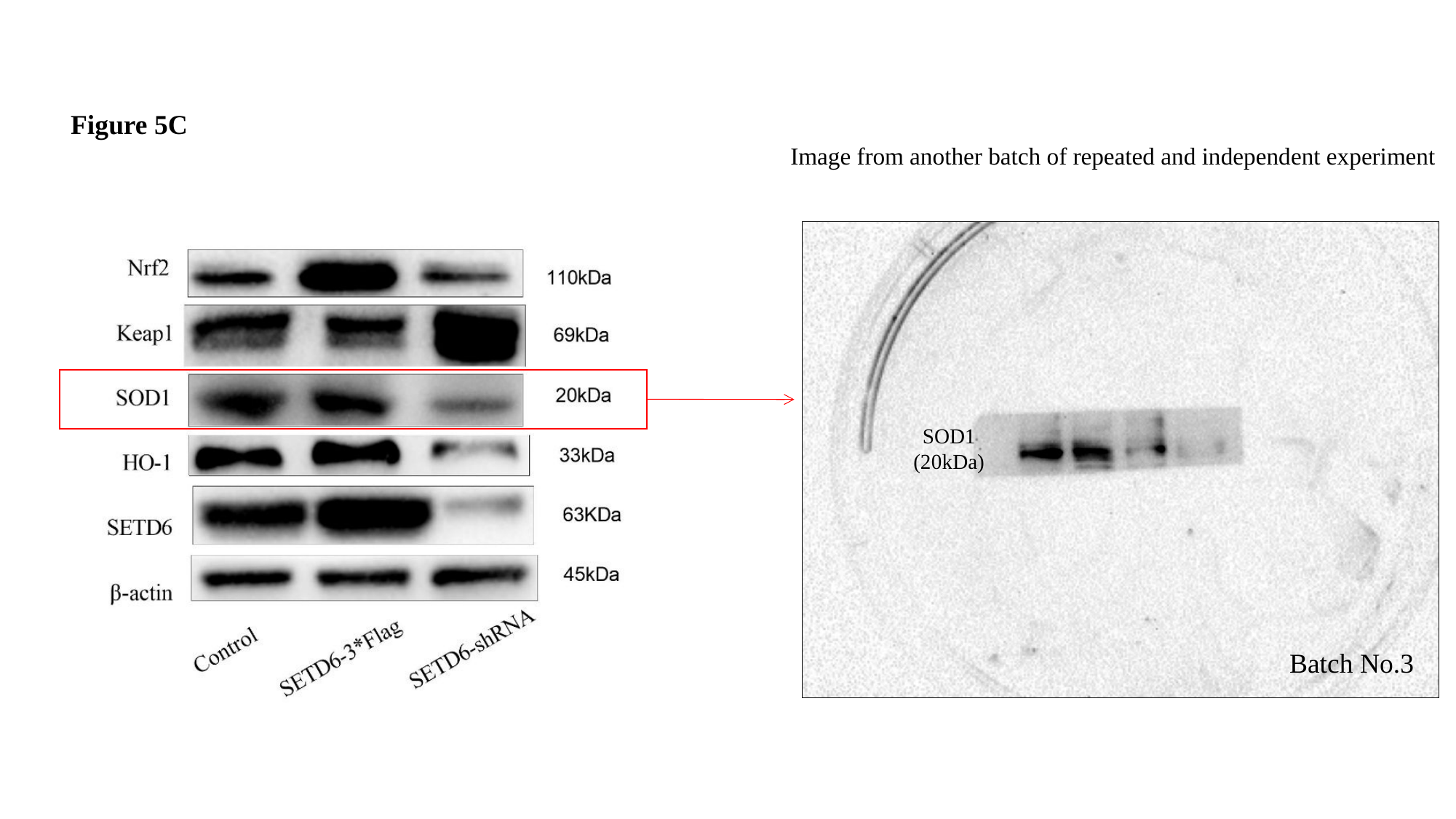

Figure 5C
Image from another batch of repeated and independent experiment
SOD1
(20kDa)
Batch No.3

## Slide 56
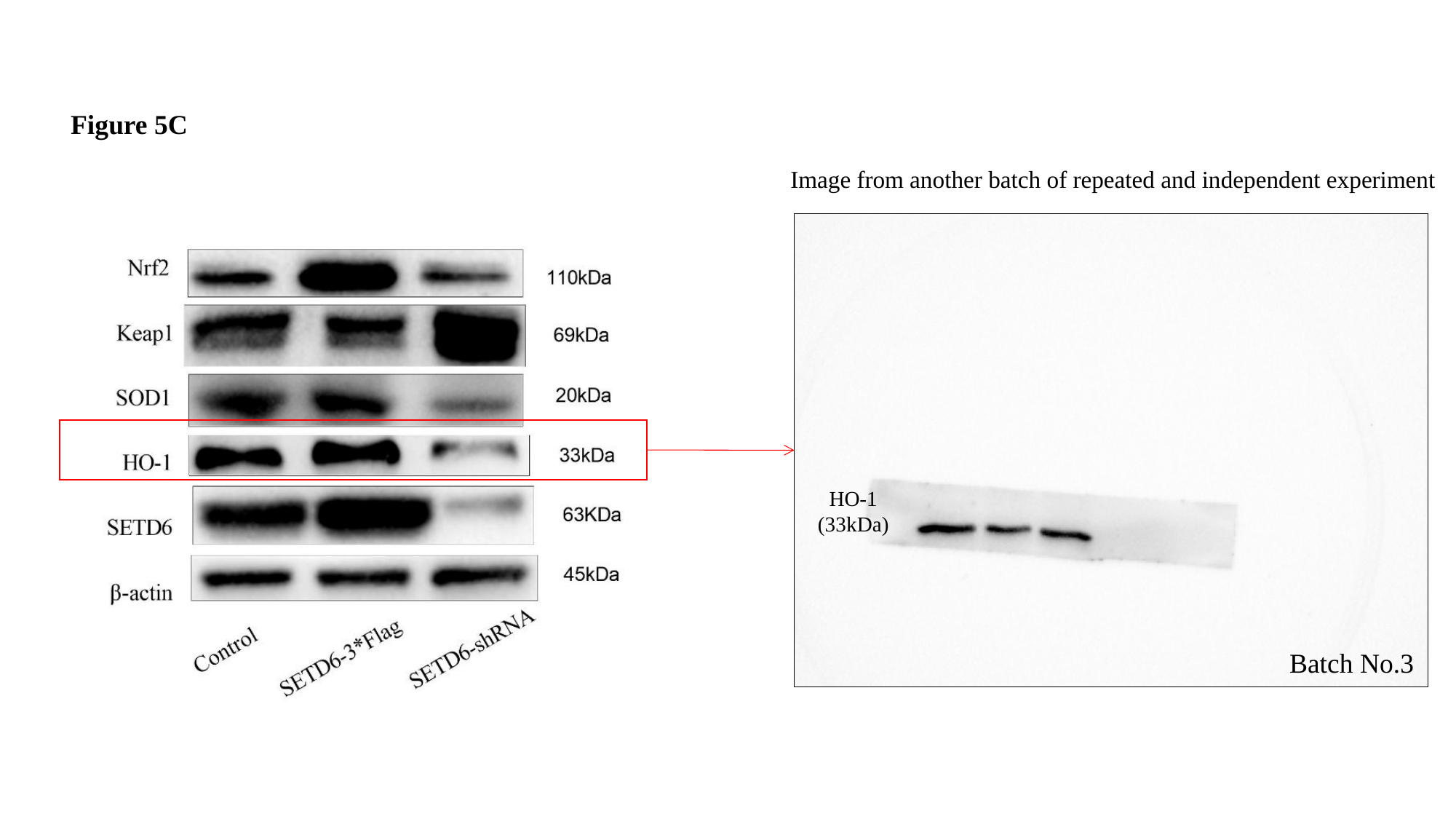

Figure 5C
Image from another batch of repeated and independent experiment
HO-1
(33kDa)
Batch No.3

## Slide 57
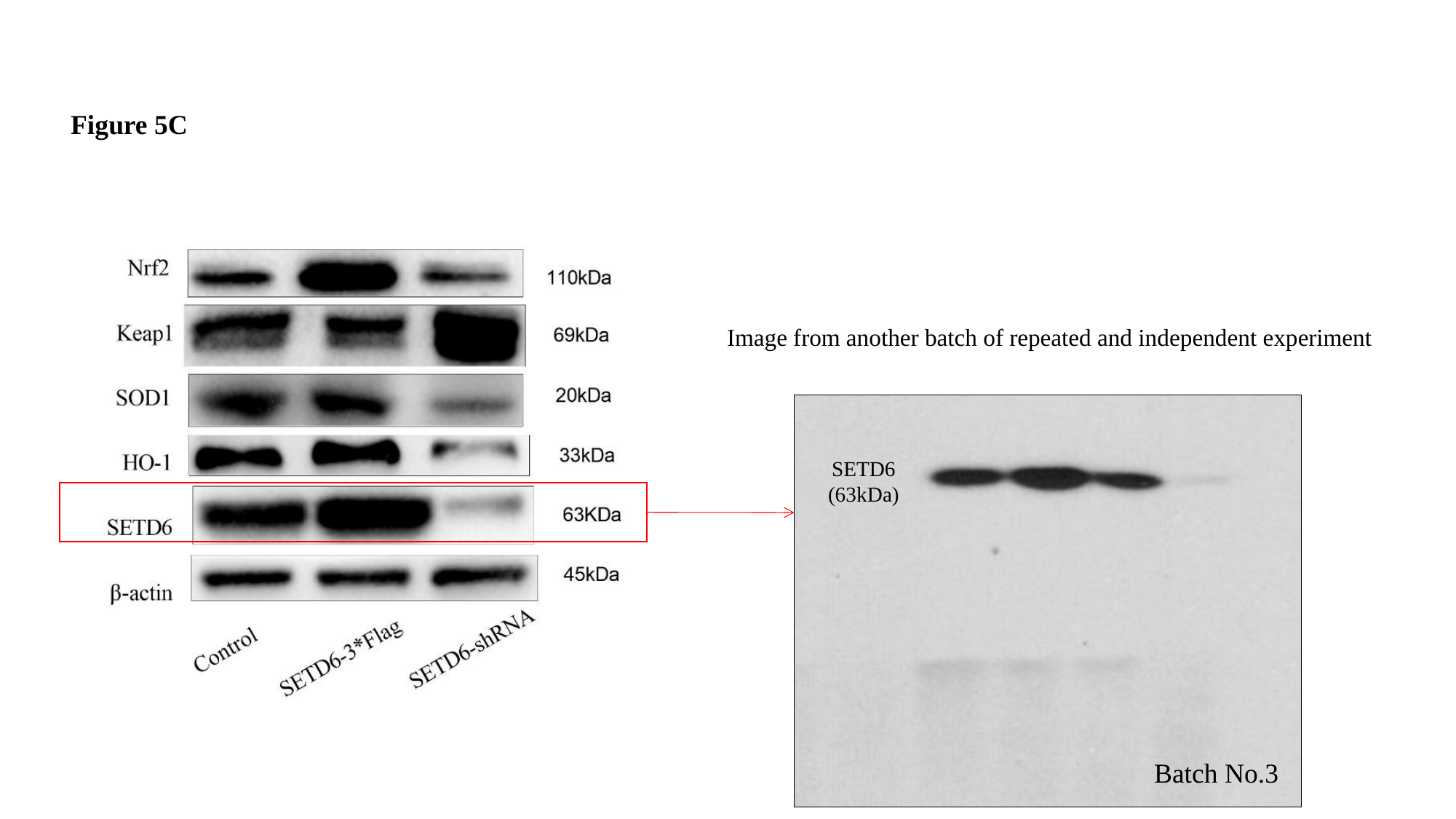

Figure 5C
Image from another batch of repeated and independent experiment
SETD6
(63kDa)
Batch No.3

## Slide 58
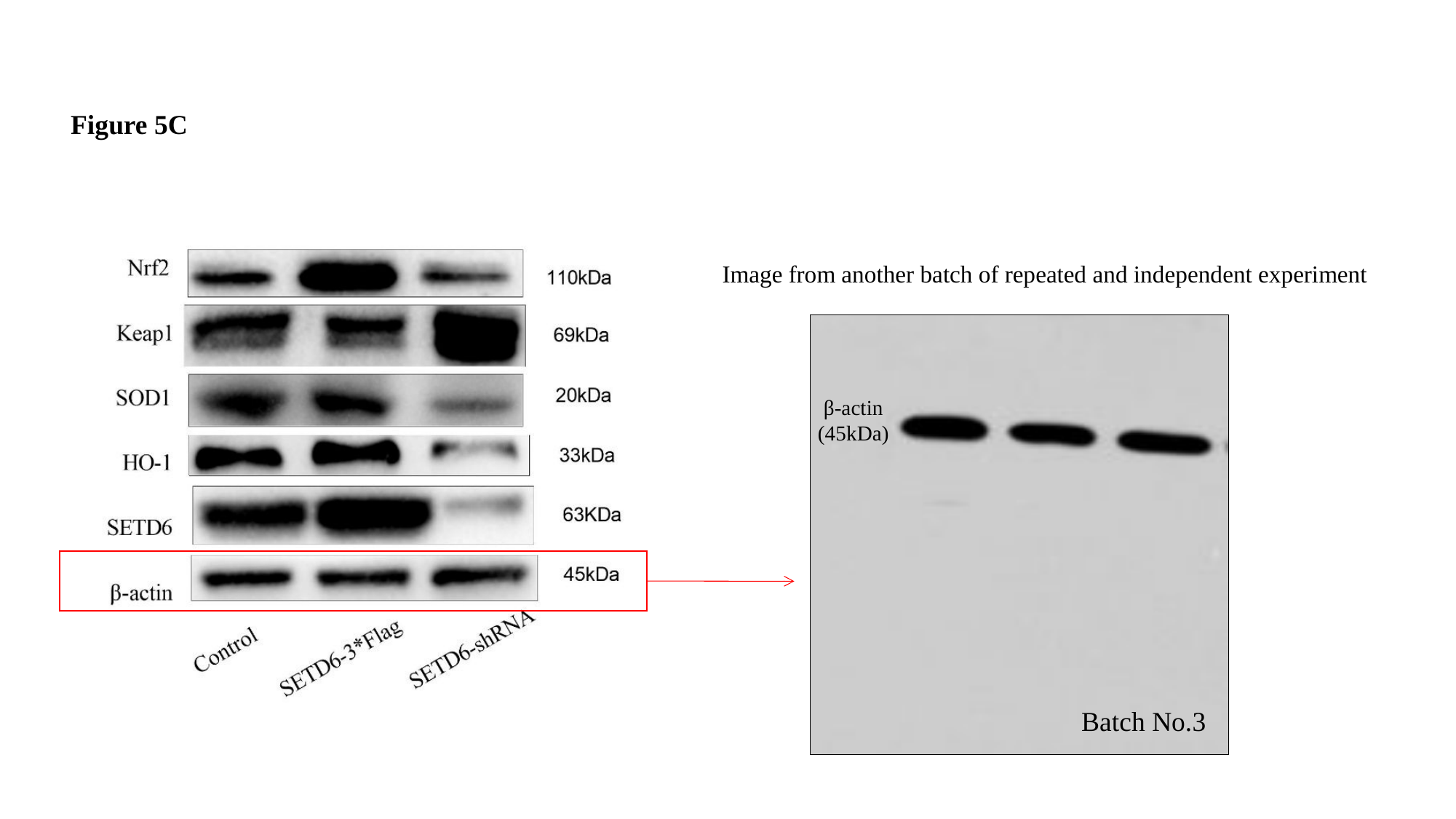

Figure 5C
Image from another batch of repeated and independent experiment
β-actin
(45kDa)
Batch No.3
